# Asymmetric Triple Helical Nonbenzenoid Nanographenes with Controllable Helicene Lengths

**DOI:** 10.1021/acs.orglett.5c03372

**Published:** 2025-09-05

**Authors:** Lin Yang, Yucheng Yin, Wenhui Niu, Yubin Fu, Fupin Liu, Hartmut Komber, Alexey A. Popov, Ji Ma, Xinliang Feng

**Affiliations:** † Max Planck Institute of Microstructure Physics, Weinberg 2, 06120 Halle, Germany; ‡ Centre for Advancing Electronics Dresden (cfaed), Department of Chemistry and Food Chemistry, 9169 Technische Universität Dresden, 01062 Dresden, Germany; § State Key Laboratory of Synergistic Chem-Bio Synthesis, School of Chemistry and Chemical Engineering, Frontiers Science Center for Transformative Molecules, Shanghai Key Laboratory of Electrical Insulation and Thermal Ageing, Shanghai Jiao Tong University, Shanghai 200240, China; ∥ Leibniz Institute for Solid State and Materials Research, Helmholtzstraße 20, 01069 Dresden, Germany; ⊥ Jiangsu Key Laboratory of New Power Batteries, Jiangsu Collaborative Innovation Center of Biomedical Functional Materials, School of Chemistry and Materials Science, 12534 Nanjing Normal University, 210023 Nanjing, China; # 28408 Leibniz-Institut für Polymerforschung Dresden e.V., Hohe Straße 6, 01069 Dresden, Germany; ∇ College of Materials Science and Optoelectronic Technology & Center of Materials Science and Optoelectronics Engineering, University of Chinese Academy of Science, 100049 Beijing, P. R. China

## Abstract

Helical nanographenes (NGs) play a crucial role in the development of chiral nanomaterials due to their distinctive optoelectronic and chiroptical properties. Herein, we report the efficient synthesis of two unprecedented azulene-embedded asymmetric triple helical NGs (**1** and **2**) with controllable helicene subunit lengths and π-extension. The crystallographic analysis confirms their highly twisted and asymmetric geometries. Both compounds exhibit narrow and tunable optical energy gaps (**1**: 1.74 eV; **2**: 1.63 eV), as determined by UV–vis absorption and cyclic voltammetry measurements. Moreover, the enantiomers of **1** display pronounced chiroptical activity with a *g*_abs_ of up to 3.1 × 10^–3^.

## Graphical abstract

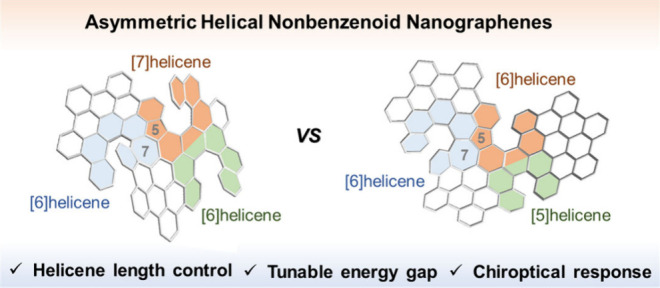

The development of chiral nanomaterials has emerged as a pivotal frontier in materials science, driven by their unique optical, electronic, and magnetic properties.^1–5^ Among these, triple helical nanographenes (THNs), which incorporate three individual helicene units, represent a structurally complex and functionally versatile class of molecules. The 3-fold helicene subunits endow these systems with inherent chirality, high racemization barrier, and pronounced chiroptical responses.^6–10^ In recent years, substantial progress has been made in the synthesis of symmetric THNs through the integration of triple [n]­helicene units into polycyclic aromatic frameworks (A–D in Figure 1a).^11–21^ In contrast, introducing symmetry breaking into THNs enables further regulation of their geometry and electronic structures, leading to notable changes in molecular strain and physicochemical properties.^22–27^ Despite this potential, the synthesis of asymmetric triple helical nanographenes (ATHNs) remains largely underexplored, primarily due to the scarcity of suitable precursors and the absence of versatile synthetic strategies.

**Figure 1:**
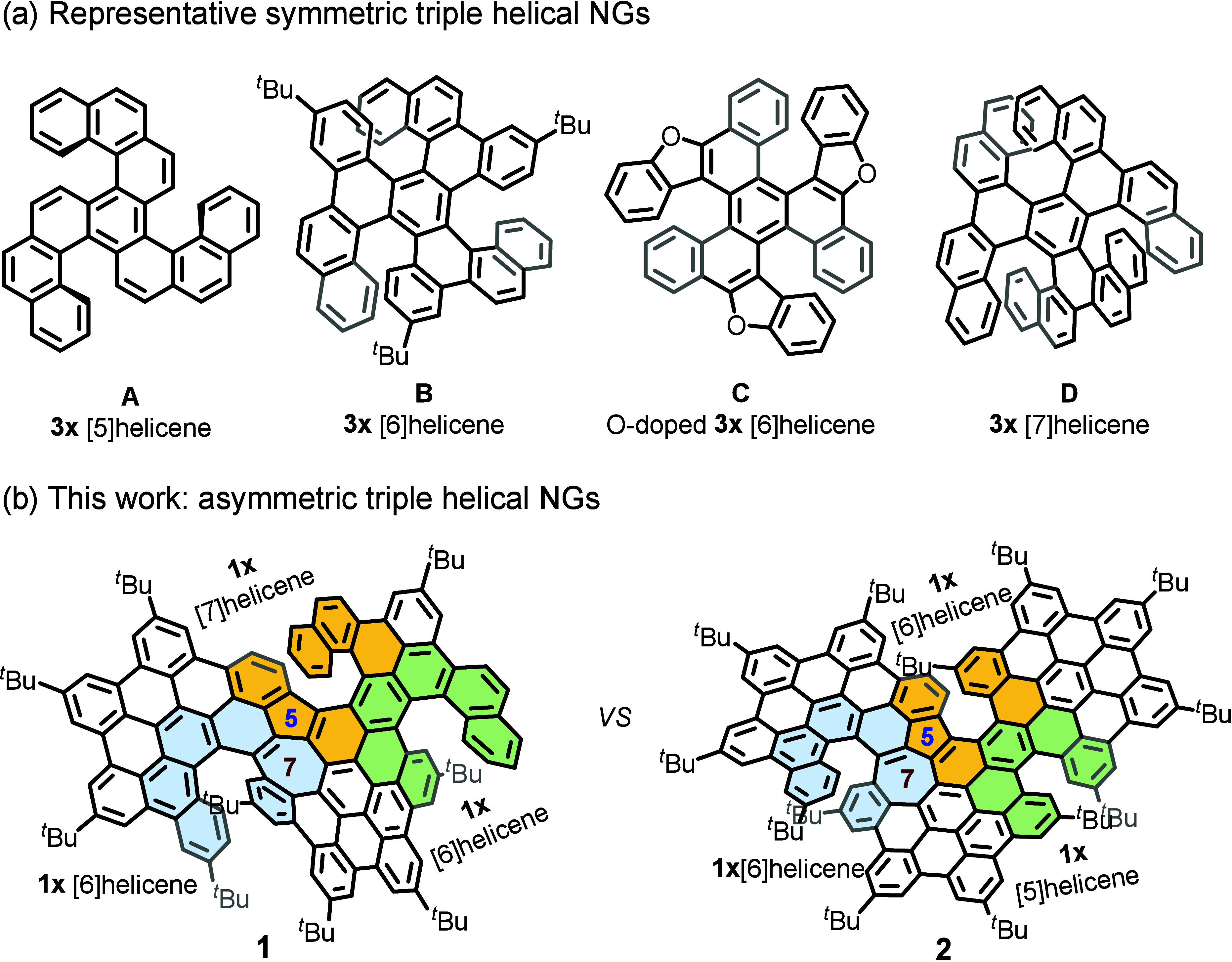
Chemical structure of triple helical NGs. (a) Representative symmetric triple helical NGs. (b) Asymmetric triple helical NGs in this work.

In this letter, we report an efficient and straightforward synthesis of two unprecedented azulene-embedded ATHNs (**1** and **2**, Scheme 1), accessed from the Scholl-type cyclization of the tailored azulene-embedded nonbenzenoid precursors. The obtained compound **1** incorporates a [6]­helicene, a heptagon-embedded [6]­helicene and a pentagon-embedded [7]­helicene, while **2** features a [5]­helicene, a heptagon-embedded [6]­helicene and a pentagon-embedded [6]­helicene along its backbone (Figure 1b). Single-crystal X-ray diffraction unambiguously confirms their unique asymmetric triple-helical structures. Detailed analysis of their crystal structures reveals significant molecular strain and highly twisted geometries resulting from the asymmetrical arrangement of the helicene subunits. The fundamental physicochemical properties of both compounds were thoroughly investigated using UV–vis absorption and cyclic voltammetry (CV), demonstrating their narrow energy gaps (1.74 eV for **1**; 1.63 eV for **2**). Furthermore, chiral high-performance liquid chromatography (HPLC) was used to resolve the enantiomers of **1**, and their chiroptical properties were examined by electronic circular dichroism (ECD). This study offers valuable insights into how helicene subunits impact the fine-tuning of topology and optoelectronic properties in triple helical NGs.

**Scheme 1:**
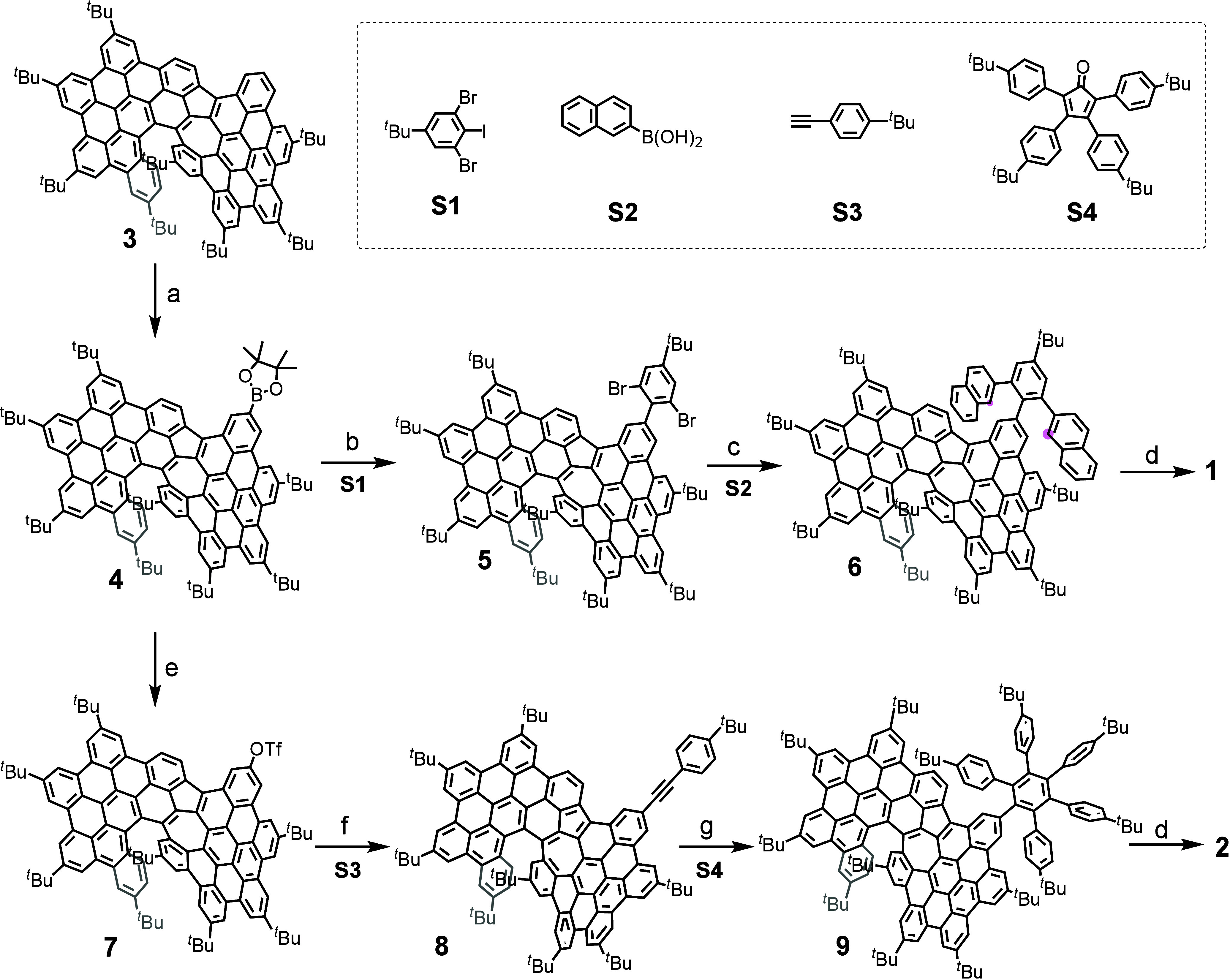
Synthetic Routes to **1** and **2** ^a^

a Reaction conditions: a) B_2_pin_2_, [Ir­(OMe)­(cod)]_2_, dtbpy, cyclohexane, 100 °C, 16 h. b) Pd­(dppf)­Cl_2_·CH_2_Cl_2_, K_2_CO_3_, THF/H_2_O, 80 °C, 24 h; 61% over two steps. c) Pd­(PPh_3_)_4_, K_2_CO_3_, THF/H_2_O, 85 °C, 18 h, 92%. d) DDQ, TfOH, CH_2_Cl_2_, 0 °C, 20 min, 43% for **1** or 68% for **2**. e) i. NaOH, H_2_O_2_, THF/H_2_O, rt, 12 h; ii. Tf_2_O, pyridine, DCM, 0 °C, 1 h, then rt, 12 h; 68% over two steps. f) Pd­(PPh_3_)_2_Cl_2_, CuI, Et_3_N, THF, 70 °C, 12 h, 89%. g) Ph_2_O, 265 °C, 36 h, 87%.

The synthetic routes to azulene-embedded ATHNs **1** and **2** are depicted in Scheme 1. Compound **3**, a key starting material reported in our previous work,^28^ was selectively borylated to furnish the intermediate **4**. Following the Suzuki reaction with 1,3-dibromo-5-(*tert*-butyl)-2-iodobenzene (**S1**), compound **5** was produced with a 61% yield in two steps from **3**. Then, **5** was coupled with 2-naphthaleneboronic acid (**S2**) to yield the precursor **6**. The final cyclodehydrogenation using DDQ and TfOH afforded the desired ATHN **1** in 43% yield. Furthermore, the synthesis of **2** with extended π-conjugation was also carried out. First, compound **4** was transformed into compound **7** in 68% yield in two steps. The Sonogashira coupling reaction of **7** with 4-*tert*-butylphenylacetylene (**S3**) in the presence of Pd­(PPh_3_)_2_Cl_2_ and CuI gave compound **8** with a yield of 89%. Subsequently, compound **9** was generated in 87% yield via a Diels–Alder cycloaddition between **8** and 2,3,4,5-tetrakis­(*p-tert*-butyl-phenyl)-cyclopentadienone (**S4**). Finally, compound **9** was treated with DDQ/TfOH at 0 °C in CH_2_Cl_2_ to afford the target ATHN **2** in a good yield of 68%. High-resolution matrix-assisted laser desorption/ionization time-of-flight (MALDI-TOF) mass spectrometry initially confirmed the successful formation of **1** and **2**, revealing isotopic distributions consistent with the calculated patterns (Figure S1–2). Subsequent NMR spectroscopic analysis further supported the expected structures, with all ^1^H NMR signals unambiguously assigned using 2D NMR (Figures S3–S14).

ATHNs **1** and **2** were then unambiguously confirmed by single-crystal analysis. Both of them, **1** (CCDC 2473288) and **2** (CCDC 2473289), adopt a highly twisted and rigid helical geometry resulting from the triple helicene backbone (Figure 2). In principle, the presence of asymmetric triple helicene subunits in **1** and **2** could generate up to four possible enantiomeric pairs, respectively. However, only a single pair of enantiomers was observed in the solid state for each compound, in agreement with the NMR data (Figures S3 and S9). The splay angle between the two planes of the terminal rings θ_AF_, θ_GL_ in **1** and θ_AF_, θ_LQ_ in **2** is 35.8°, 41.3° and 33.3°, 53.6°, respectively. These values are significantly smaller than those of the [6]­helicene (59.5°) owing to the lateral extension.^29^ On the other hand, the value of θ_MS_ (60.2°) and θ_GK_ (64.2°) in **1** and **2** is relatively high when compared to pristine [5]­helicene (47.3°)^30^ and [7]­helicene(32.0°)^31^ due to the highly twisted geometry of this motif (Figure 2 and Table S2). The sum of torsional angles for the inner helicene rim (φ) of heptagon-embedded [6]­helicene in **1** (heptagon[6]-**1**, 108.1°), [6]­helicene in **1** ([6]-**1**, 94.2°) and heptagon-embedded [6]­helicene in **2** (heptagon[6]**-2**, 106.0°), are all larger than the pristine [6]­helicene (87.5°).^29^ In contrast, the φ value of pentagon-embedded [6]­helicene in **2** (pentagon[6]-**2**) is 78.9°, smaller than that of [6]­helicene (87.5°) due to the small torsion angle (6.26°) of C50–C56–C105–C106 (highlighted in blue). Additionally, the φ value of pentagon-embedded [7]­helicene in **1** (pentagon[7]-**1**, 110.8°) and [5]­helicene in **2** ([5]-**2**, 72.6°) is comparable to the pristine [7]­helicene (109.3°) and [5]­helicene (67.9°)^30,31^ (Figure 2 and Table S2).

**Figure 2:**
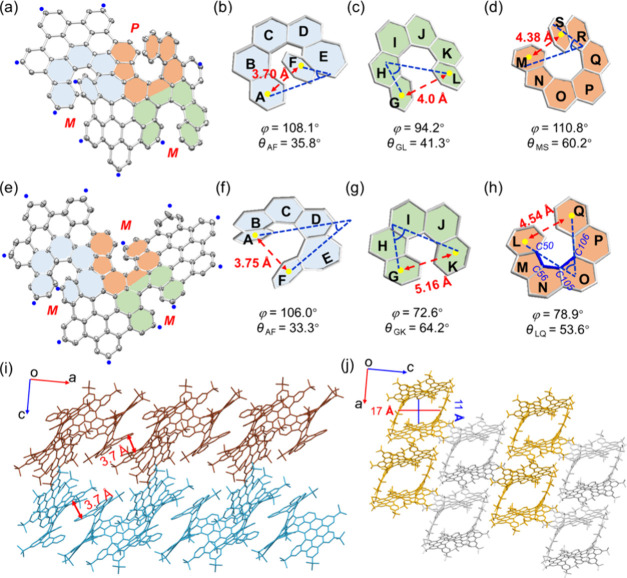
Single-crystal structures of **1** (a) and **2** (e). Thermal ellipsoids are shown at the 30% probability level. The hydrogen atoms and *tert*-butyl groups are omitted for clarity (blue dot, ^*t*^Bu). The three embedded helicene subunits in **1** (b–d) and **2** (f–h), respectively. (i, j) Molecular packing of **1** and **2** in the crystal. **1**: *PPM-* and *MMP-*enantiomers are colored in peacock blue and dark reddish-brown, respectively. **2**: *PPP-* and *MMM*-enantiomers are colored in light gray and golden, respectively.

The centroid–centroid distances (*d*) between the terminal rings of heptagon[6]**-1** (3.70 Å), [6]**-1** (4.0 Å), and heptagon[6]**-2** (3.75 Å) are all smaller than those of pentagon[6]-**2** (4.54 Å) and [6]­helicene (4.48 Å). On the contrary, the *d* values of pentagon[7]**-1** (4.38 Å) and [5]**-2** (5.16 Å) are larger than the corresponding [7]­helicene (3.80 Å) and [5]­helicene (4.99 Å) due to the congested geometry of the helicene backbone (Figure 2). The significant deviation of φ and *d* from those of simple helicene units underscores the high strain and unique geometry imposed by the asymmetric triple helical backbone. In the solid state, compounds **1** and **2** crystallize in the same space group, *P*2_1_/n. The crystal structure of **1** (*PPM* and *MMP*) displays weak intermolecular π–π interactions in the packing pattern, and the enantiomers are alternately assembled in a layer-by-layer manner and arranged themselves in an offset stacking array (Figure 2i). Compound **2** contains a pair of enantiomers (*MMM* and *PPP*) in the unit cell, which form a cavity by two same configurational enantiomers with 17 Å in length and 11 Å in width (Figure 2j).

The optoelectronic properties of **1** and **2** were investigated with UV–vis spectroscopy (Figure 3a). The absorption spectrum of **1** shows a maximum peak (λ_max_) at 660 nm with a weak tail extending to an onset at approximately 712 nm. Time-dependent density functional theory (TD-DFT) calculations assign the 660 nm feature to the S_0_ → S_1_ transition, predominantly arising from HOMO→LUMO (96.6%) excitation with an oscillator strength (*f*) of 0.212 (Figure 3c and Table S3). The optical energy gap of compound **1** is calculated as 1.74 eV from the onset of its UV–vis absorption. In contrast, compound **2** displays significant bathochromic shifts relative to **1**, exhibiting a λ_max_ at 703 nm and an absorption onset at 762 nm. This red-shifted absorption corresponds to the S_0_ → S_1_ transition, dominated by HOMO→LUMO excitation (97.9%) and characterized by a stronger oscillator strength (*f* = 0.528; Figure 3d and Table S4). Consequently, **2** possesses a narrower optical energy gap of 1.63 eV mainly due to its extended π-conjugation. Afterward, the electrochemical properties of **1** and **2** were investigated by CV and differential pulse voltammetry (DPV) in a solution of *n*-Bu_4_NPF_6_ (0.1 M) in CH_2_Cl_2_ (Figure 3b). Compound **1** exhibits four reversible oxidations with half-wave potentials at 0.29, 0.62, 0.99, and 1.29 V, as well as two reversible reduction waves at −1.63 and −1.96 V (vs Fc^+^/Fc), identified by both CV and DPV curves (Figure 3b). Similarly, compound **2** also displays four reversible oxidation waves at 0.15, 0.48, 0.92, and 1.19 V and two reduction waves at −1.60 and −1.88 V, respectively (Figure 3b). Consequently, the HOMO/LUMO levels were estimated to be −4.96/–3.16 eV for **1** and −4.9/–3.3 eV for **2** based on the onset potentials of the first oxidation/reduction waves (Figure 3b). The electrochemical energy gaps were thus calculated to be 1.8 and 1.6 eV for **1** and **2**, respectively, which are in good accordance with their optical energy gaps.

**Figure 3:**
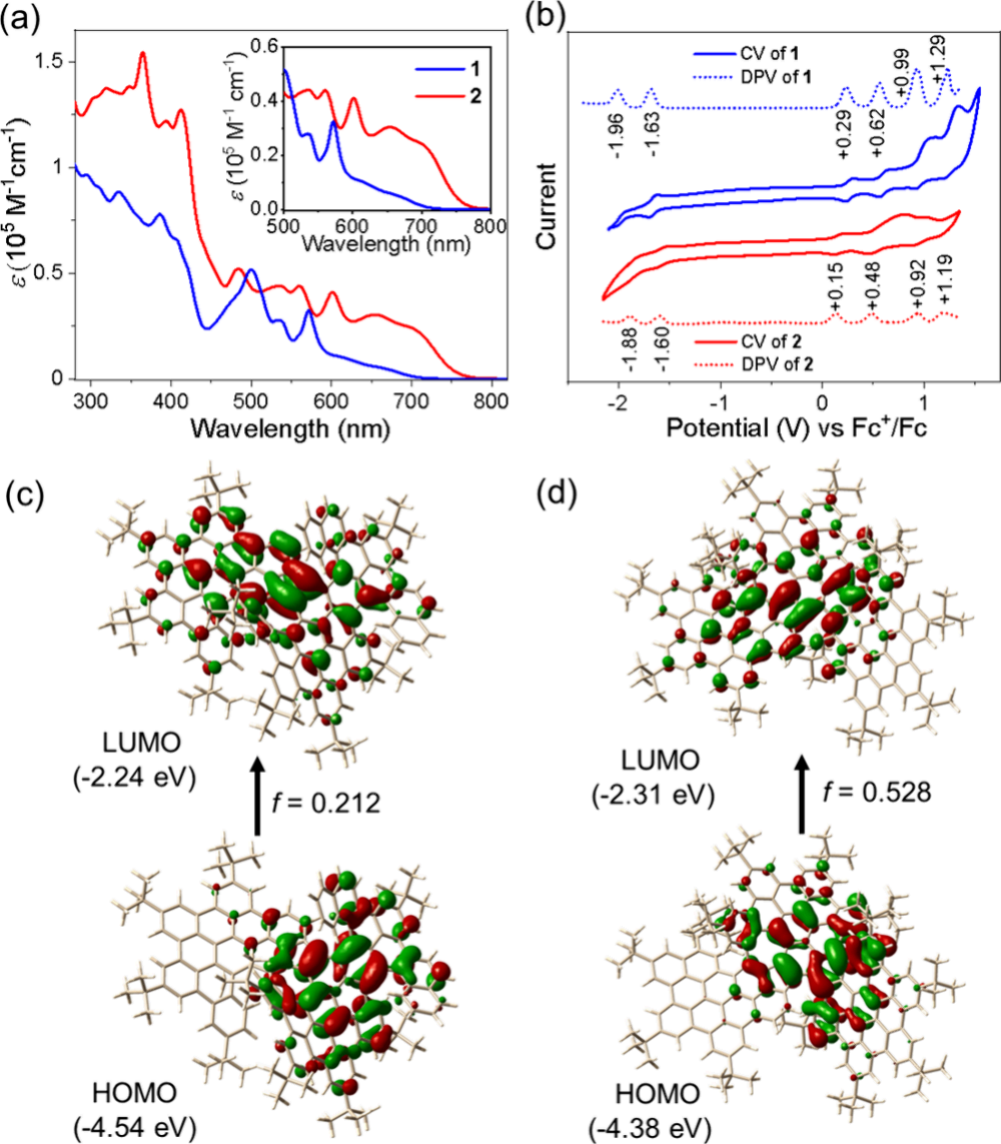
(a) UV–vis absorption spectra of **1** and **2** in CH_2_Cl_2_ (1 × 10^–5^ mol/L). (b) CV and DPV of **1** (blue) and **2** (red) in CH_2_Cl_2_ containing 0.1 M *n*Bu_4_NPF_6_ at a scan rate of 50 mV s^–1^ at room temperature. Optical transitions with molecular orbitals of **1** (c) and **2** (d).

To study the chiroptical properties, HPLC is employed as a critical first step for enantiomer resolution. The racemic mixtures of **1** were resolved using a Chiralpak IA column, employing a gradient eluent of hexane/chloroform (95:5) at 1 mL/min (Figure S15a). Neither racemization nor decomposition was observed after heating the first fraction of **1** in toluene at 100 °C for 4 h, indicating the high configurational stability of compound **1** (Figure S15b). Unfortunately, efforts to resolve the enantiomers of compound **2** via chiral HPLC have not been successful so far, probably due to the low inversion barrier in the embedded [5]­helicene unit. After enantioresolution of compound **1**, their chiroptical properties were studied (Figure 4). The enantiomers revealed perfect mirror images with multiple opposite Cotton effects. Additionally, the simulated ECD spectrum based on TD-DFT calculations is in agreement with the experimental data, confirming the first and second fractions assigned as the *MMP*- and *PPM*-enantiomers, respectively (Figure 4a). The absorption dissymmetry factors (*g*_abs_) range from 2.6 × 10^–4^ to 3.1 × 10^–3^ (Figure 4b). In the S_0_ → S_1_ transition, the electric (μ_e_) and magnetic dipole (μ_m_) moments are oriented with an angle θ = 90.54° between them (Figure 4c and Figure S19). This near-perpendicular alignment results in an exceptionally low |cos θ| value of 0.01 and a high |μ_e_|/|μ_m_| ratio (>400), dramatically reducing the *g*_abs_ (defined as 4 cos θ × |μ_m_|/|μ_e_|) value.

**Figure 4:**
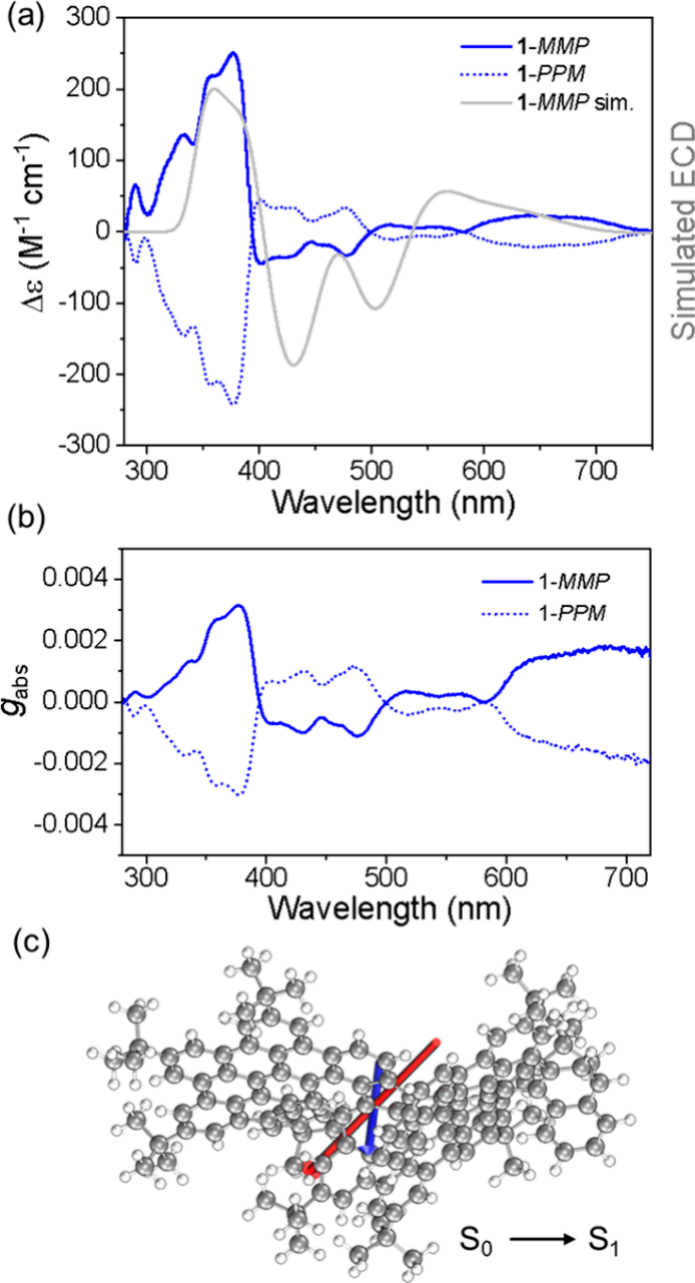
(a) Experimental ECD spectra of the pure enantiomers of **1** in anhydrous CH_2_Cl_2_ in comparison with the simulated ECD spectra of **1**-*MMP*. (b) Absorption dissymmetry factor of enantiomers of **1**. (c) Spatial arrangement of electric (μ_e_) and magnetic (μ_m_) dipole moment vectors for the S_0_ → S_1_ transition.

In summary, we developed a modular synthetic strategy enabling the construction of asymmetric triple helical nonbenzenoid nanographenes **1** and **2** with precise control over helicene length and π-extension. Single-crystal X-ray diffraction unambiguously confirmed their highly twisted helical backbones. ATHN **1** comprises a [6]­helicene, a heptagon-embedded [6]­helicene and a pentagon-embedded [7]­helicene, while ATHN **2** features a [5]­helicene, a heptagon-embedded [6]­helicene and a pentagon-embedded [6]­helicene within its framework. Optical and electrochemical characterizations revealed their tunable and narrow energy gaps (1.74 eV for **1**; 1.63 eV for **2**). Moreover, enantioresolution of **1** was achieved by chiral HPLC, with the enantiomers showing good ECD responses, reaching values of *g*_abs_ up to 3.1 × 10^–3^. This work establishes a versatile platform for designing asymmetric helical NGs and multiple helical systems with controllable helicene length.

## Supplementary Material



## Data Availability

The data underlying this study are available in the published article and its Supporting Information.

## Notes

Experimental procedures and characterization, NMR data, IR data, mass spectrometry data, crystallographic details, HPLC separation, configurational stability study, and computational details. Structural assignments were made with additional information from COSY, HSQC, and HMBC experiments (PDF)
