# Supplementary material for: Asymmetric Triple Helical Nonbenzenoid Nanographenes with Controllable Helicene Lengths
Source: Org Lett. 2025 Sep 5;27(37):10507–12. doi: 10.1021/acs.orglett.5c03372 (PMC12455654; doi:10.1021/acs.orglett.5c03372)
Supplement: Supplementary file 1 [file ol5c03372_si_001.pdf]

Supporting Information

Asymmetric Triple Helical Nonbenzenoid Nanographenes with Controllable Helicene Lengths

Lin Yang[1,+], Yucheng Yin[2,3,+], Wenhui Niu[1,*], Yubin Fu[1], Fupin Liu[4,5], Hartmut Komber[6], Alexey A. Popov[4], Ji Ma[7,*], Xinliang Feng[1,2,*]

[1] *Max Planck Institute of Microstructure Physics, Weinberg 2, 06120 Halle, Germany*

[2] *Centre for Advancing Electronics Dresden (cfaed), Department of Chemistry and Food Chemistry, Technische Universität Dresden, 01062 Dresden, Germany*

[3] *State Key Laboratory of Synergistic Chem-Bio Synthesis, School of Chemistry and Chemical Engineering, Frontiers Science Center for Transformative Molecules, Shanghai Key Laboratory of Electrical Insulation and Thermal Ageing, Shanghai Jiao Tong University, Shanghai 200240, China*

[4] *Leibniz Institute for Solid State and Materials Research, Helmholtzstraße 20, 01069 Dresden, Germany*

[5] *Jiangsu Key Laboratory of New Power Batteries, Jiangsu Collaborative Innovation Center of Biomedical Functional Materials, School of Chemistry and Materials Science, Nanjing Normal University, Nanjing, 210023 China*

[6] *Leibniz-Institut für Polymerforschung Dresden e.V., Hohe Straße 6, 01069 Dresden, Germany*

[7] *College of Materials Science and Opto-Electronic Technology &Center of Materials Science and Optoelectronics Engineering, University of Chinese Academy of Science, 100049 Beijing, P. R. China*

Table of contents

1. General methods and materials S3

2. Detailed synthetic procedure and characterization data of **1** and **2** S5

3. High Resolution (HR) MALDI-TOF mass spectra of **1** and **2** S14

4. NMR spectra of **1** and **2** S15

5. X-ray crystallographic analysis of **1** and **2** S23

6. Determination of enantiopurity **1** by HPLC and configurational stability study S25

7. Photophysical study **1** and **2** S26

8. DFT calculations S27

9. NMR spectra S31

10. References S51

1. General methods and materials

All the reagents were obtained from Sigma Aldrich, TCI, abcr, Alfa Aesar, Strem, fluorochem, and chempur. All these chemicals were used as received without further purification. All reactions dealing with air- or moisture-sensitive compounds were carried out in a dry reaction vessel under argon (Ar) atmosphere by using standard vacuum-line and Schlenk techniques. Anhydrous dichloromethane and tetrahydrofuran were obtained from MBRAUN MB-SPS-5 solvent purification system.

Thin layer chromatography (TLC) was performed on silica-coated aluminium sheets with a fluorescence indicator (TLC silica gel 60 F254, purchased from Merck KGaA).

Column chromatography was performed on silica (SiO_2, particle size 0.063-0.200 mm, purchased from VWR).

NMR spectra were recorded on a Bruker Avance III 500 spectrometer operating at 500.13 MHz for ^{1}H and at 125.77 MHz for ^{13}C at 30°C (unless otherwise stated). The 1D and 2D NMR spectra were recorded using the standard Bruker pulse programs. CD_2Cl_2 (δ(^{1}H) = 5.33 ppm, δ(^{13}C) = 53.7 ppm), and $C_2D_2Cl_4$ (δ(^{1}H) = 5.98 ppm, δ(^{13}C) = 73.7 ppm) were used as solvents and as internal chemical shift reference. Chemical shifts (δ) are reported in ppm. The following abbreviations are used to describe peak patterns as appropriate: s = singlet, d = doublet, t = triplet, q = quartet, and m = multiplet.

The high-resolution matrix-assisted laser desorption/ionization time-of-flight (MALDI-TOF) mass spectrometry was performed on a Bruker Autoflex Speed MALDI TOF MS (Bruker Daltonics, Bremen, Germany) using *trans*-2-[3-(4-*tert*-butylphenyl)-2-methyl-2-propenylidene]malononitrile (DCTB) or dithranol as matrix.

HPLC separation were carried out on an Agilent 1260 series equipped with the following modules: quaternary pump (G7111B 1260 Quat Pump), automatic sample injector (G2258A 1260 DL ALS), column thermostat (G1316A 1260 TCC), DAD detector (G7115A 1260 DADWR) and an automatic sample collector (G1364C 1260 FC-AS).

Electronic circular dichroism (ECD) were recorded in an Olis DSM172 spectrophotometer equipped with a xenon lamp of 150 W.

UV–visible spectra were measured on an Agilent Cary 5000 UV–vis–NIR spectrophotometer by using 10 mm optical-path quartz cell at room temperature.

Cyclic voltammetry (CV) was carried out on a PARSTAT4000 potentiostat (Princeton Applied Research, Ametek, Germany) in a three-electrode cell in degassed dry dichloromethane solution containing 0.1 M of tetra-*n*-butylammonium hexafluorophosphate (n-Bu_4NPF_6) at different scan rates at room temperature. A Pt wire, silver chloride-coated silver wire, and Pt disc electrode were used as the working electrode, the reference electrode, and the counter electrode, respectively. Ferrocene as the reference redox system (−4.8 eV) was used.

2. Detailed synthetic procedure and characterization data of 1 and 2

The precursor **3** was prepared according to the literature method.[1]

Synthesis of the intermediate 4

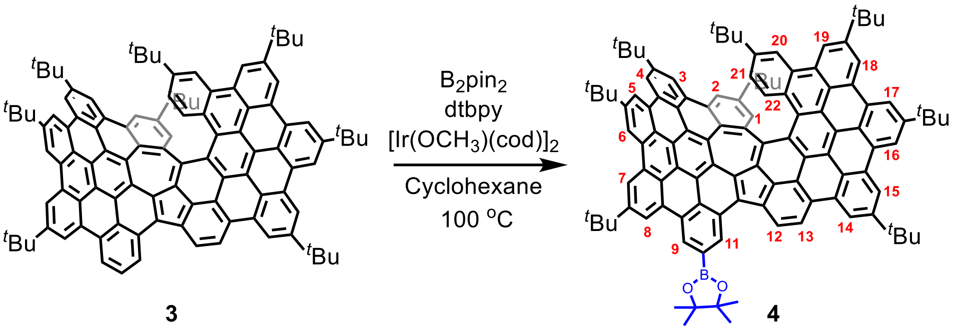

A mixture of compound **3** (400 mg, 0.287 mmol), B_2pin_2 (146 mg, 0.575 mmol), dtbpy (7.7 mg, 0.028 mmol), and $[Ir(OMe)(cod)]_2$ (9.52 mg, 0.014 mmol) in a 20 mL sealed pressure tube was degassed with Ar for 30 min. Then, degassed cyclohexane (10 mL) was added via a syringe. The reaction mixture was stirred at 100 °C in an oil bath for 16 h. After being cooled to room temperature, the solvent was removed under reduced pressure, and the residue was passed through a silica plug (eluent: DCM). The obtained mixture was used directly in the next step because the boronic ester was partially converted to the boronic acid during column chromatography.

oil bath, heating mantle

Synthesis of the intermediate 5

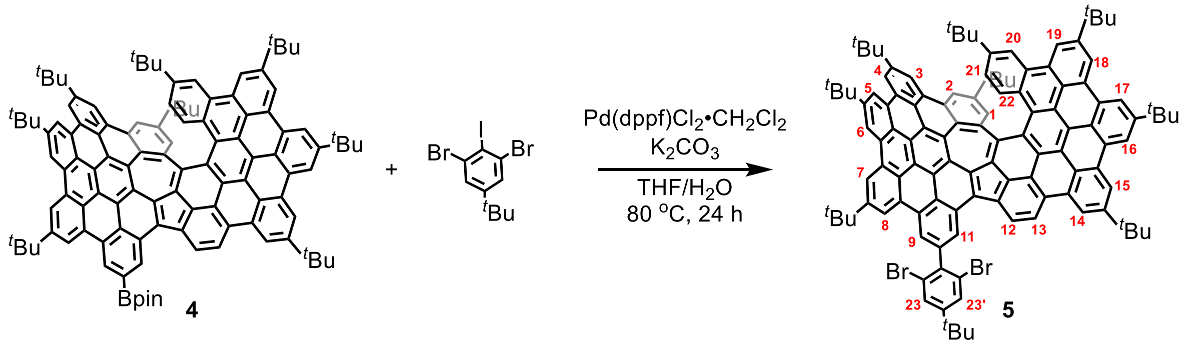

A mixture of compound **4** (300 mg, 0.198 mmol), 1,3-dibromo-5-(*tert*-butyl)-2-iodobenzene (165.2 mg, 0.395 mmol), K_2CO_3 (54.6 mg, 0.395 mmol) and $PdCl_2(dppf)·DCM$ (16.1 mg, 0.02 mmol) was added to a 25 mL Schlenk flask, and degassed with Ar for 30 min. Then, degassed THF/H_2O (5/1, 12 mL) was added via syringe. The reaction mixture was stirred at 80 °C in an oil bath for 24h. After being cooled to room temperature, the solvent was removed under

reduced pressure, and the residue was subjected to column chromatography (silica gel, isohexane/DCM = 10/1 to 5/1) to afford the product **5** (202.7 mg, 61%, over two steps, red solid).

^{1}H NMR (500 MHz, CD_2Cl_2): δ 9.49 (s, 1H; 14), 9.48 (d, 8.4 Hz, 1H; 13), 9.46 (s, 1H; 15), 9.43 (s, 1H; 11), 9.42 (s, 1H; 16), 9.41 (d, 8.4 Hz, 1H; 12), 9.33 (s, 1H; 17), 9.30 (3 x s, 3H; 6, 7, 8), 9.28 (s, 1H; 18), 9.27 (s, 1H; 5), 9.17 (s, 1H; 4), 9.16 (s, 1H; 9), 9.05 (s, 1H; 19), 8.89 (s, 1H; 3), 8.60 (s, 1H; 20), 8.58 (s, 1H; 2), 8.57 (d, 8.4 Hz, 1H; 22), 8.06 (s, 1H; 23), 7.95 (s, 1H; 23'), 7.23 (s, 1H; 1), 6.52 (d, 8.4 Hz, 1H; 21), 1.89 (s, 9H; $^{t}Bu_{14/15}$), 1.88 (s, 9H; $^{t}Bu_{5/6}$), 1.87 (s, 9H; $^{t}Bu_{16/17}$), 1.83 (27H; $^{t}Bu_{3/4}$, $^{t}Bu_{7/8}$, $^{t}Bu_{18/19}$), 1.56 (s, 9H; $^{t}Bu_{23/23'}$), 1.25 (s, 9H; $^{t}Bu_{20/21}$), 0.79 ppm (s, 9H; $^{t}Bu_{1/2}$).

^{13}C NMR (125 MHz, CD_2Cl_2): δ 154.8, 150.4 (2C), 150.2 (2C), 150.1 (4C), 147.9, 142.3, 140.6, 140.0, 139.9, 138.8, 134.9, 134.3, 134.2, 132.8, 132.5 (22), 131.7, 131.6, 131.5, 131.2, 131.1, 131.0, 130.9, 130.8, 130.7, 130.2 (1, C), 130.1 (23), 130.0 (23', C), 129.9, 129.7 (2 C), 129.6, 128.4, 127.5, 127.0, 126.9, 125.0, 124.9, 124.7, 124.5, 124.2 (11), 123.7 – 123.5 (12, 21, xC), 123.4 (2C), 123.2, 122.9, 122.4, 122.1 (9), 121.7 (13), 121.5, 121.0 (4), 120.8, 120.6 (14), 120.2 (15), 120.0 – 119.5 (2, 5 - 8, 16 - 18, xC), 119.2 (19), 119.0 (3, 20), 36.1 (2C), 36.0, 35.9 (3C), 35.3, 35.0, 34.2 (all C_{tBu}), 32.1-31.9 (6 x CH_3; $^{t}Bu_{3/4}$, $^{t}Bu_{5/6}$, $^{t}Bu_{7/8}$, $^{t}Bu_{14/15}$, $^{t}Bu_{16/17}$, $^{t}Bu_{18/19}$), 31.7 (CH_3; $^{t}Bu_{20/21}$), 31.3 (CH_3; $^{t}Bu_{23/23'}$), 30.5 ppm (CH_3; $^{t}Bu_{1/2}$).

HR-MS MALDI-TOF (m/z): calculated for $C_{118}H_{104}Br_2$ $[M]^{+}$, 1678.6499; found, 1678.6524, error = +1.49 ppm.

Synthesis of the intermediate 6

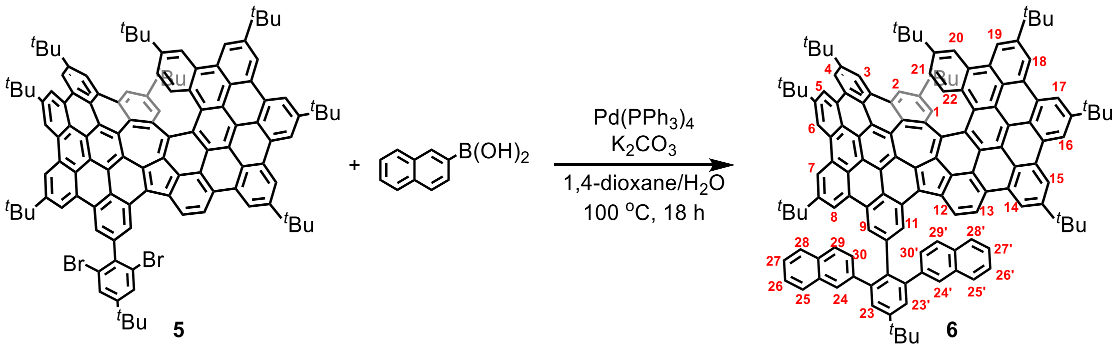

A mixture of compound **5** (60 mg, 35.7 µmol), 2-naphthylbornic (24.5 mg, 142.7 µmol), K_2CO_3 (29.6 mg, 0.214 mmol) and $Pd(PPh_3)_4$ (4.12 mg, 3.57 µmol) was added to a 10 mL Schlenk flask, and degassed with Ar for 30 min. Then, degassed 1,4-dioxane/H_2O (4/1, 2.5 mL) was added via syringe. The reaction mixture was stirred at 100 °C in an oil bath for 18 h. After being

cooled to room temperature, the solvent was removed under reduced pressure, and the residue was subjected to column chromatography (silica gel, isohexane/DCM =10/1 to 5/1) to afford the product **6** (58.3 mg, 92%, red solid).

^{1}H NMR (500 MHz, CD_2Cl_2): δ 9.44 (s, 1H; 15), 9.41 (s, 1H; 16), 9.31 (s, 1H; 17), 9.26 (s, 1H; 18), 9.24 (s, 1H; 14), 9.19 (s, 1H; 5), 9.15 (s, 1H; 6), 9.10 (s, 1H; 4), 9.05 (s, 1H; 11), 9.04 (s, 1H; 7), 9.01 (s, 1H; 19), 8.93 (s, 1H; 9), 8.85 (s, 1H; 3), 8.67 (s, 1H; 30'), 8.56 (s, 1H; 8), 8.55 (s, 1H; 20), 8.52 (s, 1H; 2), 8.46 (d, 8.4 Hz, 1H; 29), 8.42 (d, 8.5 Hz, 1H; 22), 8.33 (d, 8.1 Hz, 1H; 13), 8.15 (s, 1H; 30), 8.06 (s, 1H; 23'), 7.92 (s, 1H; 23), 7.87 (t, 7.6 Hz, 1H; 28), 7.78 (d, 9.1 Hz, 1H; 29'), 7.77 (d, 8.1 Hz, 1H; 12), 7.51 (d, 7.8 Hz, 1H; 26), 7.45 – 7.40 (4H; 27, 24', 25', 26'), 7.33 (d, 8.6 Hz, 1H; 25), 7.27 (t, 7.6 Hz, 1H; 28'), 7.20 (d, 8.6 Hz, 1H; 24), 7.16 (t, 7.2 Hz, 1H; 27'), 7.14 (s, 1H; 1), 6.43 (d, 8.3 Hz, 1H; 21), 1.99 (s, 9H; ^{t}Bu$_{14/15}$), 1.87 (s, 9H; ^{t}Bu$_{16/17}$), 1.83 (s, 9H; ^{t}Bu$_{5/6}$), 1.82 (s, 9H; ^{t}Bu$_{18/19}$), 1.80 (s, 9H; ^{t}Bu$_{3/4}$), 1.68 (s, 9H; ^{t}Bu$_{23/23'}$), 1.55 (s, 9H; ^{t}Bu$_{7/8}$), 1.22 (s, 9H; ^{t}Bu$_{20/21}$), 0.76 ppm (s, 9H; ^{t}Bu$_{1/2}$).

^{13}C NMR (125 MHz, CD_2Cl_2): δ 151.7, 150.4, 150.2 (2C), 150.0 (4C), 149.9 (2C), 147.8, 143.2, 142.3, 141.5 (2C), 141.1, 140.2, 138.8, 137.6, 136.5, 134.7, 134.5, 134.0, 133.7 (2C), 133.0, 132.7, 132.4 (22), 132.1, 131.5, 131.4, 131.2, 131.0, 130.9, 130.7, 130.6 (2C), 130.5, 130.4, 130.3, 130.2, 130.0 (2C), 129.9 (1, C), 129.7 (24' or 25'), 129.4 (30', 2C), 129.2, 129.1, 128.9 (23, 23', 24, 30), 128.7 (29), 128.4, 128.3 (26), 128.1 (29'), 127.6 (26'), 127.3 (11, 24' or 25'), 127.2 (25, C), 126.5, 126.4 (28), 126.2 (27), 126.1 (9, 28'), 125.9 (27'), 125.6, 124.8, 124.6, 124.4, 124.1, 123.7, 123.6, 123.5 (21), 123.4, 123.3 (2C), 123.2 (12, C), 123.1, 122.7, 122.2, 121.2 (13, 2C), 120.9 (4), 120.7, 120.4 (14), 120.0 (15), 119.7 (17), 119.5 (16, 18), 119.4 (6, 7), 119.3 (5), 119.1 (2, 19), 119.0 (8, 20), 118.9 (3), 36.1 (2C), 35.9 (3C), 35.6, 35.3, 34.9, 34.1 (all C_{tBu}), 32.2 (CH_3; ^{t}Bu$_{14/15}$), 32.0-31.9 (4 x CH_3; ^{t}Bu$_{3/4}$, ^{t}Bu$_{5/6}$, ^{t}Bu$_{16/17}$, ^{t}Bu$_{18/19}$), 31.7 (CH_3; ^{t}Bu$_{7/8}$), 31.6 (2 x CH_3; ^{t}Bu$_{20/21}$, ^{t}Bu$_{23/23'}$), 30.5 ppm (CH_3; ^{t}Bu$_{1/2}$).

HR-MS MALDI-TOF (m/z): calculated for $C_{138}H_{118}$ $[M]^+$, 1774.9288; found, 1774.9255, error = -1.86 ppm.

Synthesis of 1

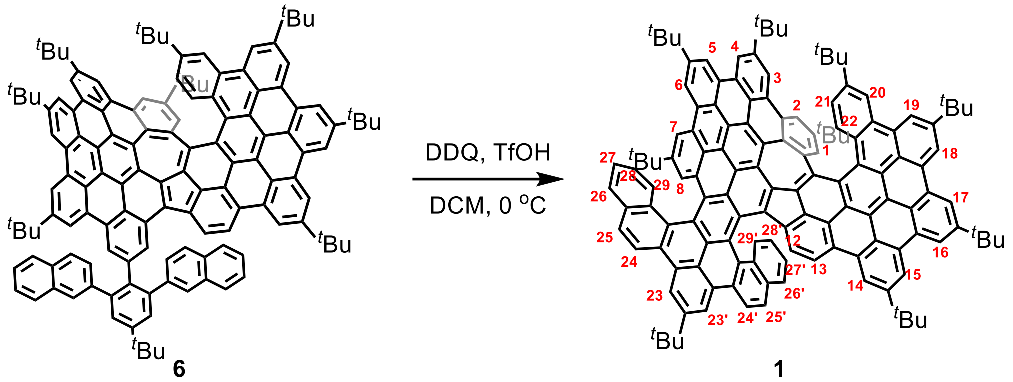

6 1

6 (20 mg, 11.3 μmol) and DDQ (10.22 mg, 0.045 mmol) were dissolved in 10 mL of anhydrous DCM in a 25 mL Schlenk flask equipped with a magnetic stirrer under argon. The solution was cooled down to 0 °C with an ice bath. After stirring for 5 min, 0.5 mL of TfOH was added dropwise through a syringe. The reaction mixture was kept stirred at 0 °C in an ice water bath for 20 min and then quenched with 1 mL Et_3N. The reaction mixture was diluted with 20 mL DCM and the organic layer was washed with water, dried with anhydrous magnesium sulfate and concentrated under reduced pressure. The residue was separated by preparative silica TLC (isohexane/DCM = 5/1). Compound **1** was obtained as a red solid in 43% yield.

^{1}H NMR (500 MHz, CD_2Cl_2): δ 9.37 (s, 1H; 23‘), 9.33 (2H; 15, 16), 9.30 (d, 9.2 Hz, 1H; 24‘), 9.28 (s, 1H; 5), 9.26 (2H; 17, 23), 9.24 (s, 1H; 6), 9.22 (2H; 4, 18), 9.16 (s, 1H; 14), 9.09 (s, 1H; 3), 9.01 (d, 8.9 Hz, 1H; 24), 8.99 (s, 1H; 19), 8.87 (s, 1H; 7), 8.82 (s, 1H; 2), 8.62 (d, 8.6 Hz, 1H; 29‘), 8.57 (d, 8.5 Hz, 1H, 13), 8.50 (3H; 8, 20, 25‘), 8.23 (d, 8.7 Hz, 1H; 25), 8.09 (d, 8.6 Hz, 1H; 29), 8.01 (d, 8.3 Hz, 1H; 22), 7.91 (d, 7.7 Hz, 1H; 26), 7.80 (d, 7.7 Hz, 1H; 26‘), 7.29 (s, 1H; 1), 7.24 (t, 8.0 Hz, 1H; 27), 7.00 (t, 8.0 Hz, 1H; 27‘), 6.93 (t, 8.0 Hz, 1H; 28‘), 6.92 (d, 8.5 Hz, 1H, 12), 6.65 (t, 8.0 Hz, 1H; 28), 6.19 (d, 8.3 Hz, 1H; 21), 1.93 (s, 9H; ^{t}Bu$_{23/23'}$),1.89 (s, 9H; ^{t}Bu$_{5/6}$), 1.87 (s, 9H; ^{t}Bu$_{3/4}$), 1.82 (2 x s, 18H; ^{t}Bu$_{14/15}$, ^{t}Bu$_{16/17}$), 1.80 (s, 9H; ^{t}Bu$_{18/19}$), 1.14 (s, 9H; ^{t}Bu$_{20/21}$), 1.11 (s, 9H; ^{t}Bu$_{7/8}$), 0.96 ppm (s, 9H; ^{t}Bu$_{1/2}$).

^{13}C NMR (125 MHz, CD_2Cl_2): δ 151.2, 150.4, 150.3, 150.2, 150.0, 149.9, 149.8, 148.8, 148.3, 142.7, 139.9, 139.5, 133.5, 133.4, 133.2, 133.0, 132.8, 132.4 (22), 131.3, 131.0 - 130.9 (xC), 130.6, 130.3 – 129.6 (xC), 129.4 – 129.0 (1, 25, 25‘, xC), 128.8, 128.5, 128.3 (29), 128.2, 128.1 (26), 127.8 (26‘), 127.7 (29‘), 126.6 (8, C), 126.0 (27‘), 125.8 (27, 28), 125.7 (28‘), 125.1, 124.9, 124.8, 124.5, 124.2 (2C), 124.0, 123.8 (12), 123.6, 123.5, 123.3, 123.2 (21), 123.1, 123.0, 122.5, 122.4 (24), 122.2, 121.6 (24‘), 120.8 (4 or 18), 120.7 (2C), 120.5, 120.4 (13), 120.1 (14), 120.0 (15), 119.6 (5, 17, 23), 119.5 (4 or 18, 16, 23’), 119.1 (6, C), 119.0 (2, 19), 118.7 (3),

118.5 (7), 118.4 (20), 36.3, 36.0 (4C), 35.9, 34.9, 34.8, 34.5 (all C_{tBu}), 32.2 (CH_3; ^{t}Bu$_{23/23'}$), 32.0 (4 x CH_3; ^{t}Bu$_{3/4}$, ^{t}Bu$_{5/6}$, ^{t}Bu$_{14/15}$, ^{t}Bu$_{16/17}$), 31.9 (CH_3; ^{t}Bu$_{18/19}$), 31.4 (CH_3; ^{t}Bu$_{20/21}$), 31.3 (CH_3; ^{t}Bu$_{7/8}$), 30.8 ppm (CH_3; ^{t}Bu$_{1/2}$).

HR-MS MALDI-TOF (m/z): calculated for $C_{138}H_{114}$ $[M]^+$, 1770.8915; found, 1774. 8964, error = +2.77 ppm.

Synthesis of the intermediate 7

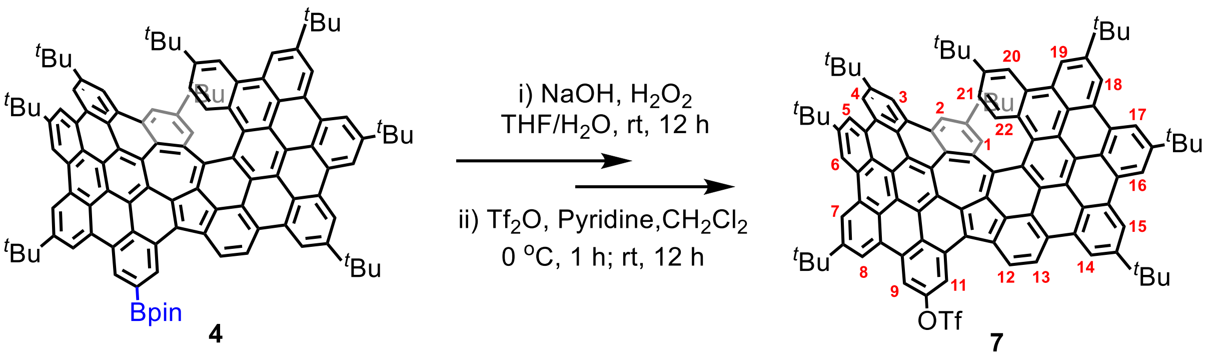

A mixture of compound **4** (436.0 mg, 0.287 mmol) and NaOH (34.5 mg, 0.862 mmol) were dissolved in THF/H_2O (4:1, 10 mL). To this mixture, an aqueous solution of H_2O_2 (30%, 29.31mg, 0.862 mmol) was added slowly. After stirring for 12 h, the solution was acidified to pH 1-2 by using 1M HCl. The reaction mixture was extracted with DCM (3 times). The organic fractions were dried over $MgSO_4$ and the solvent was removed by slow evaporation. The obtained mixture was used for the next step directly. Then, the mixture and anhydrous dichloromethane were added to a 20 mL Schlenk flask under Argon atmosphere. Pyridine and trifluoromethanesulfonic anhydride were added at 0 °C in an ice water bath for 1 h. The mixture was then warmed to room temperature and stirred for 12 h. The reaction was quenched with H_2O and extracted with CH_2Cl_2. The organic layers dried with anhydrous $MgSO_4$ and concentrated under reduced pressure. The residue was purified by column chromatography (silica gel, isohexane/DCM = 10/1 to 5/1) to afford **7** (298.08 mg, 67.5%) as a red solid.

^{1}H NMR (500 MHz, $C_2D_2Cl_4$, 60°C): δ 9.48 (d, 8.0 Hz, 1H; 13),9.44 (s, 1H; 14), 9.42 (s, 1H; 15), 9.39 (s, 1H; 16), 9.29 (3H; 7, 11, 17), 9.28 (br, 1H; 12), 9.25 (3H; 5, 6, 18), 9.14 (2 x s, 2H; 4, 8), 9.02 (s, 1H; 19), 8.96 (s, 1H; 9), 8.86 (s, 1H; 3), 8.57 (s, 1H; 20), 8.55 (s, 1H; 2), 8.48 (d, 8.4 Hz, 1H; 22), 7.27 (s, 1H; 1), 6.53 (d, 8.4 Hz, 1H; 21), 1.88 and 1.87 (27H; ^{t}Bu$_{5/6}$, ^{t}Bu$_{14/15}$, ^{t}Bu$_{16/17}$), 1.84 (s, 9H; ^{t}Bu$_{7/8}$), 1.83 (s, 9H; ^{t}Bu$_{18/19}$), 1.82 (s, 9H; ^{t}Bu$_{3/4}$), 1.25 (s, 9H; ^{t}Bu$_{20/21}$), 0.79 ppm (s, 9H; ^{t}Bu$_{1/2}$).

^{13}C NMR (125 MHz, $C_2D_2Cl_4$, 60°C): δ 151.0, 150.6, 14.2, 148.5, 143.4, 139.3, 138.9, 136.2, 134.6, 133.4, 133.0 (22), 132.1, 131.9, 131.4, 131.3, 130.9 (1), 130.8, 130.2, 129.7, 128.8, 128.0, 127.6, 126.6, 125.2, 124.9, 124.2 (21), 124.0, 123.9, 123.7, 123.5 (12), 123.1, 122.5 (13), 122.1, 121.9, 121.5 (7), 121.4 (4), 121.1 (14), 120.7 (15), 120.5 (8), 120.3 (2), 120.2 (17), 120.0 (5, 6, 16, 18), 119.6 (3, 19), 119.6 (20), 113.9 (11), 112.4 (9), 32.8 – 32.6 (6 x CH_3; ^{t}Bu$_{3/4}$, ^{t}Bu$_{5/6}$, ^{t}Bu$_{7/8}$, ^{t}Bu$_{14/15}$, ^{t}Bu$_{16/17}$, ^{t}Bu$_{18/19}$), 32.4 (CH_3; ^{t}Bu$_{20/21}$), 31.2 ppm (CH_3; ^{t}Bu$_{1/2}$).

^{19}F NMR (470 MHz, $C_2D_2Cl_4$, 60°C): -72.9 ppm (CF_3).

HR-MS MALDI-TOF (m/z): calculated for $C_{109}H_{93}F_3O_3S$ $[M]^+$, 1538.6792; found, 1538.6759, error = -2.14 ppm.

Synthesis of the intermediate 8

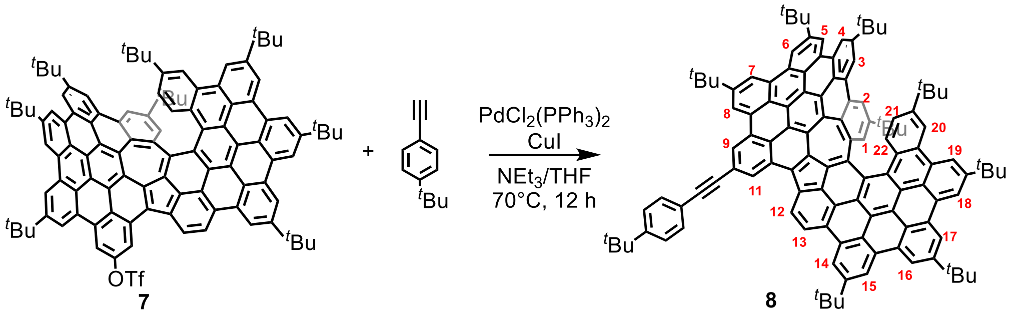

A mixture of compound **7** (160.0 mg, 0.104 mmol), $PdCl_2(PPh_3)_2$ (7.29 mg, 10.4 μmol) and CuI (3.96 mg, 20.8 μmol) in a 20 mL Schlenk flask was degassed with Ar for 30 min. Then, degassed Et_3N/THF (7/7 mL) and 4-*tert*-butylphenylacetylene (0.18 mL, 1.04 mmol) were added via syringe. The reaction mixture was stirred at 70 °C in an oil bath for 12 h. After being cooled to room temperature, the solvent was removed under reduced pressure, and the residue was subjected to column chromatography (silica gel, isohexane/DCM = 10/1 to 5/1) to afford the product **8** (143.1 mg, 89%, red solid).

NMR (500 MHz, $C_2D_2Cl_4$): δ 9.62 (s, 1H; 11), 9.51 (AB spin system, 2H; 12, 13), 9.47 (s, 1H; 14), 9.44 (s, 1H; 15), 9.42 (s, 1H; 16), 9.35 (s, 1H; 9), 9.32 (s, 1H; 17), 9.31 (s, 1H; 8), 9.28-9.25 (4H; 5, 6, 7, 18), 9.14 (s, 1H; 4), 9.05 (s, 1H; 19), 8.86 (s, 1H; 3), 8.60 (s, 1H; 20), 8.57 (d, 8.3 Hz, 1H; 22), 8.55 (s, 1H; 2), 7.85 (d, 8.0 Hz, 2H; 25), 7.58 (d, 8.0 Hz, 2H; 26), 7.25 (s, 1H; 1), 6.57 (d, 8.3 Hz, 1H; 21), 1.91 and 1.89 (27H; ^{t}Bu$_{5/6}$, ^{t}Bu$_{14/15}$, ^{t}Bu$_{16/17}$), 1.87 (s, 9H; ^{t}Bu$_{7/8}$), 1.85 (s, 9H; ^{t}Bu$_{18/19}$), 1.84 (s, 9H; ^{t}Bu$_{3/4}$), 1.46 (s, 9H; ^{t}Bu$_{26}$), 1.29 (s, 9H; ^{t}Bu$_{20/21}$), 0.81 ppm (s, 9H; ^{t}Bu$_{1/2}$).

^{13}C NMR (125 MHz, $C_2D_2Cl_4$): 153.0, 151.0, 150.9, 150.7 (2C), 150.6, 150.5 (2C), 148.4, 143.0, 139.8, 138.8, 135.5, 134.2, 134.0, 133.2, 133.0 (22), 132.6 (25), 132.4, 132.0 (2C), 131.7, 131.5 (2C), 131.2 (2C), 130.9 (1, 2C), 130.6, 130.5, 130.4 (3C), 130.2, 130.0, 129.9, 128.8, 128.0, 127.4 (2C), 126.5 (26), 126.1 (11), 125.4, 125.2, 125.1, 125.0, 124.2 (12 or 13), 124.1 (21), 123.8 (9), 123.7, 123.5, 122.9, 122.8, 122.3 (12 or 13), 122.2, 122.1, 121.5 (4), 121.3, 121.0 (14), 120.9, 120.7 – 119.8 (5 - 8, 15 -18, xC), 119.7 (19), 119.5 (3, 20), 91.8 (24), 90.9 (23), 36.6 (3C), 36.4 (3C), 35.7, 35.5, 34.7 (all C_{tBu}), 32.8 – 32.5 (6 x CH_3; ^{t}Bu$_{3/4}$, ^{t}Bu$_{5/6}$, ^{t}Bu$_{7/8}$, ^{t}Bu$_{14/15}$, ^{t}Bu$_{16/17}$, ^{t}Bu$_{18/19}$), 32.5 (CH_3; ^{t}Bu$_{20/21}$), 32.1 (CH_3; ^{t}Bu$_{26}$), 31.3 ppm (CH_3; ^{t}Bu$_{1/2}$).

HR-MS MALDI-TOF (m/z): calculated for $C_{120}H_{106}$ $[M]^+$, 1546.8289; found, 1546.8233, error = -3.62 ppm.

Synthesis of the intermediate 9

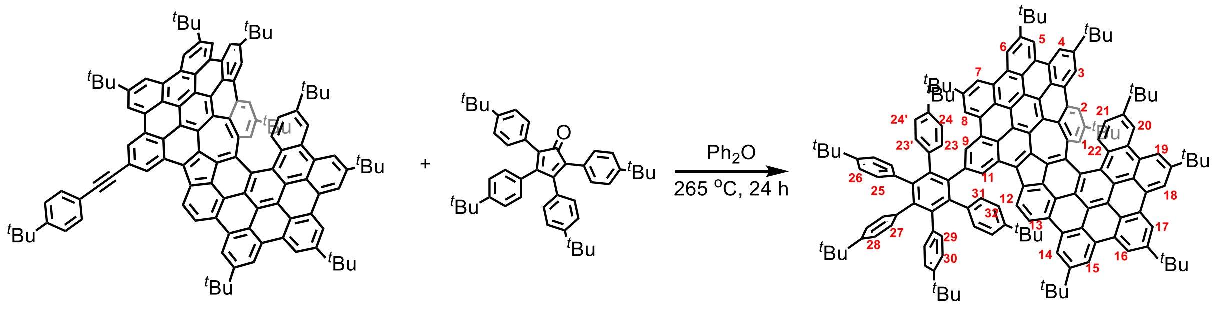

A degassed solution of **8** (50.0 mg, 32.3 µmol) and 2,3,4,5-tetrakis(*p-tert*-butyl-phenyl)-cyclopentadienone (49.2 mg, 80.7 µmol) in 0.5 mL diphenyl ether was refluxed for 24 h using a heating mantle. After cooling down to room temperature, the mixture was subjected to column chromatography (silica gel, isohexane to remove the diphenyl ether, then, isohexane/DCM = 6/1) to afford the product **9** (59.8 mg, 87%, red solid).

^{1}H NMR (500 MHz, CD_2Cl_2): δ 9.47 (s, 1H; 14), 9.44 (s, 1H; 15), 9.41 (s, 1H; 16), 9.34 (d, 8.2 Hz, 1H; 13), 9.31 (s, 1H; 17), 9.26 (s, 1H; 18), 9.20 (s, 2H; 5, 6), 9.14 (s, 1H; 7), 9.11 (s, H; 4), 9.02 (s, 1H; 19), 8.93 (s, 1H; 11), 8.84 (s, 1H; 8), 8.83 (s, 1H; 3), 8.79 (d, 8.2 Hz, 1H; 12), 8.74 (s, 1H; 9), 8.57 (s, 1H; 20), 8.53 (d, 8.4 Hz, 1H; 22), 8.50 (s, 1H; 2), 7.46 (dd, 1H; 31), 7.29 (dd, 1H; 32), 7.22 (dd, 1H; 31'), 7.14 (s, 1H; 1), 7.13 (dd, 1H; 23'), 7.12 – 6.90 (12 H; 25 – 30, 25' – 30'), 6.88 (dd, 1H; 32'), 6.85 (dd, 1H; 24'), 6.80 (dd, 1H; 23), 6.47 (d, 8.4 Hz, 1H; 21), 6.46 (dd, 1H; 24), 1.91 (s, 9H; ^{t}Bu$_{14/15}$), 1.86 (s, 9H; ^{t}Bu$_{16/17}$), 1.85 (s, 9H; ^{t}Bu$_{5/6}$), 1.82 (s, 9H; ^{t}Bu$_{18/19}$), 1.81 (s, 9H; ^{t}Bu$_{7/8}$), 1.80 (s, 9H; ^{t}Bu$_{3/4}$), 1.25 (s, 9H; ^{t}Bu$_{26\text{ or }28\text{ or }30}$), 1.23 (s, 9H; ^{t}Bu$_{20/21}$),

1.21 (s, 9H; ^{t}Bu$_{26\ or\ 28\ or\ 30}$), 1.15 (s, 9H; ^{t}Bu$_{26\ or\ 28\ or\ 30}$), 0.77 (s, 9H; ^{t}Bu$_{32}$), 0.76 (s, 9H; ^{t}Bu$_{1/2}$), 0.46 ppm (s, 9H; ^{t}Bu$_{24}$).

^{13}C NMR (125 MHz, CD_2Cl_2): δ 150.4, 150.1, 150.0 (3C), 149.9 (2C), 148.7, 148.4 (4C), 147.7, 141.8, 141.7, 141.6, 141.5, 141.2, 140.8, 140.7, 140.2, 139.9, 138.7 (2C), 138.5 (4C), 134.8, 134.2, 134.0, 132.7, 132.4 (22, 31'), 132.0 (31), 131.8 – 131.4 (23', 25, 25', 27, 27', 29, 29', 4C), 131.2 (23, C), 131.1, 130.9, 130.8, 130.6 (3C), 130.4, 130.2, 130.0 (1, 3C), 129.9, 129.5, 129.3, 129.2, 129.0, 128.4, 127.3, 127.0 (11), 126.5, 125.9 (9), 125.0, 124.8, 124.7, 124.6 (32'), 124.5 (12), 124.3 (32), 124.0 (24, C), 123.8 - 123.5 (21, 24', 26, 26', 28, 28', 30, 30', 3C), 123.4 (2C), 123.3, 123.1, 122.7, 122.2, 121.4 (13, C), 121.1, 120.9 (4), 120.8, 120.4 (14), 120.0 (15), 119.7 (17), 119.5 (16, 18), 119.4 - 118.9 (2, 3, 5 - 8, 19, 20), 36.1 (2C), 35.9 (xC), 34.9, 34.4 (3C), 34.1 (2C), 33.9 (all C_{tBu}), 32.1 – 31.9 (6 x CH_3; ^{t}Bu$_{3/4}$, ^{t}Bu$_{5/6}$, ^{t}Bu$_{7/8}$, ^{t}Bu$_{14/15}$, ^{t}Bu$_{16/17}$, ^{t}Bu$_{18/19}$), 31.7 (CH_3; ^{t}Bu$_{20/21}$), 31.3 (3 x CH_3; ^{t}Bu$_{26}$, ^{t}Bu$_{28}$, ^{t}Bu$_{30}$), 30.8 (^{t}Bu$_{32}$), 30.5 ppm (2 x CH_3; ^{t}Bu$_{1/2}$, ^{t}Bu$_{24}$).

HR-MS MALDI-TOF (m/z): calculated for $C_{164}H_{158}$ $[M]^+$, 2127.2358; found, 2127.2301, error = -2.68 ppm.

Synthesis of 2

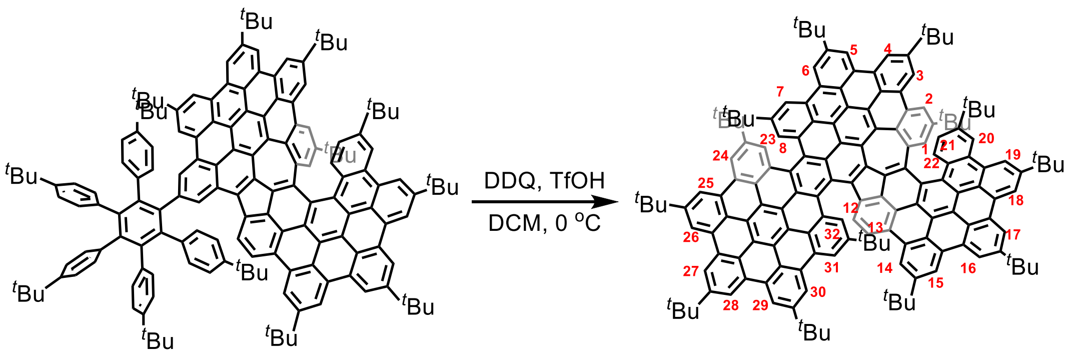

9 **2**

9 (20 mg, 9.4 μmol) and DDQ (25.6 mg, 0.113 mmol) were dissolved in 10 mL of anhydrous DCM in a 25 mL Schlenk flask equipped with a magnetic stirrer under argon. The solution was cooled down to 0 °C with an ice water bath. After stirring for 5 min, 0.5 mL of TfOH was added dropwise through a syringe. The reaction mixture was kept stirred at 0 °C for 20 min and then quenched with 1 mL Et_3N. The reaction mixture was diluted with 20 mL DCM and the organic layer was washed with water, dried with anhydrous magnesium sulfate and concentrated under reduced pressure. The residue was separated by preparative silica TLC (isohexane/DCM = 5/1). Compound **2** was obtained as a red solid in 68% yield.

^{1}H NMR (500 MHz, CD_2Cl_2): δ 9.75 (s, 1H; 23), 9.63 (s, 1H; 30), 9.62 (s, 1H; 8), 9.57 (s, 1H; 29), 9.55 (s, 1H; 28), 9.52 (s, 1H; 27), 9.49 (s, 1H; 26), 9.44 (s, 1H; 25), 9.40 (3H; 15, 16, 31), 9.34 (s, 1H; 17), 9.33 (s, 1H; 5), 9.32 (s, 1H; 4), 9.31 (s, 1H; 18), 9.29 (s, 1H; 6), 9.26 (2H; 14, 24), 9.18 (d, 8.4 Hz, 1H; 22), 9.16 (s, 1H; 32), 9.14 (s, 1H; 7), 9.11 (s, 1H; 19), 8.83 (d, 8.4 Hz, 1H; 13), 8.80 (s, 1H; 3), 8.72 (s, 1H; 20), 8.36 (s, 1H; 2), 7.69 (d, 8.4 Hz, 1H; 12), 7.20 (s, 1H; 1), 6.93 (d, 8.4 Hz, 1H; 21), 2.00 (s, 9H; $^{t}Bu_{29/30}$), 1.95 (s, 9H; $^{t}Bu_{27/28}$), 1.91 (s, 9H; $^{t}Bu_{25/26}$), 1.89 (18H; $^{t}Bu_{3/4}$, $^{t}Bu_{5/6}$), 1.87 (s, 9H; $^{t}Bu_{16/17}$), 1.86 (s, 9H; $^{t}Bu_{18/19}$), 1.81 (s, 9H; $^{t}Bu_{14/15}$), 1.43 (s, 9H; $^{t}Bu_{20/21}$), 1.39 (s, 9H; $^{t}Bu_{23/24}$), 1.38 (s, 9H; $^{t}Bu_{7/8}$), 1.05 (s, 9H; $^{t}Bu_{31/32}$), 0.68 ppm (s, 9H; $^{t}Bu_{1/2}$).

^{13}C NMR (125 MHz, CD_2Cl_2): δ 150.1 (2C), 150.1 – 149.9 (xC), 149.5, 149.2, 148.7, 147.9, 142.6, 137.2, 137.0, 136.4, 136.1, 134.7, 133.6, 133.2, 133.4, 132.1 (22), 131.5 (2C), 131.3 (1), 131.2 -130.2 (xC), 129.8 (1), 129.2, 128.8, 128.6, 128.2 (2C), 128.1, 127.9, 127.0 (23), 126.9 (8), 126.8 (12), 126.2, 125.8 (2C), 124.7, 124.4 (2C), 124.3, 124.0 (21), 123.9 (2C), 123.8, 123.7, 123.6 (2C), 123.5, 123.3 (2C), 123.1, 123.0 (2C), 122.8 (2C), 122.0, 121.8, 121.7 (2C), 121.5 (2), 121.2 (2C), 121.1, 120.5 (4, C), 120.0 (13, 14), 119.9 – 119.3 (5 - 7, 15 – 18, 24 - 31), 119.2, 36.2 – 31.9 (xC), 35.6, 35.5, 35.3, 35.1, 34.0 (all C_{tBu}), 32.2 (CH_3; $^{t}Bu_{29/30}$), 32.1 (2 x CH_3; $^{t}Bu_{27/28}$, $^{t}Bu_{25/26}$), 32.*0* (6 x CH_3; $^{t}Bu_{3/4}$, $^{t}Bu_{5/6}$, $^{t}Bu_{14/15}$, $^{t}Bu_{16/17}$, $^{t}Bu_{18/19}$, $^{t}Bu_{20/21}$), 31.7 (CH_3; $^{t}Bu_{23/24}$), 31.6 (CH_3; $^{t}Bu_{7/8}$), 31.0 (CH_3; $^{t}Bu_{31/32}$), 30.3 ppm (CH_3; $^{t}Bu_{1/2}$).

HR-MS MALDI-TOF (m/z): calculated for $C_{164}H_{146}$ $[M]^+$, 2115.1419; found, 2115.1424, error = +0.24 ppm.

3. High Resolution (HR) MALDI-TOF mass spectra of 1 and 2

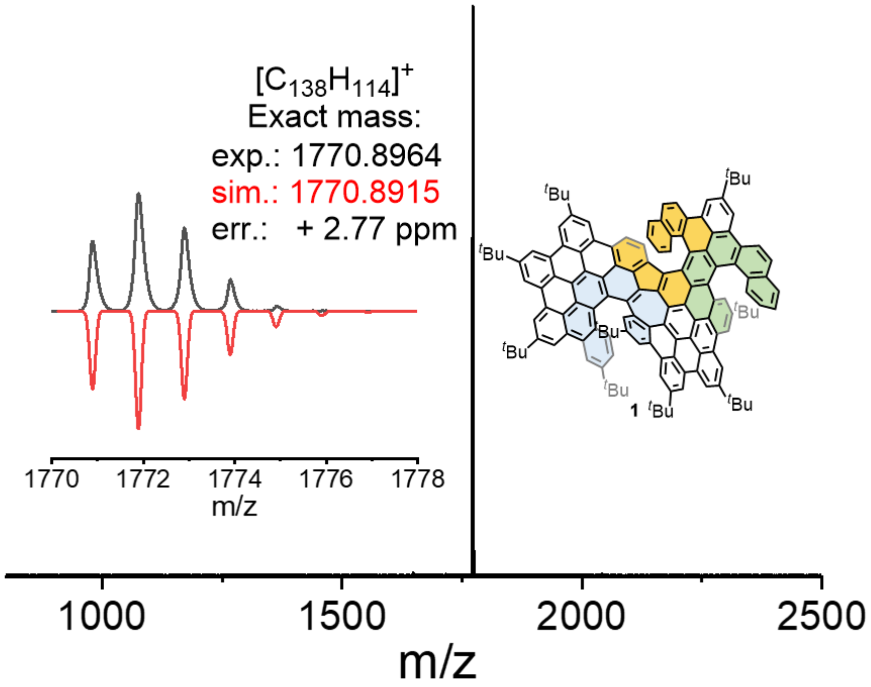

Figure S1. HR MALDI-TOF mass spectrum of **1**.

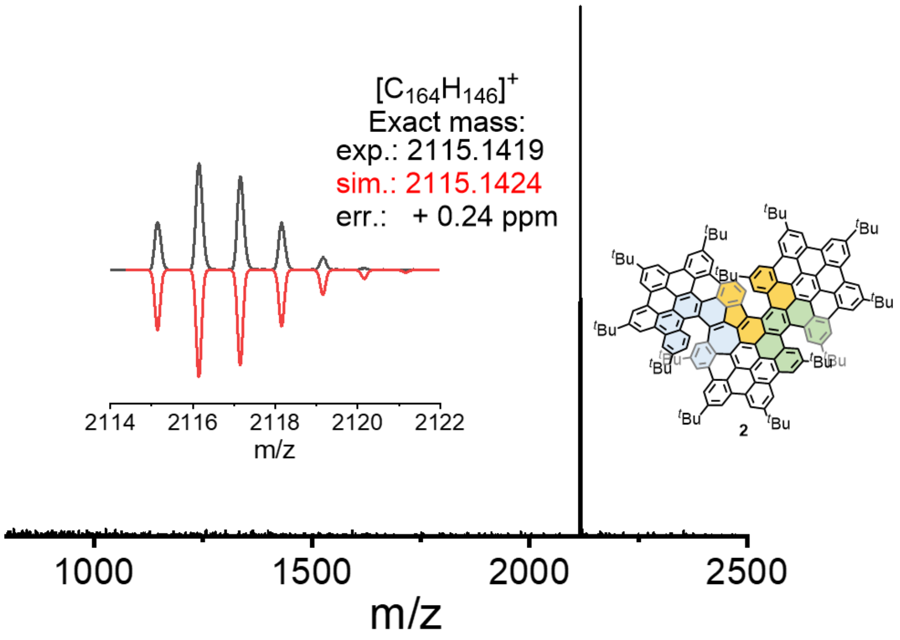

Figure S2. HR MALDI-TOF mass spectrum of **2**.

4 NMR spectra of 1 and 2

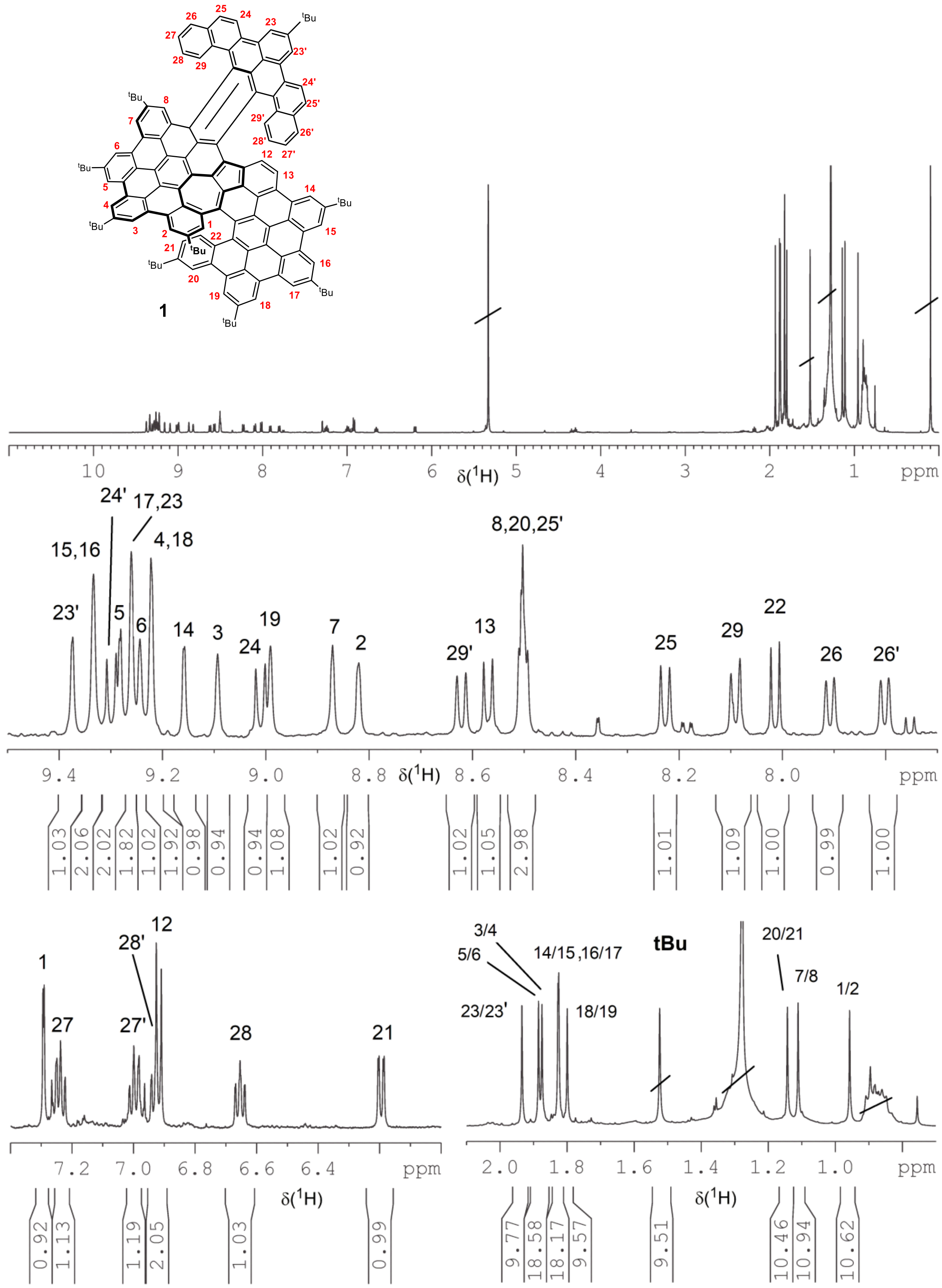

Figure S3. ^{1}H NMR spectrum of **1** and expanded regions (500 MHz, CD_2Cl_2).

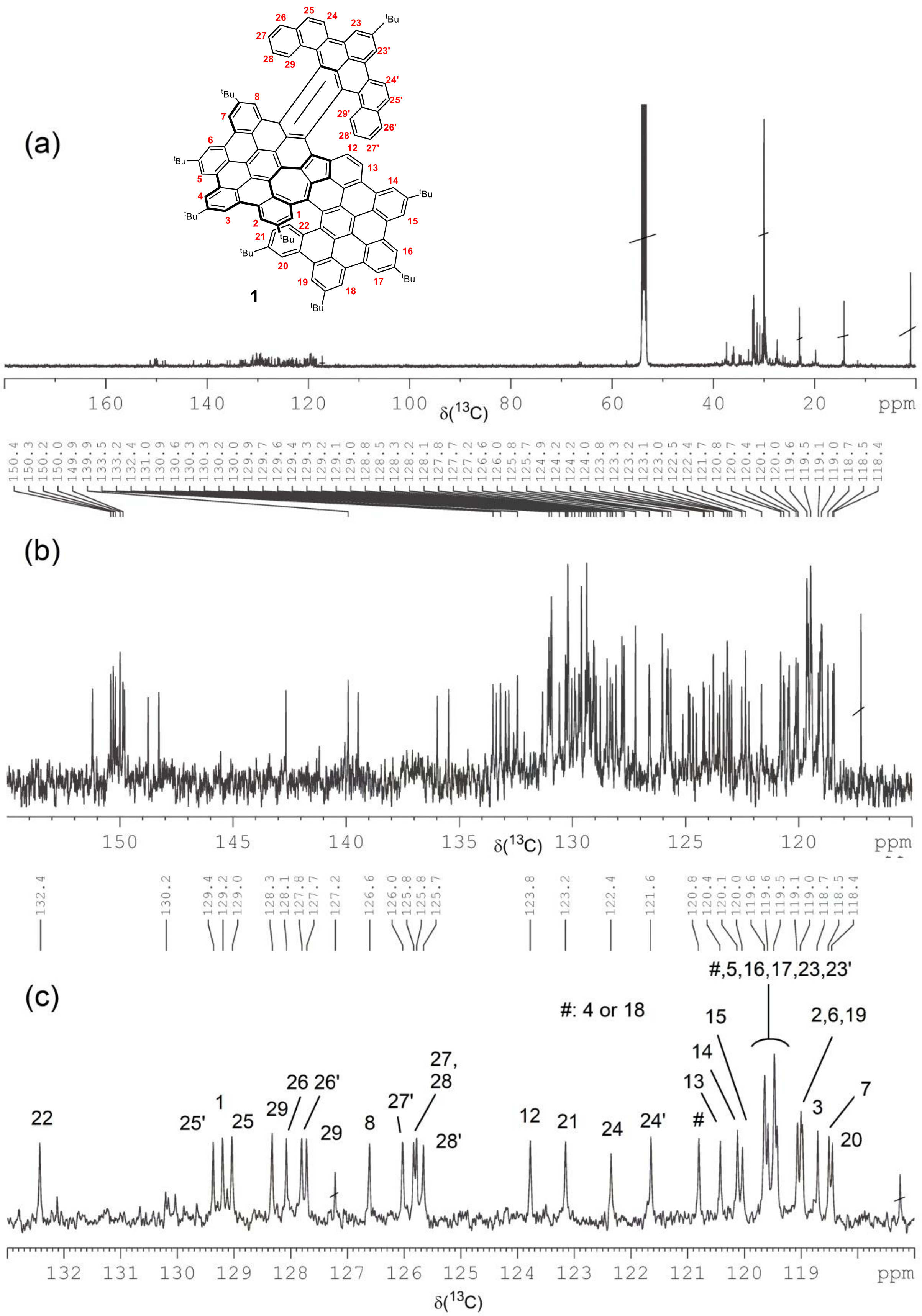

Figure S4. ^{13}C NMR spectrum of **1** (a), expanded region (b) and region of the DEPT135 spectrum (c) in CD_2Cl_2 (125 MHz).

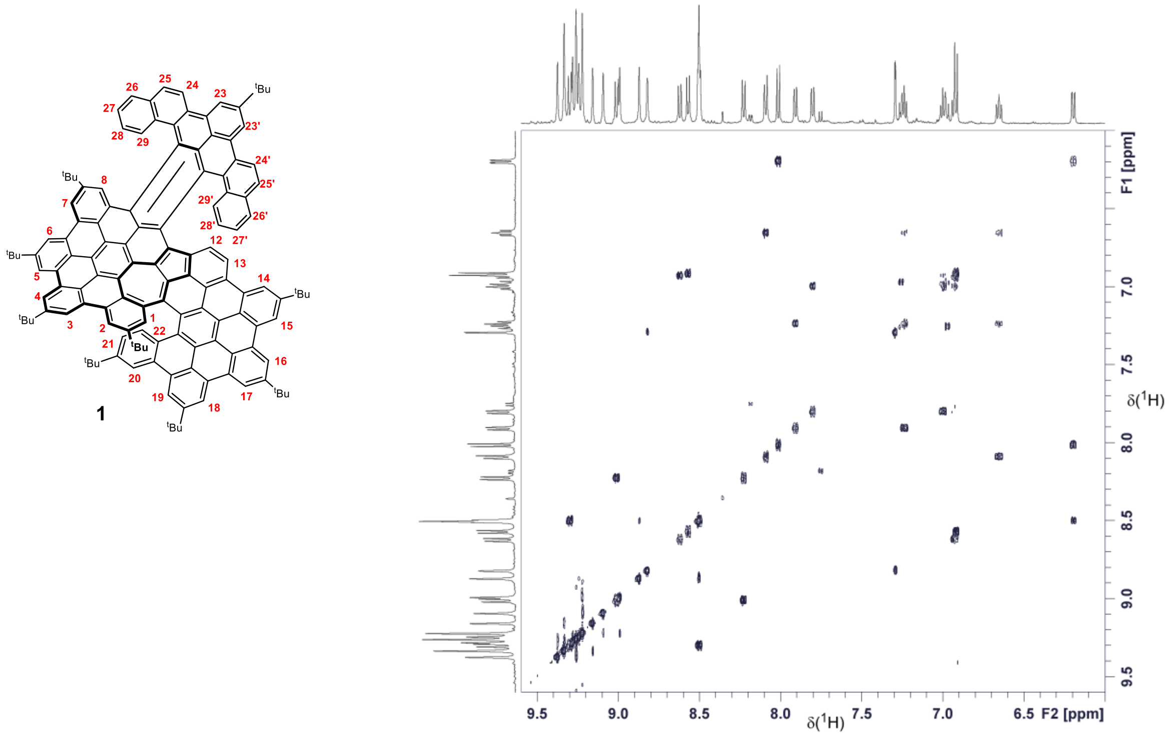

Figure S5. COSY spectrum (region of aromatic protons) of **1** (500 MHz, CD_2Cl_2).

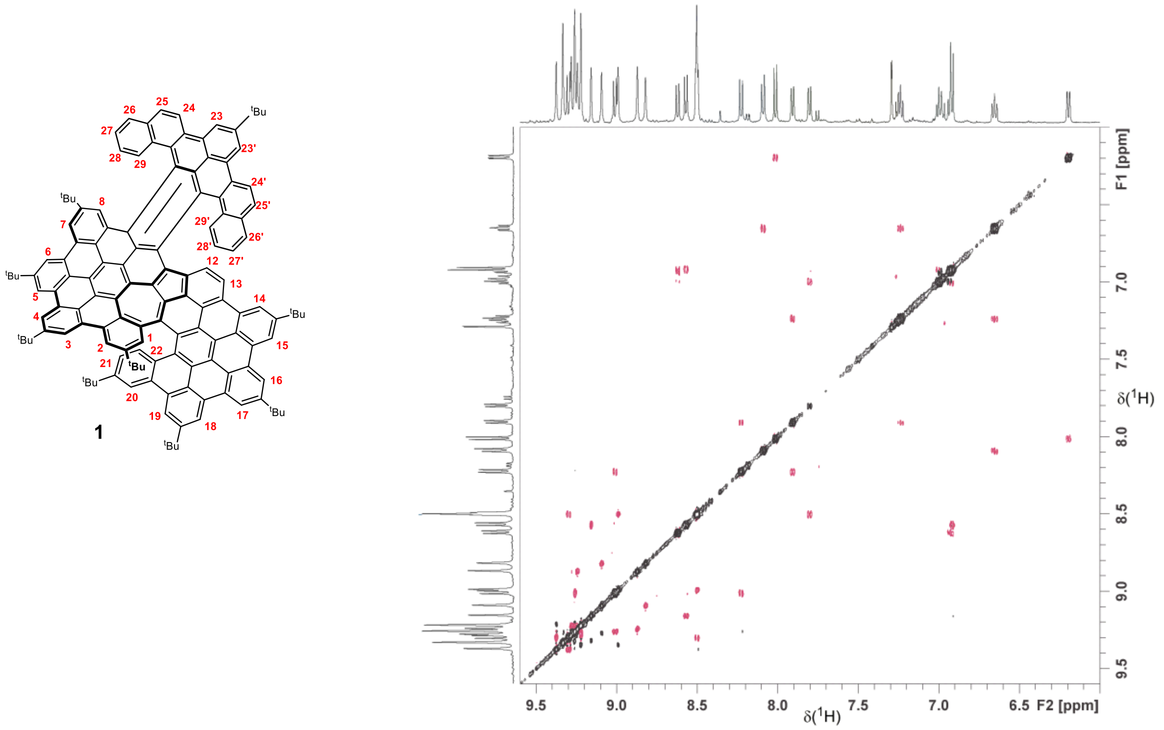

Figure S6. ROESY spectrum (region of aromatic protons) of **1** (500 MHz, CD_2Cl_2).

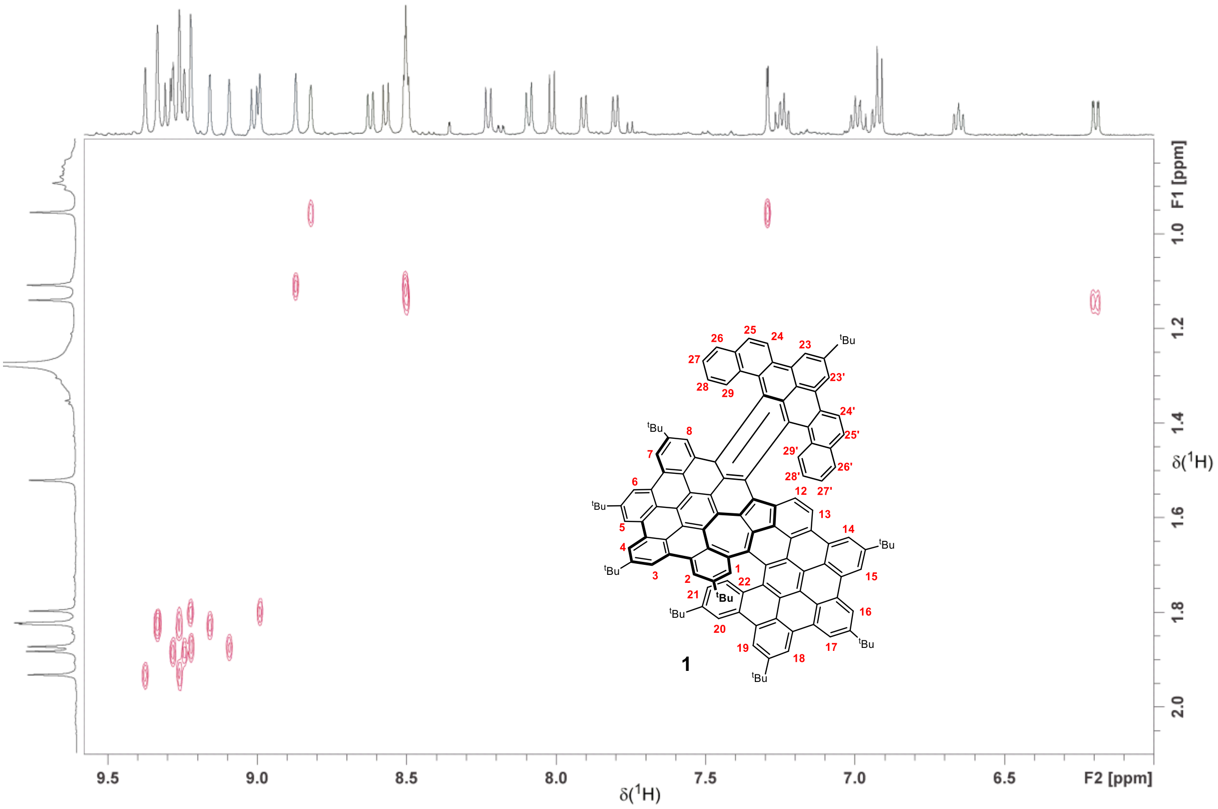

Figure S7. ROESY spectrum (correlations between CH_3 groups and aromatic protons) of **1** in CD_2Cl_2 (500 MHz).

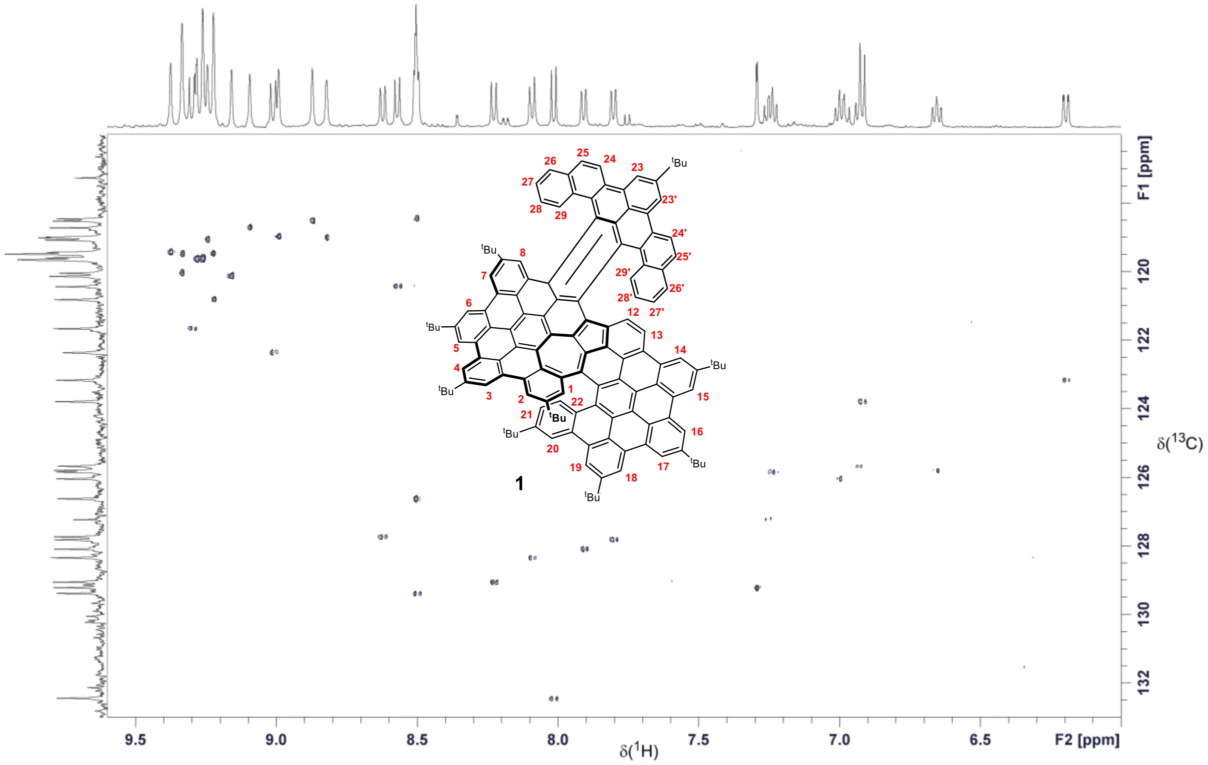

Figure S8. HSQC spectrum (region of aromatic CH groups) of **1** (500 MHz, CD_2Cl_2). The F1 axis depicts the DEPT135 spectrum.

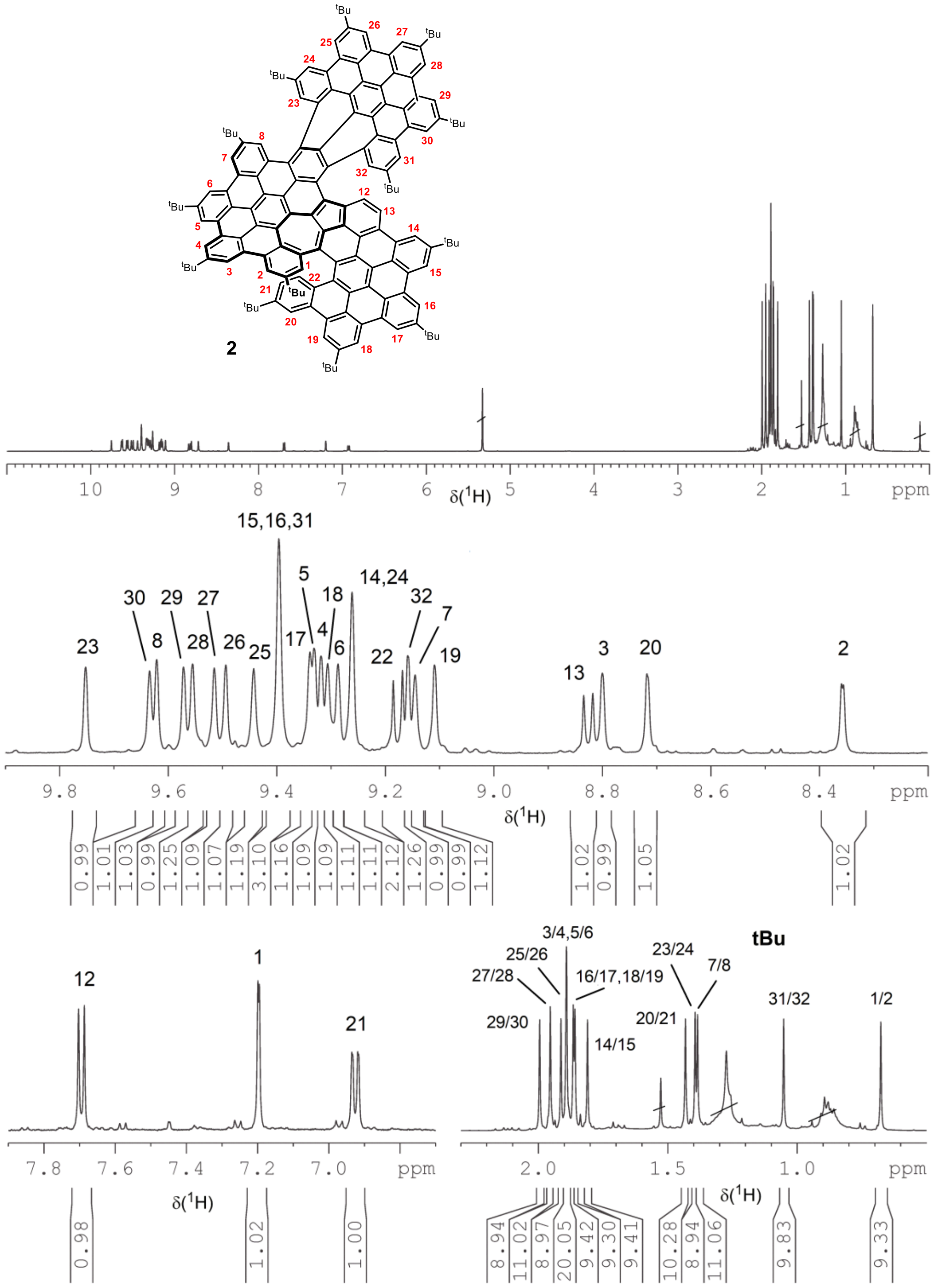

Figure S9. ^1H NMR spectrum of **2** and expanded regions (500 MHz, CD_2Cl_2).

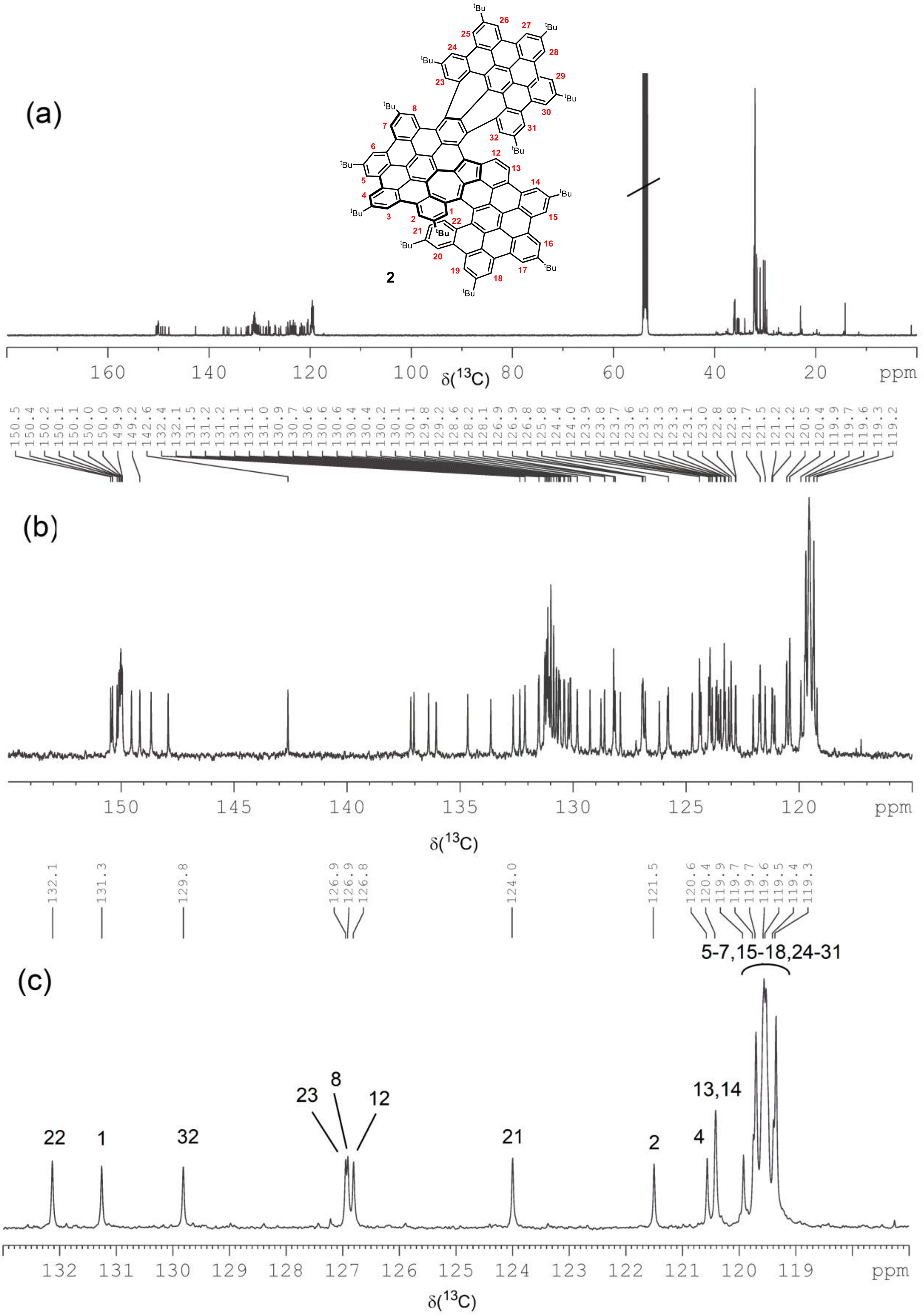

Figure S10. ^{13}C NMR spectrum of **2** (a), expanded region (b) and region of the DEPT135 spectrum (c) in CD_2Cl_2 (125 MHz).

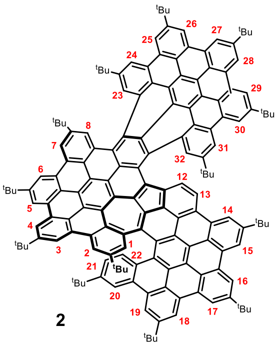

2

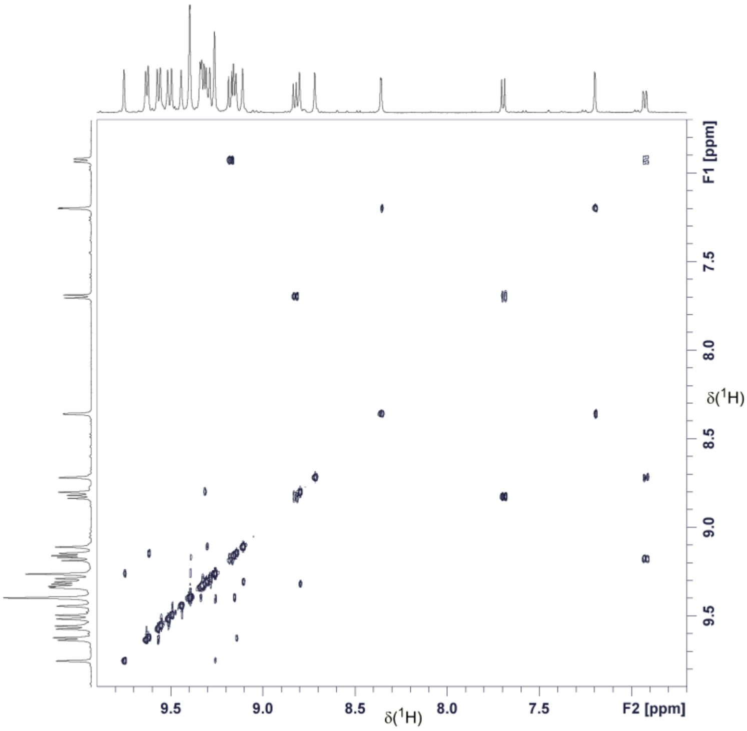

Figure S11. COSY spectrum (region of aromatic protons) of **2** (500 MHz, CD_2Cl_2).

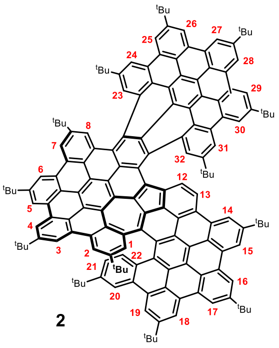

2

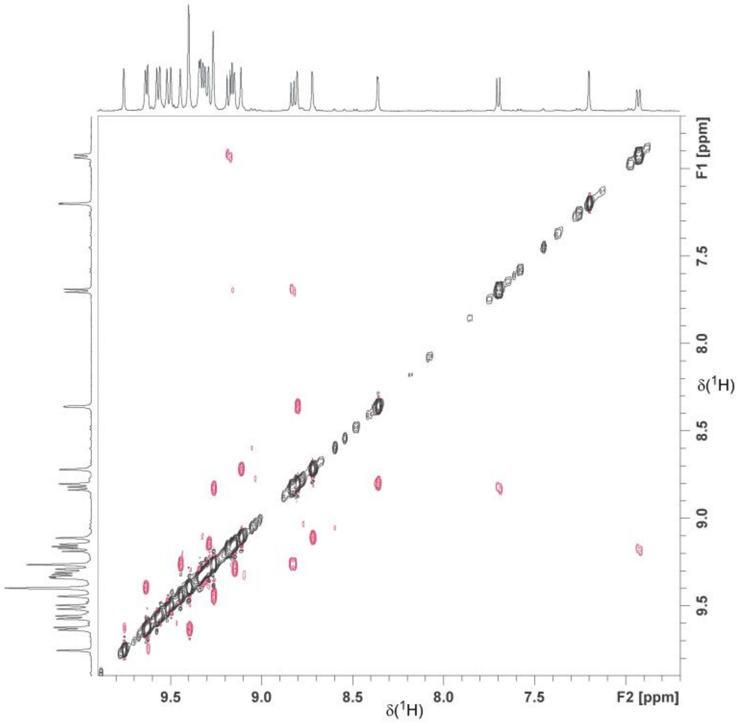

Figure S12. ROESY spectrum (region of aromatic protons) of **2** (500 MHz, CD_2Cl_2).

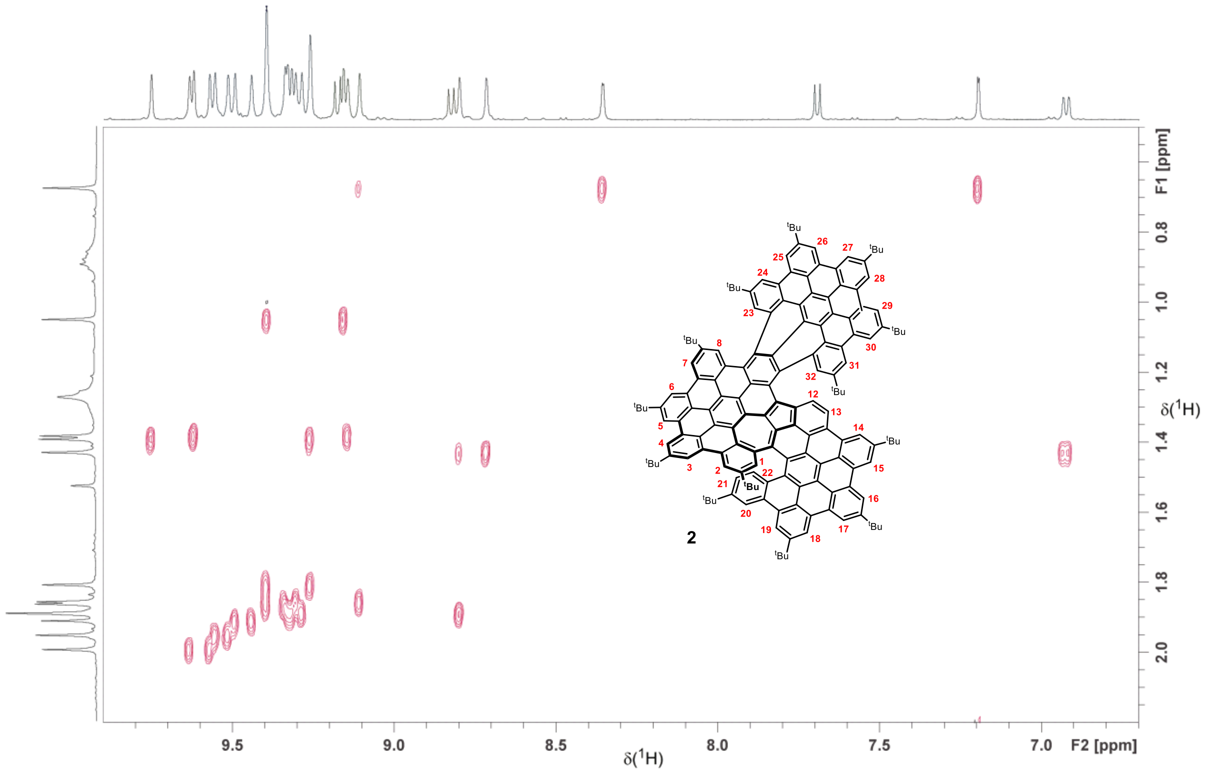

Figure S13. ROESY spectrum (correlations between CH_3 groups and aromatic protons) of **2** (500 MHz, CD_2Cl_2).

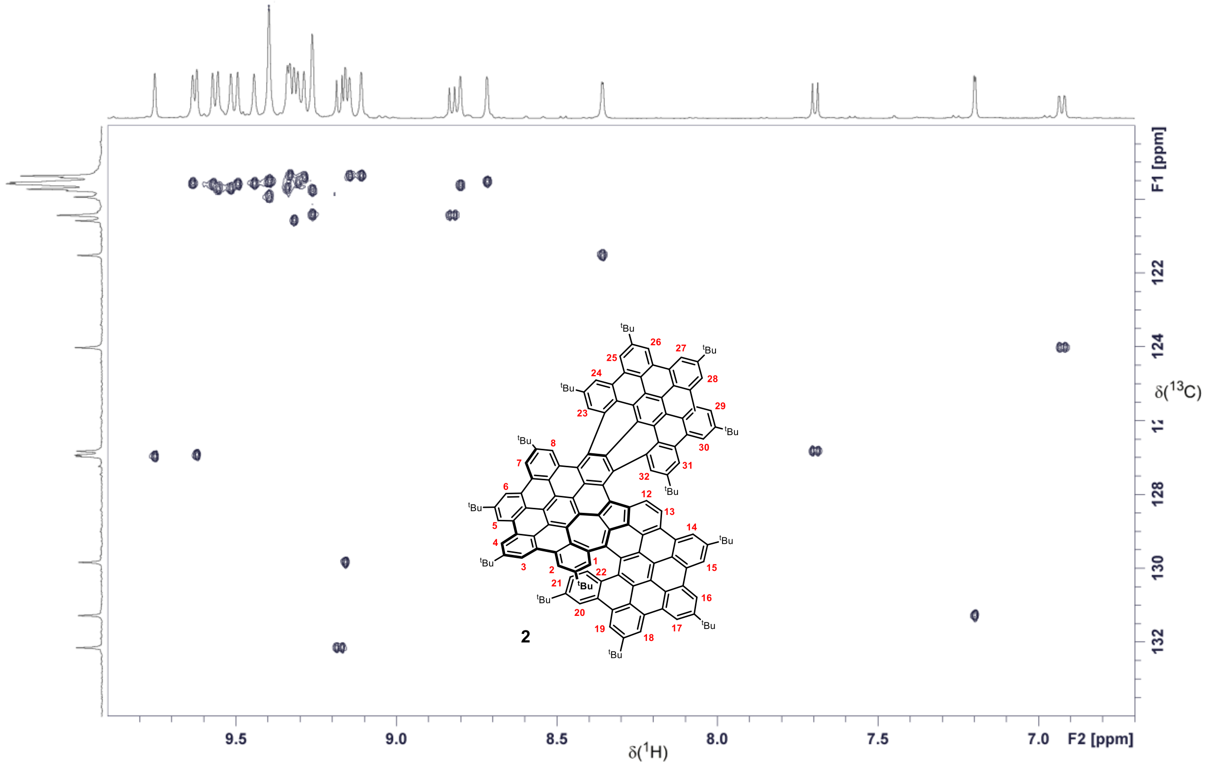

Figure S14. HSQC spectrum (region of aromatic CH groups) of **2** (500 MHz, CD_2Cl_2). The F1 axis depicts the DEPT135 spectrum.

5. X-ray crystallographic analysis of 1 and 2

Single crystals of **1** and **2** were obtained by slow vapor diffusion of the antisolvent methanol into dichlorobenzene and chlorobenzene, respectively. X-ray diffraction data collection was carried out at the BESSY storage ring (BL14.2, Berlin-Adlershof, Germany).[2] XDSAPP2.0 suite was employed for data processing.[3-4] The structure was solved by direct methods and refined by SHELXL-2019.[5] Hydrogen atoms were added geometrically and refined with a riding model. The X-ray crystallographic coordinates for **1** and **2** have been deposited at the Cambridge Crystallographic Data Centre (CCDC, 2473288 and 2473289). These data can be obtained free of charge from CCDC via http://www.ccdc.cam.ac.uk/data_request/cif

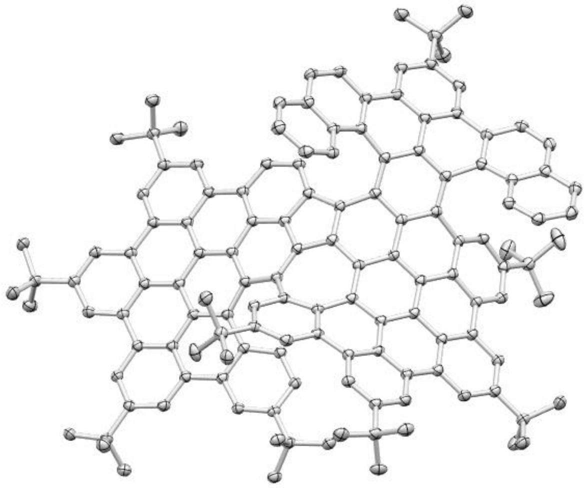

Figure S15. ORTEP diagram of (*M*, *M*, *P*)-**1**. Thermal ellipsoids are shown at the 30% probability level.

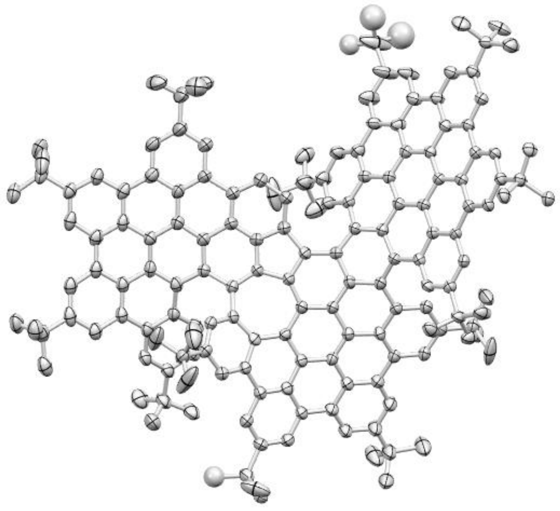

Figure S16. ORTEP diagram of (*M*, *M*, *M*)-**2**. Thermal ellipsoids are shown at the 30% probability level.

Table S1. Summary of crystal data and reflection collection parameters for compounds **1** and **2**.

**Crystal**	**1**	**2**
**Moiety formula**	$C_{150.40}H_{122.27}Cl_{4.14}$	$C_{164}H_{146}$
**Formula weight**	2076.16	2116.80
**Crystal size, mm**	0.02x0.02x0.03	0.07x0.07x0.08
**Crystal system**	monoclinic	monoclinic
**Space group**	$P2_1/n$	$P2_1/n$
***a*, Å**	19.670(4)	21.570(4)
***b*, Å**	16.890(3)	17.800(4)
***c*, Å**	35.110(7)	37.590(8)
***α*, deg**	90	90
***β*, deg**	97.08(3)	95.69(3)
***γ*, deg**	90	90
**Volume, Å^3**	11576(4)	14361(5)
Z	4	4
**$D_{calcd.}$, g cm^{-3}**	1.191	0.979
**F_{000}**	4380	4520
***T*, K**	100	100
**Radiation (λ, Å)**	Synchrotron (0.7999)	Synchrotron (0.7999)
**μ, mm^{-1}**	0.218	0.071
**2Θ range (°)**	2.546 to 61.674	2.45 to 60.902
**Index ranges**	$-25 \le h \le 25$, $-20 \le k \le 20$, $-42 \le l \le 42$	$-25 \le h \le 25$, $-21 \le k \le 21$, $-39 \le l \le 40$
**no. of collected reflections**	130848	160834
**no. of unique ref. (R_{int})**	23978 (0.0437)	27140 (0.0628)
**Data/restraints/parameters**	17016/0/1489	17165/44/1523
**R_1, wR_2 [obs $I>2\sigma(I)$]**	0.0810, 0.2243	0.1586, 0.4578
**R_1, wR_2 (all data)**	0.1107, 0.2442	0.1888, 0.4894
**residual peak/hole, e. Å^{-3}**	1.164/-0.643	0.929/-0.468
**Goodness-of-fit on F^2**	1.066	1.043
**CCDC**	2473288	2473289

Table S2. Summary of structural features. The structural parameters were obtained by X-ray single crystal diffraction analysis. [a] See ref. [6]. [b] See ref. [7]. [c] See ref. [8].

Compound	Sum of torsional angles (φ)	Angle between terminal rings (θ)	Distance between terminal rings (d)
[6]-**1**	94.2°	41.3°	4.00 Å
[6]-**1** heptagon	108.1°	35.8°	3.70 Å
[7]-**1** pentagon	110.8 °	60.2°	4.38 Å
[5]-**2**	72.6 °	64.2°	5.16 Å
[6]-**2** heptagon	106 °	33.3°	3.75 Å
[6]-**2** pentagon	78.9 °	53.6°	4.54 Å
carbon[5]helicene [a]	66.3°	47.3°	4.99 Å
carbon[6]helicene [b]	87.5°	59.5°	4.48 Å
carbon[7]helicene [c]	109.3 °	32.0°	3.80 Å

6. Determination of enantiopurity 1 by HPLC and configurational stability study

The racemic resolution was achieved using a Chiralpak® IA analytical chiral column. The column temperature was set at 25 °C and the flow rate was constant during operation (1 mL/min). **1**-*MMP* resists racemization at 100 ºC for 4 h. (Figure S15b).

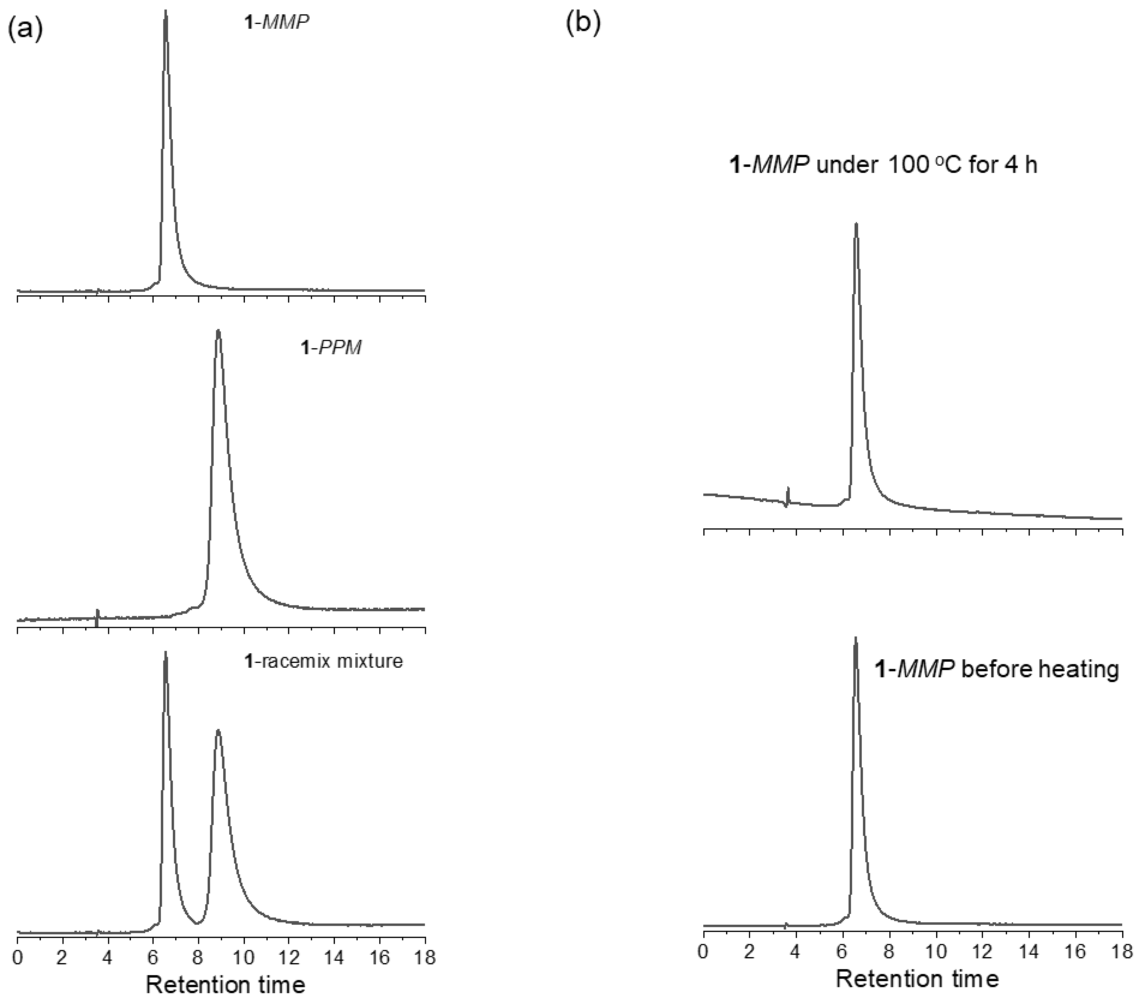

Figure S17. (a) HPLC traces (Hex/$CHCl_3$ 95:5) of **1**, (b) Thermostability measurement of **1**-*MMP* in toluene at 100 ºC for 4 h.

7. Photophysical study

7.1 Molar absorption coefficient of 1 and 2

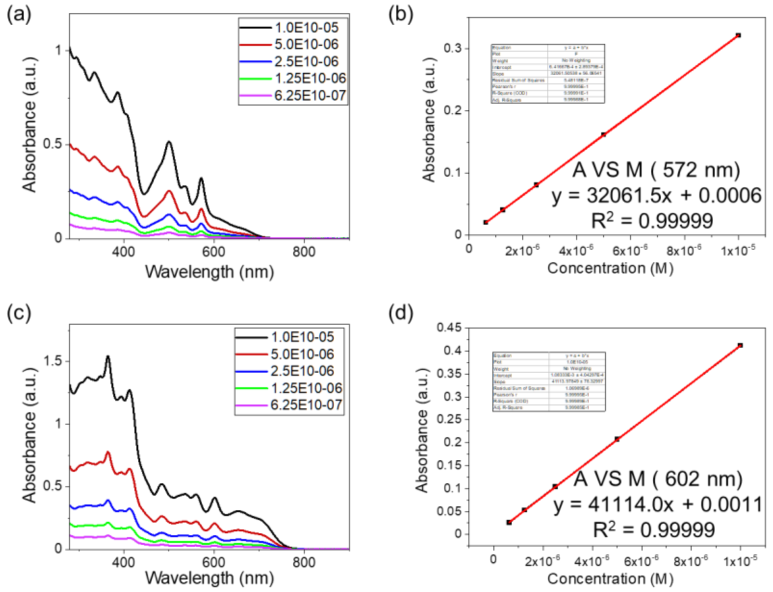

Figure S18. Absorbance spectra of **1** (a) and **2** (c) at different concentrations in CH_2Cl_2; Absorbance vs Concentration plot of compound **1** at ~572 nm (b) and **2** at ~602 nm (d), respectively.

7.2 Experimental and calculated ECD spectra

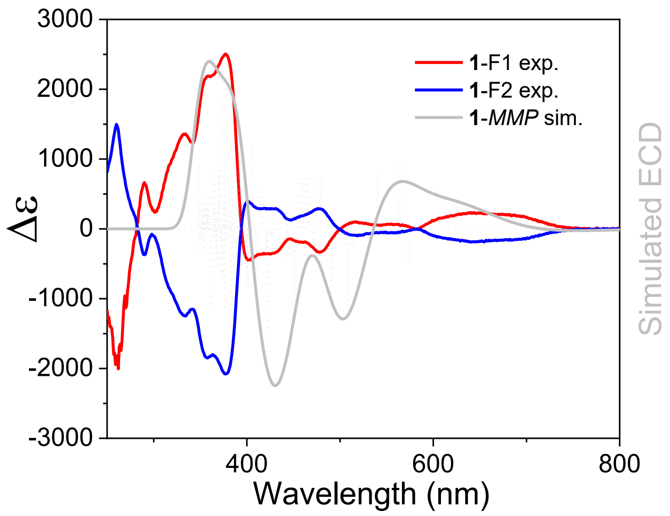

Figure S19. Experimental ECD spectra of **1**-F1 and **1**-F2 and comparison with the calculated ECD of **1**-*MMP*.

8. DFT calculations

All density functional theory (DFT) calculation was performed using the Gaussian 16 program.[9] The B3LYP functional was used for geometry optimization in the ground. All the geometry optimization was done in the gas phase and based on the single crystal structure. In order to simulate the UV-Vis spectra of the molecules TD-DFT calculations using B3LYP functional. For better comparison to the experimental absorption spectra the polarity of the solvent dichloromethane was added.

Anisotropy of the induced current density (ACID) plots were calculated by Herges's method.[10] Nucleus independent chemical shifts (NICS) values were calculated using the standard gauge invariant atomic orbital (GIAO)[11-12] method at B3LYP functional. All NICS values were averaged by two positions (above and below the plane) of each molecule.

TD-DFT calculations were carried out to simulate the UV-Vis spectra of compounds **1** and **2** by using the B3LYP functional and the 6-31G(d) basis set (Figure S18). The polarity of the solvent DCM was added for better comparability to the experimental absorption spectra. The simulated UV-Vis absorption matched well with the experimental results (Figure S18).

The NICS and ACID calculations were conducted to evaluate the aromaticity of their azulene-embedded asymmetric π-extended helical frameworks. The pentagons and heptagons are antiaromatic (**1**: ring E, +21.98 ppm, ring P, 7.80 ppm; **2**: ring E, +20.45 ppm, ring R, 6.15 ppm), while other rings exhibit aromaticity or nonaromatic features based on the NICS calculation (Figure S20). In addition, the ACID simulations also display a counter-clockwise ring current for the embedded azulene units in **1** and **2**, demonstrating their antiaromatic behavior (Figure S21).

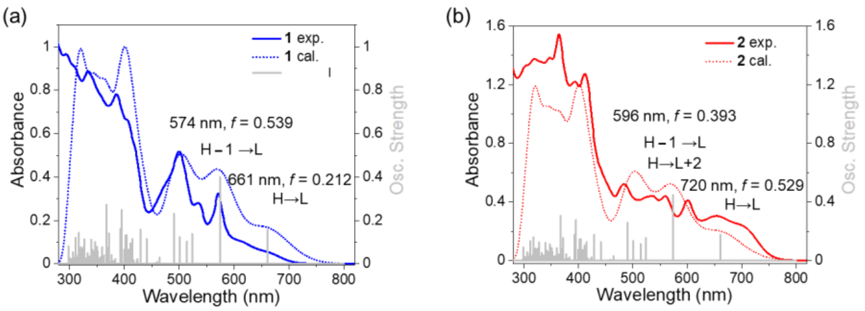

Figure S20. Comparison of experimental (blue), TD-DFT calculated (red) UV-Vis spectra and calculated oscillator strength (grey bar) of (a) **1** and (b) **2** in DCM at TD-B3LYP/6-31+G(d) level of theory.

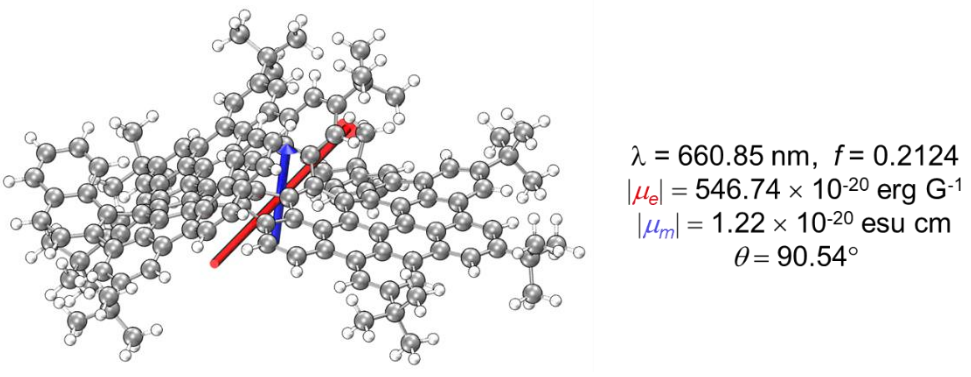

Figure S21. Transition dipole moments of 1 for the $S_0 \rightarrow S_1$ transition.

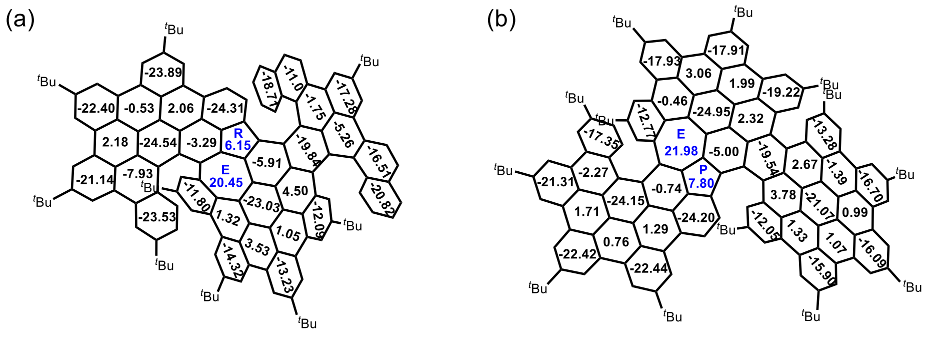

Figure S22. NICS(1)$_{zz}$ values of (a) **1** and (b) **2**, calculated at the GIAO-B3LYP/6-311+G(2d,p) level of theory.

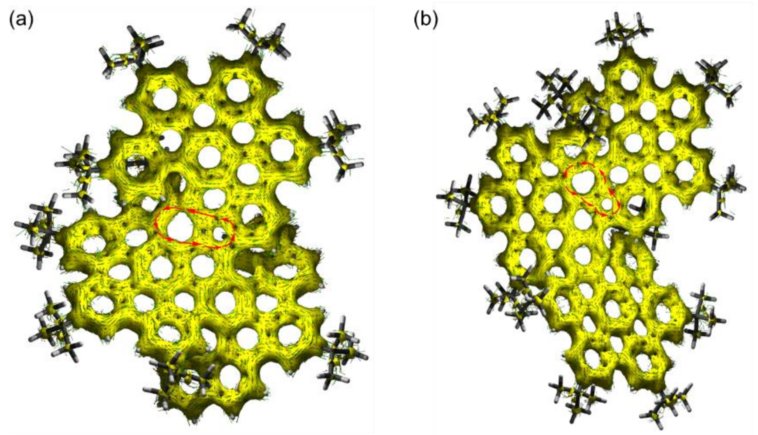

Figure S23. ACID plots of (a) **1** and (b) **2**. The isosurface value is 0.05 and the red arrows indicate diamagnetic (clockwise) ring current flow.

Table S3. TD-DFT calculation of **1** using B3LYP functional and 6-31G(d) basis set.

Excited state	Energy (eV)	Wavelength (nm)	Oscillator strength (f)	Description
1	1 .8761	660.85	0.2124	HOMO->LUMO 96.6%
2	2.1598	574.05	0.53920	HOMO-1>LUMO 93.6%
3	2.3672	523.76	0.1859	HOMO-2->LUMO 74.7%<br>HOMO->LUMO+2 13.9%<br>HOMO->LUMO+1 6.2%
4	2.4119	514.05	0.13950	HOMO->LUMO+1 87.1%
5	2.4695	502.06	0.14140	HOMO-3->LUMO 74.3%<br>HOMO-4->LUMO 14.1%
6	2.5278	490.48	0.02440	HOMO-4->LUMO 76.5%<br>HOMO-3->LUMO 17.0%
7	2.5934	478.08	0.2093	HOMO->LUMO+2 49.9%<br>HOMO->LUMO+3 11.9%<br>HOMO-1->LUMO+1 10.6%<br>HOMO-5->LUMO 7.9%
8	2.6975	459.63	0.02670	HOMO-5->LUMO 53.4%<br>HOMO->LUMO+2 17.0%<br>HOMO->LUMO+3 13.3%
9	2.7086	457.74	0.10460	HOMO-->LUMO+3 31.8%<br>HOMO-1->LUMO+1 15.0%<br>HOMO->LUMO+2 12.3%<br>HOMO-6->LUMO 5.6 %
10	2.7428	452.04	0.03580	HOMO-6->LUMO 76.2%<br>HOMO-2->LUMO+1 6.0%<br>HOMO-1->LUMO+1 5.8%

Table S4. TD-DFT calculation of **2** using B3LYP functional and 6-31G(d) basis set.

Excited state	Energy (eV)	Wavelength (nm)	Oscillator strength (f)	Description
1	1.7230	719.58	0.52850	HOMO->LUMO 97.9%
2	2.0801	596.05	0.39630	HOMO-1>LUMO 89.6%<br>HOMO->LUMO+2 5.2%
3	2.1461	577.72	0.20400	HOMO->LUMO+1 93.1%
4	2.2303	555.91	0.1598	HOMO-2>LUMO+1 87.8%<br>HOMO->LUMO+2 6.8%
5	2.4695	502.06	0.14140	HOMO-3->LUMO 74.3%<br>HOMO-4->LUMO 14.1%
6	2.5278	490.48	0.02440	HOMO-4->LUMO 76.5%<br>HOMO-3->LUMO 17.0%
7	2.5934	478.08	0.2093	HOMO->LUMO+2 49.9%<br>HOMO->LUMO+3 11.9%<br>HOMO-1->LUMO+1 10.6%<br>HOMO-5->LUMO 7.9%
8	2.6975	459.63	0.02670	HOMO-5->LUMO 53.4%<br>HOMO->LUMO+2 17.0%<br>HOMO->LUMO+3 13.3%
9	2.7086	457.74	0.10460	HOMO-->LUMO+3 31.8%<br>HOMO-1->LUMO+1 15.0%<br>HOMO->LUMO+2 12.3%<br>HOMO-6->LUMO 5.6 %
10	2.7428	452.04	0.03580	HOMO-6->LUMO 76.2%<br>HOMO-2->LUMO+1 6.0%<br>HOMO-1->LUMO+1 5.8%

9. NMR spectra

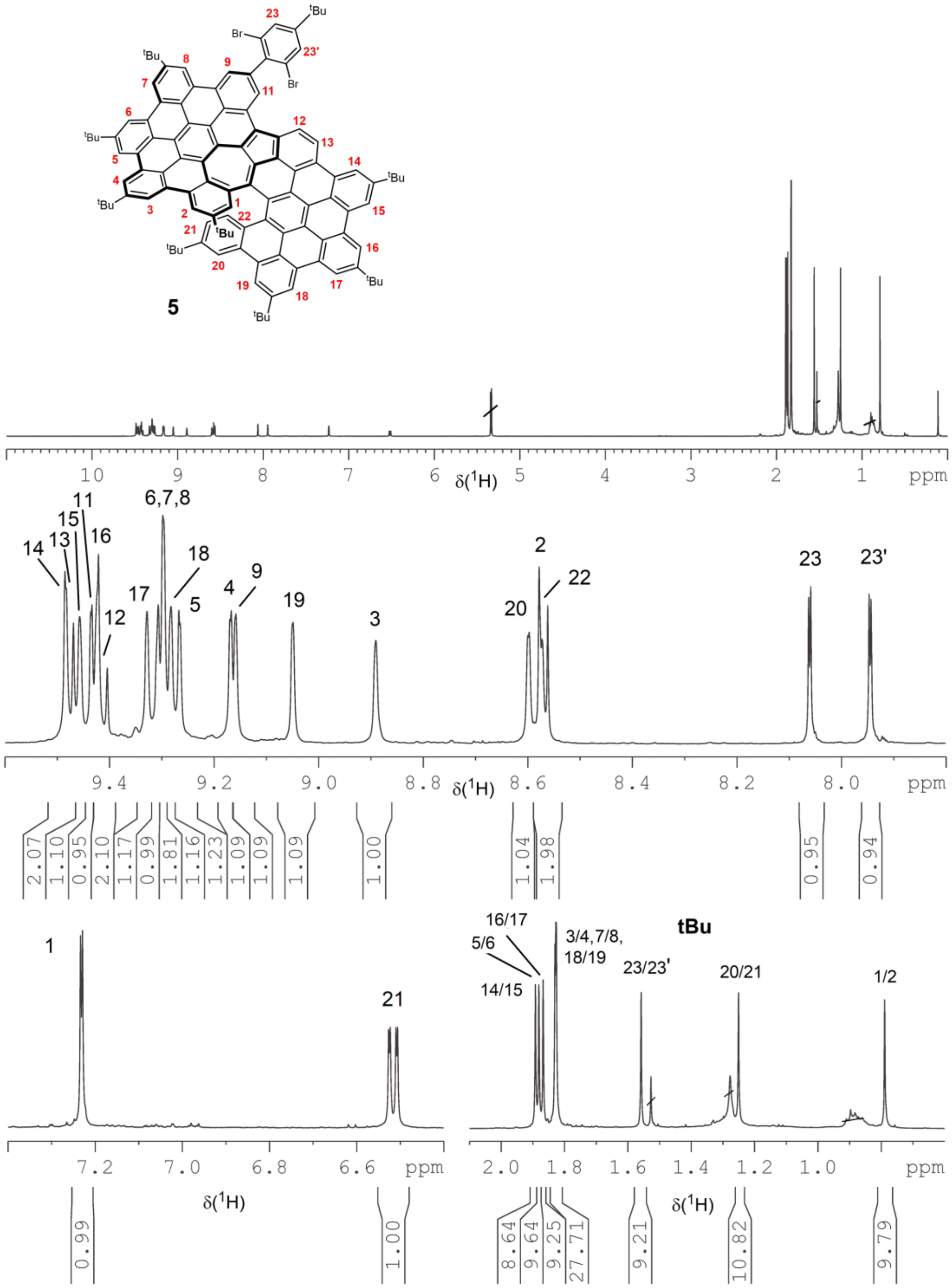

Figure S24. ^{1}H NMR spectrum of **5** and expanded regions (500 MHz, CD_2Cl_2).

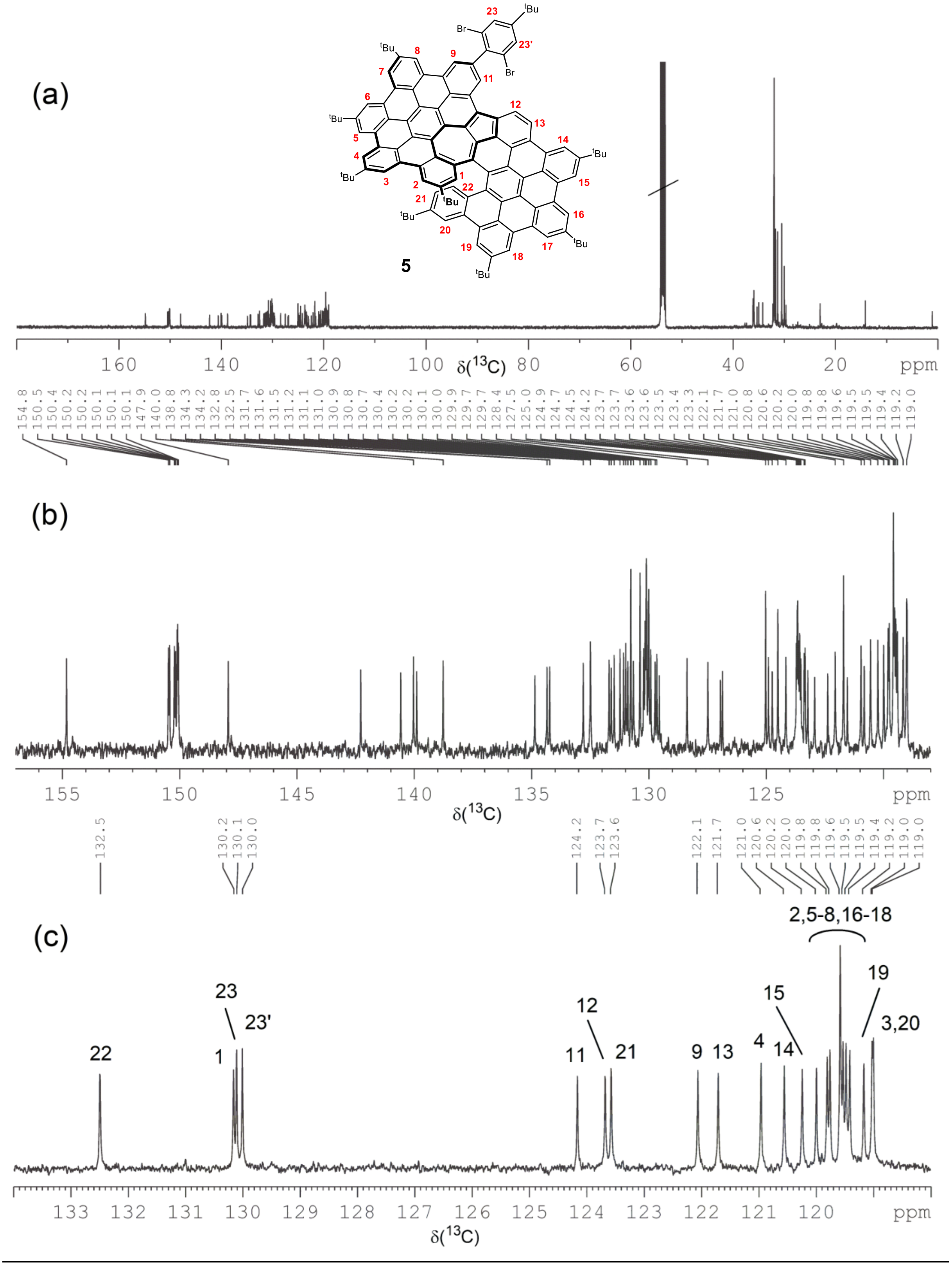

Figure S25. ^{13}C NMR spectrum of **5** (a), expanded region (b) and region of the DEPT135 spectrum (c) in CD_2Cl_2 (125 MHz).

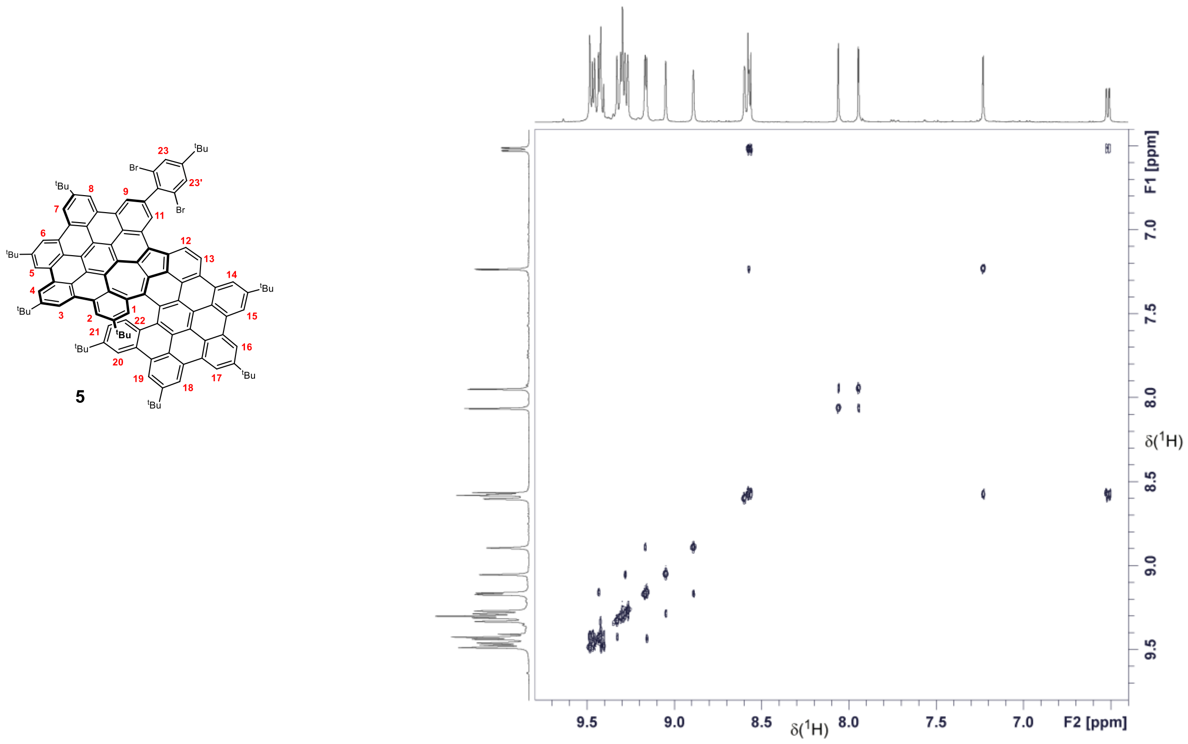

Figure S26. COSY spectrum (region of aromatic protons) of **5** (500 MHz, CD_2Cl_2).

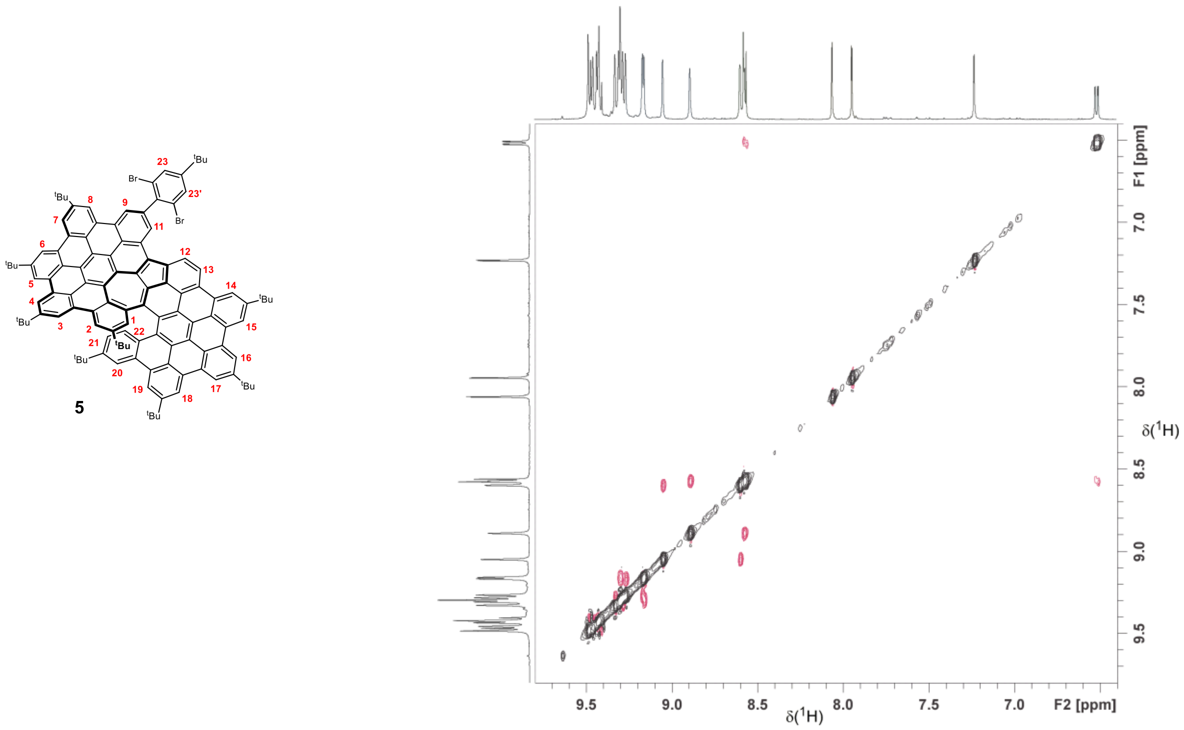

Figure S27. ROESY spectrum (region of aromatic protons) of **5** (500 MHz, CD_2Cl_2).

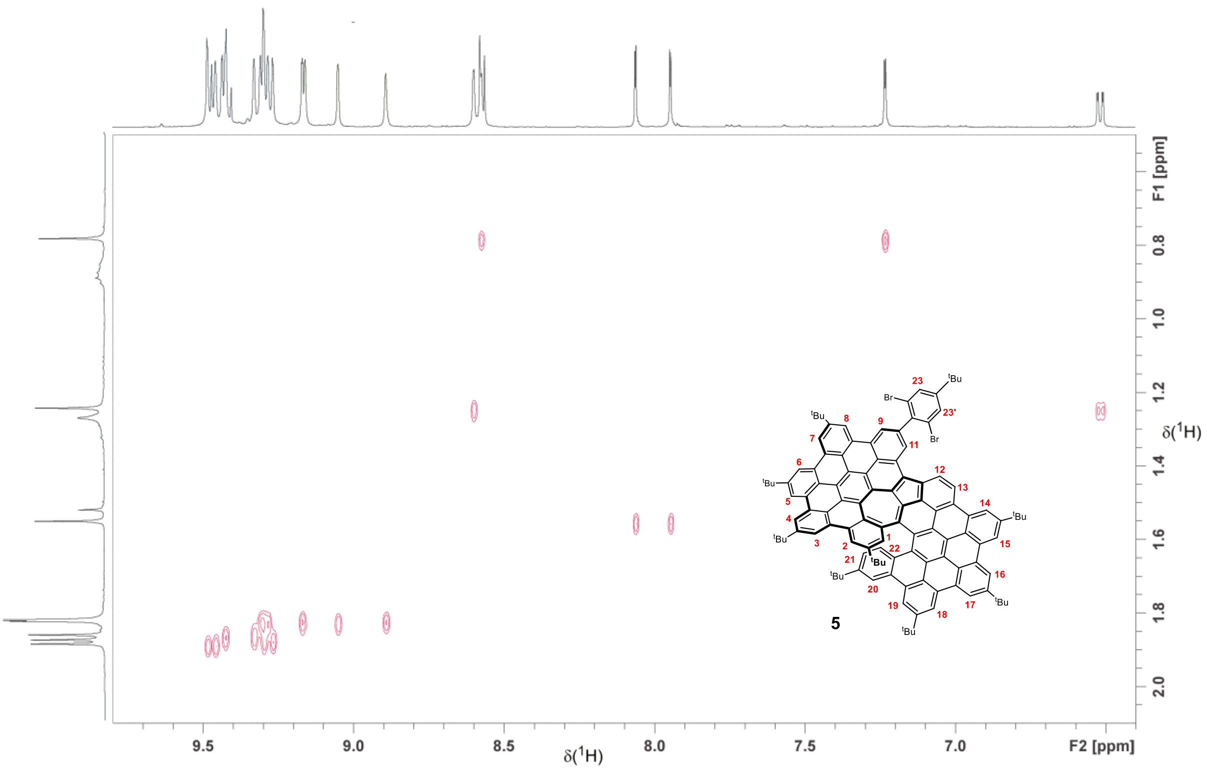

Figure S28. ROESY spectrum (correlations between CH_3 groups and aromatic protons) of **5** (500 MHz, CD_2Cl_2).

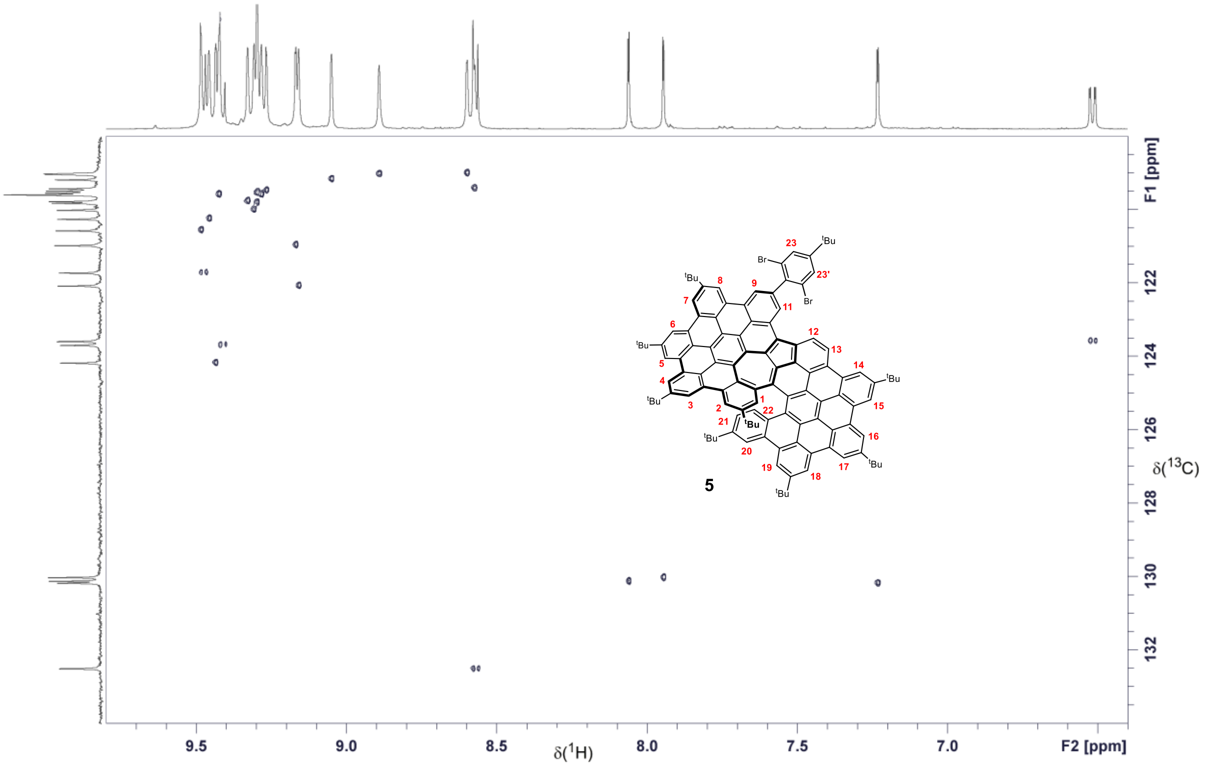

Figure S29. HSQC spectrum (region of aromatic CH groups) of **5** (500 MHz, CD_2Cl_2). The F1 axis depicts the DEPT135 spectrum.

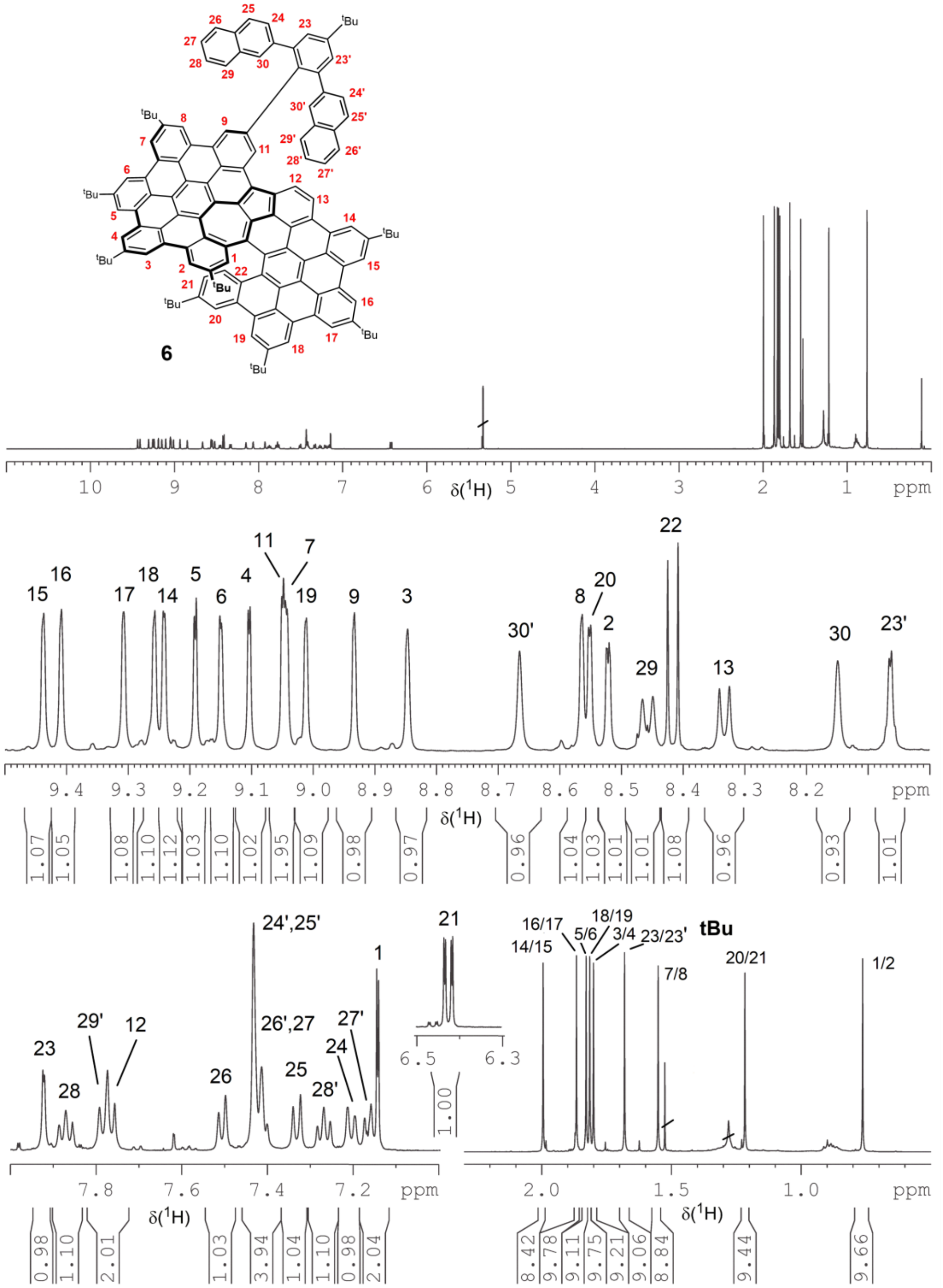

Figure S30. ^{1}H NMR spectrum of **6** and expanded regions (500 MHz, CD_2Cl_2).

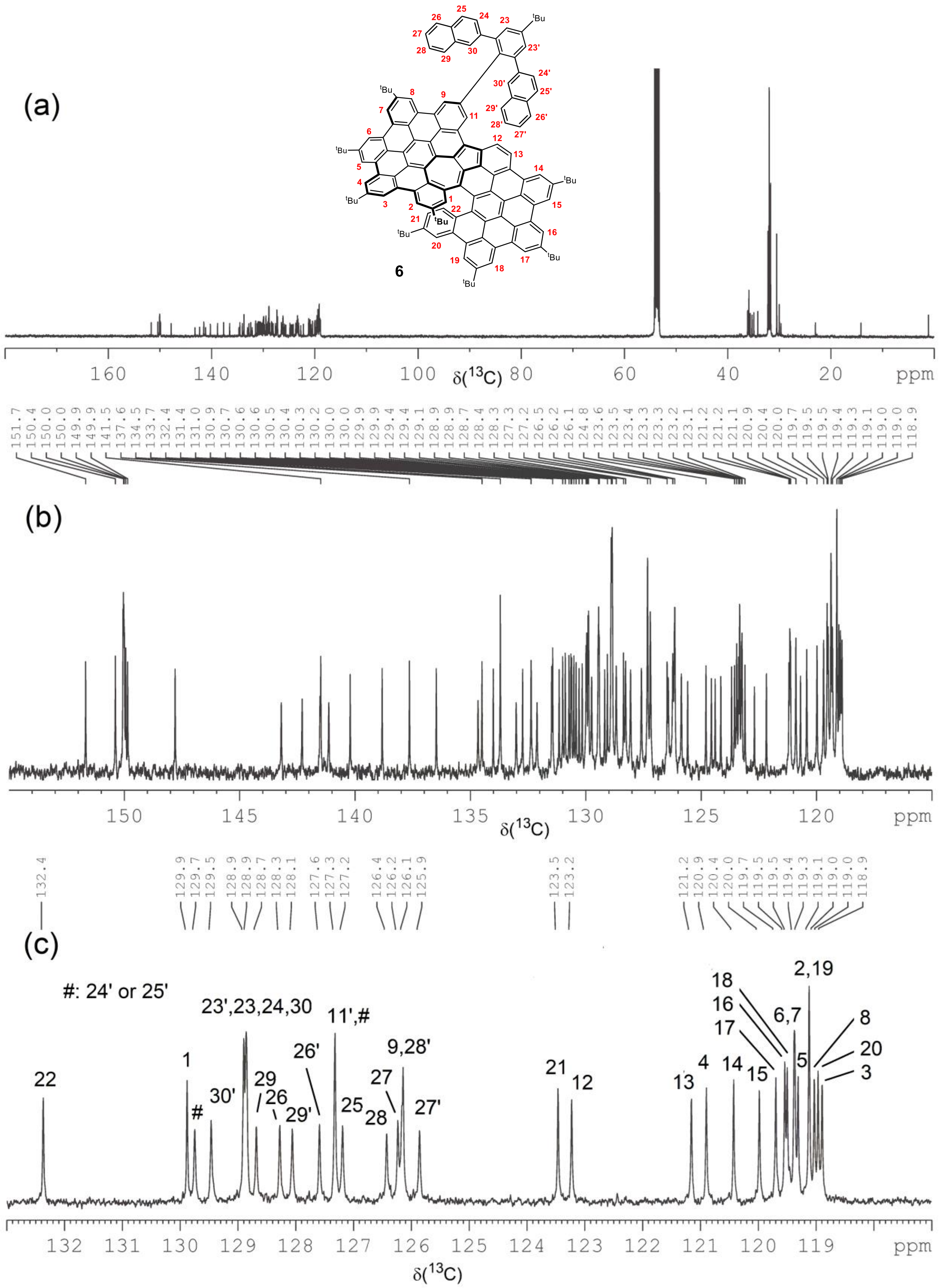

Figure S31. ^{13}C NMR spectrum of **6** (a), expanded region (b) and region of the DEPT135 spectrum (c) in CD_2Cl_2 (125 MHz).

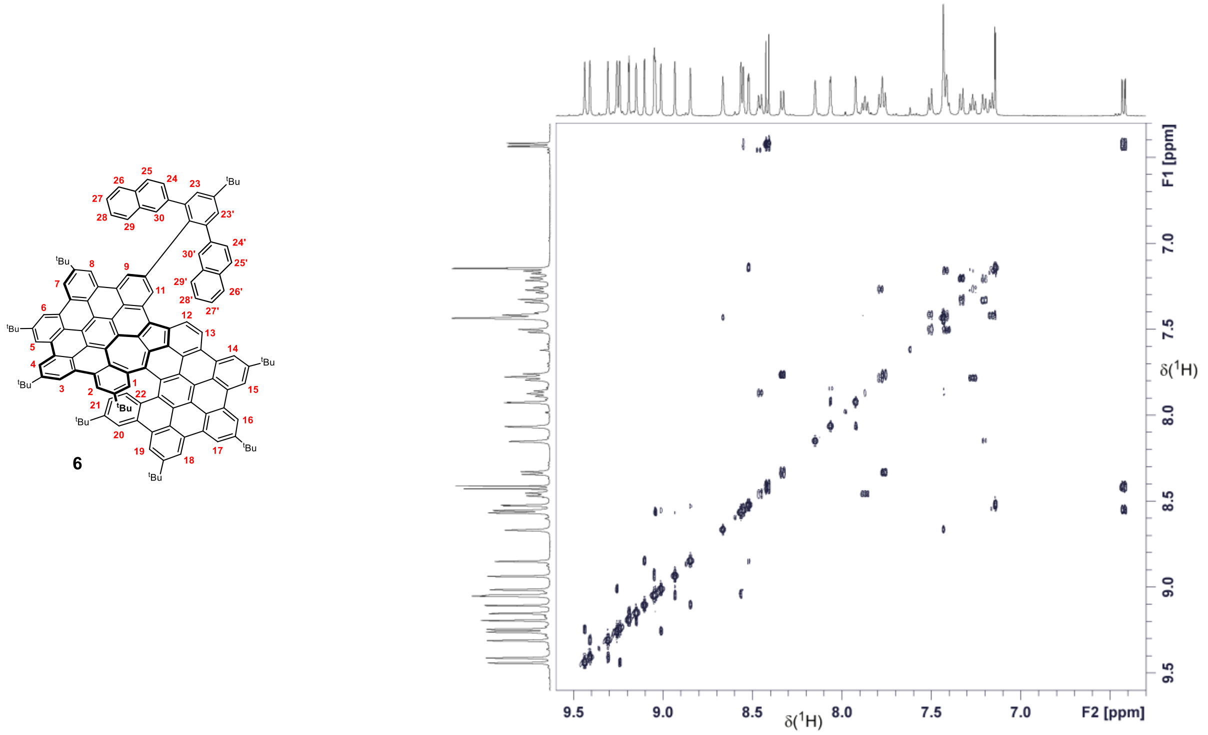

Figure S32. COSY spectrum (region of aromatic protons) of **6** (500 MHz, CD_2Cl_2).

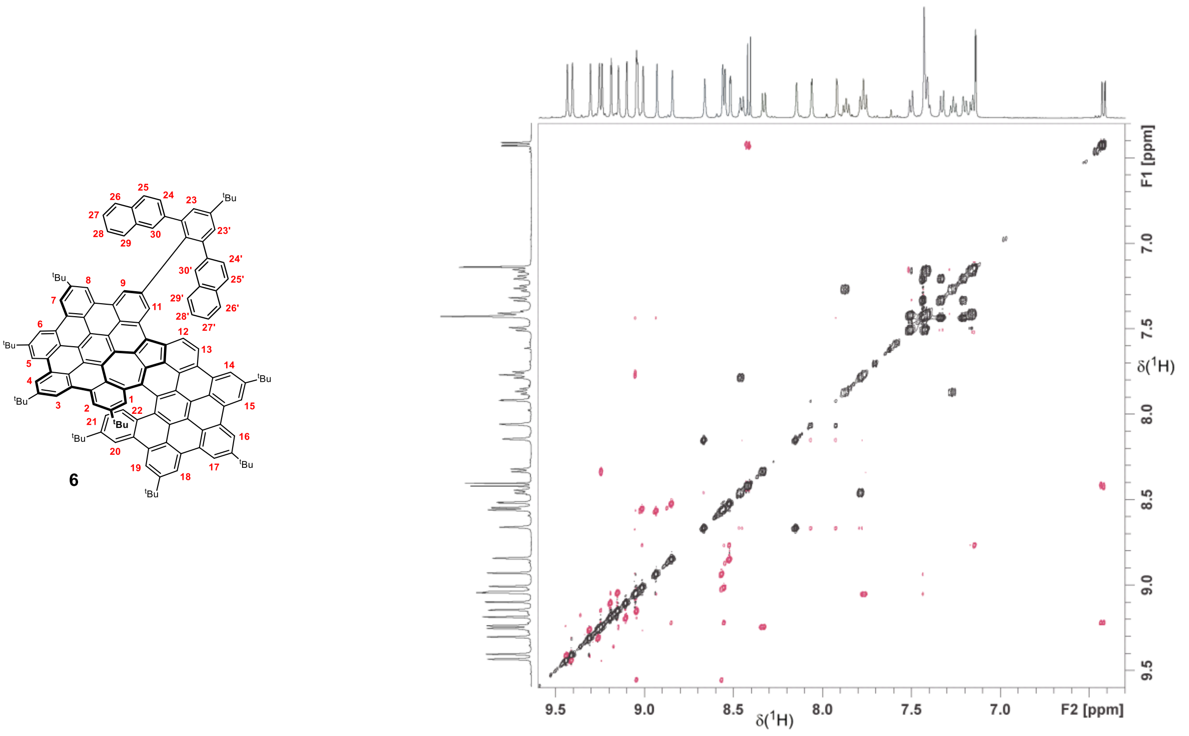

Figure S33. ROESY (red correlations)/EXSY (black correlations) spectrum (region of aromatic protons) of **6** (500 MHz, CD_2Cl_2).

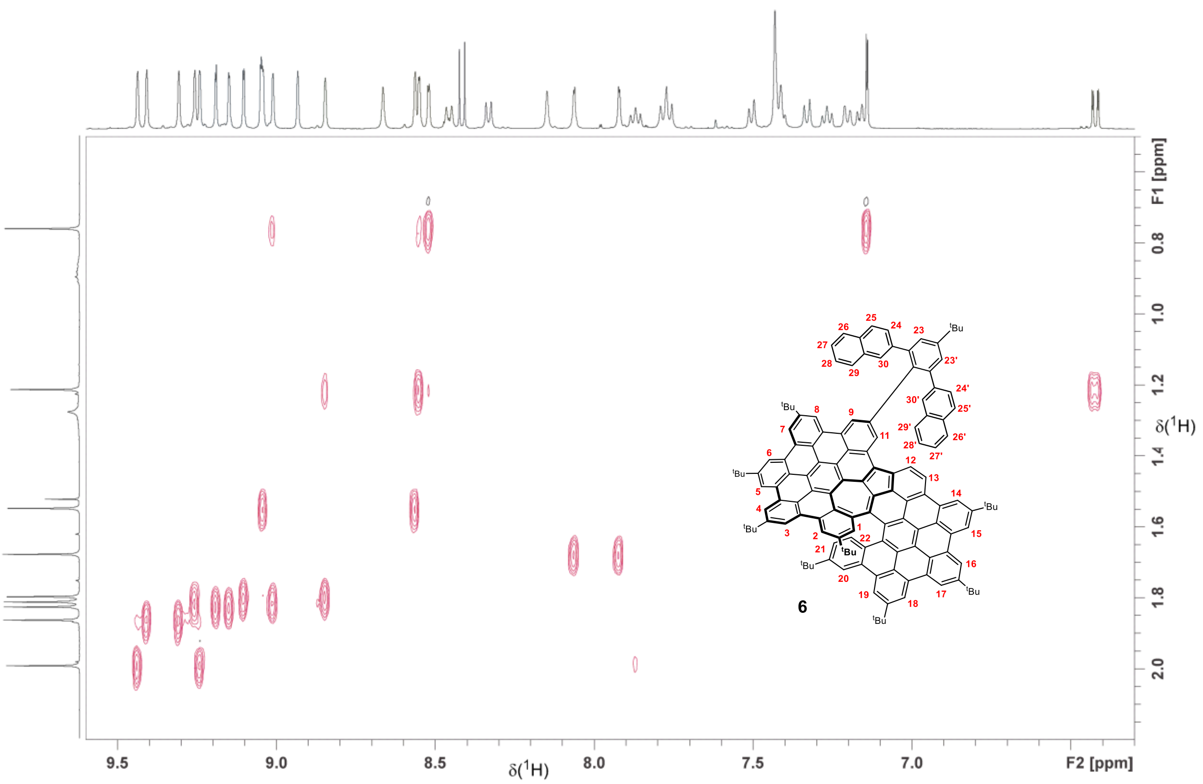

Figure S34. ROESY spectrum (correlations between CH_3 groups and aromatic protons) of **6** (500 MHz, CD_2Cl_2).

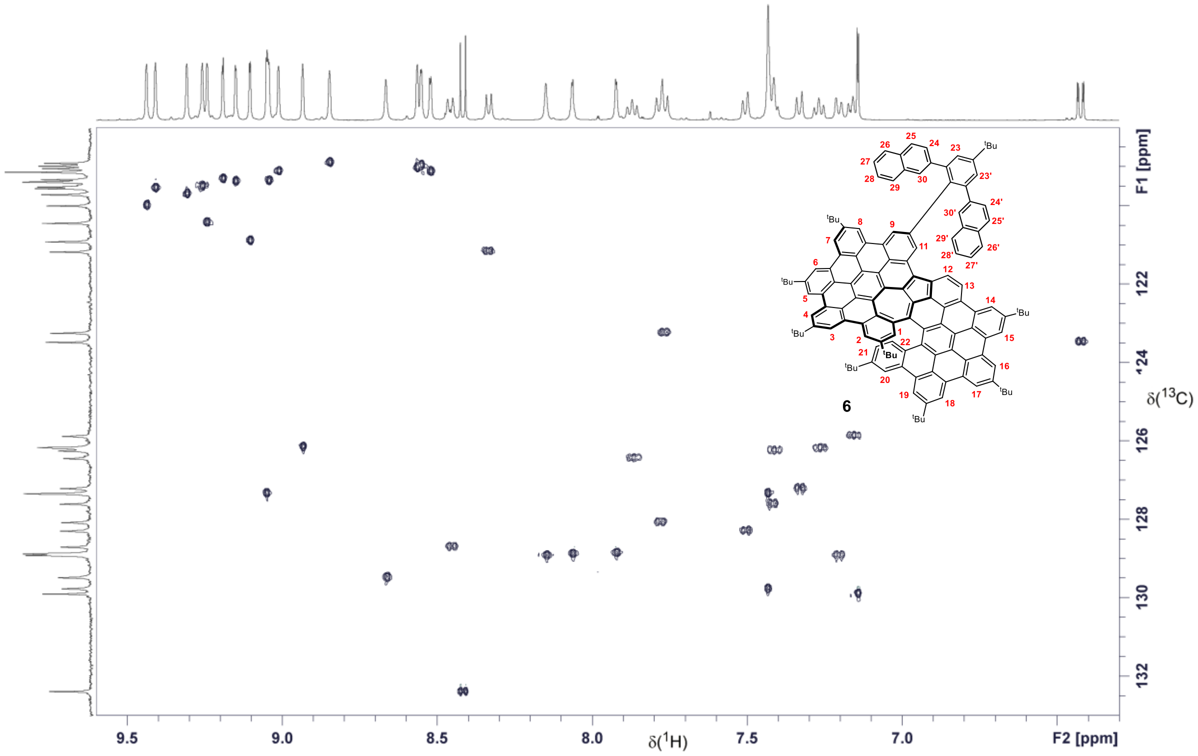

Figure S35. HSQC spectrum (region of aromatic CH groups) of **6** (500 MHz, CD_2Cl_2). The F1 axis depicts the DEPT135 spectrum.

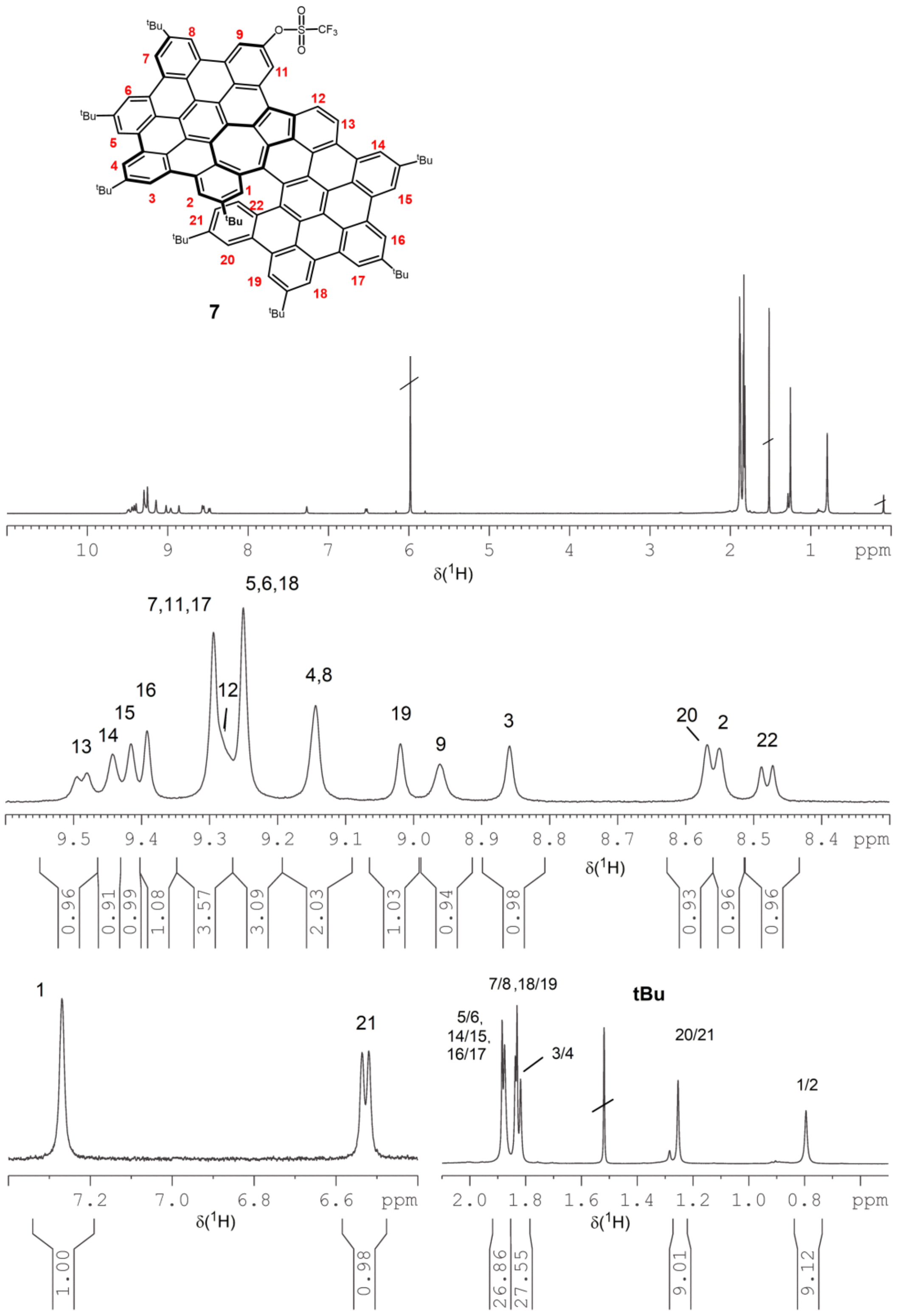

Figure S36. ^{1}H NMR spectrum of **7** and expanded regions (500 MHz, $C_2D_2Cl_4$, 60°C).

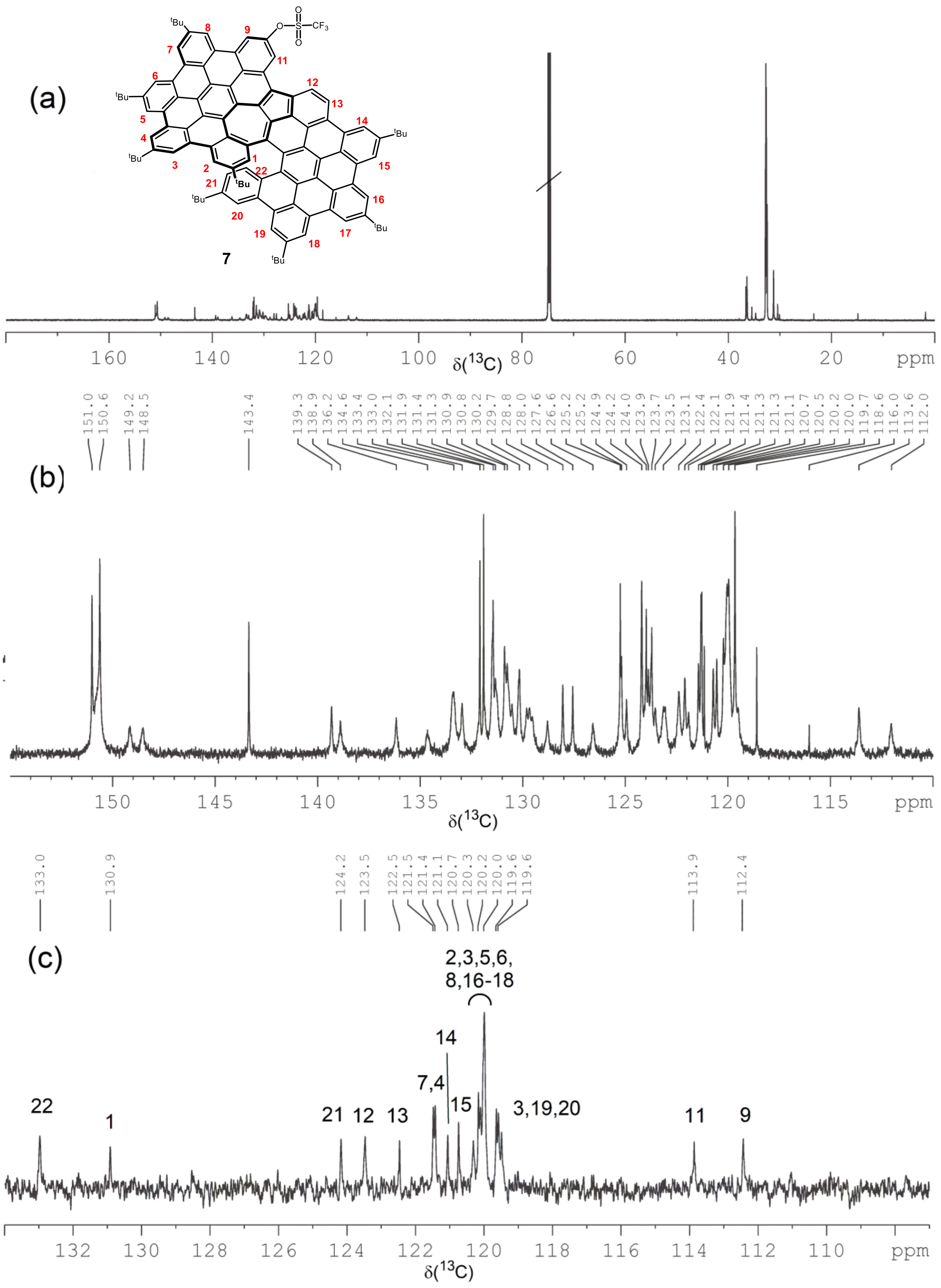

Figure S37. ^{13}C NMR spectrum of **7** (a), expanded region (b) and region of the DEPT135 spectrum (c) in $C_2D_2Cl_4$ at 60°C (125 MHz).

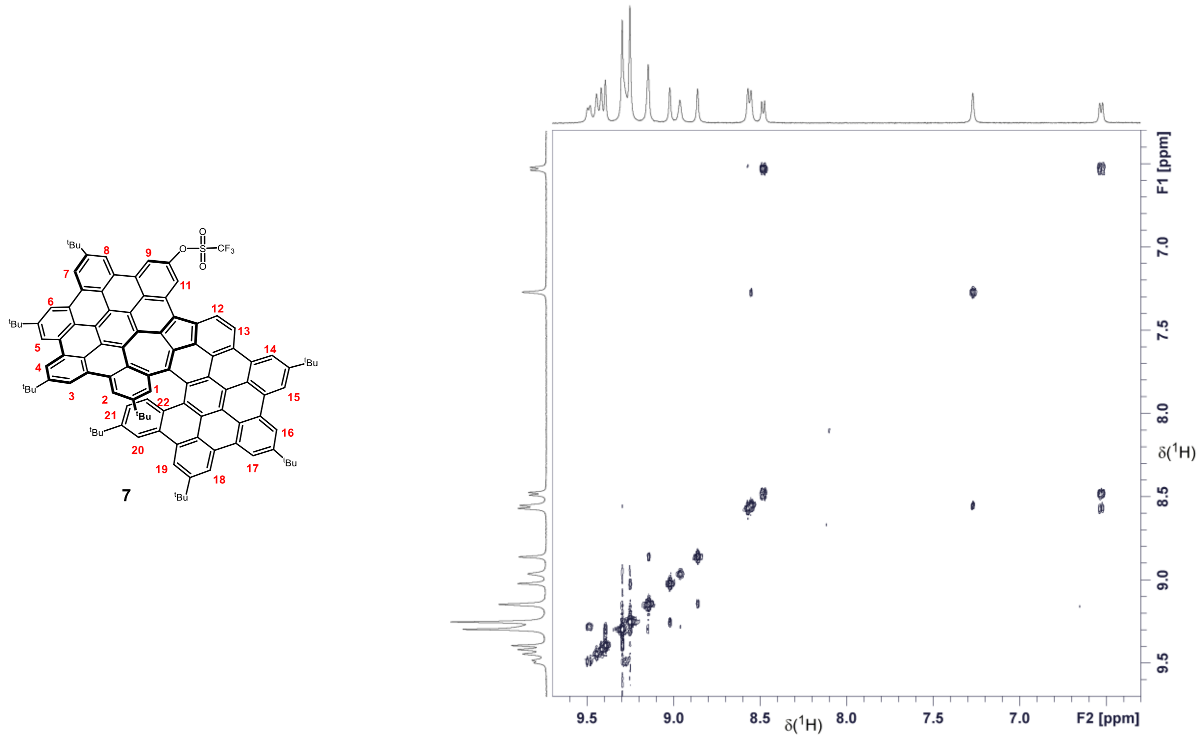

Figure S38. COSY spectrum (region of aromatic protons) of **7** (500 MHz, $C_2D_2Cl_4$, 60°C).

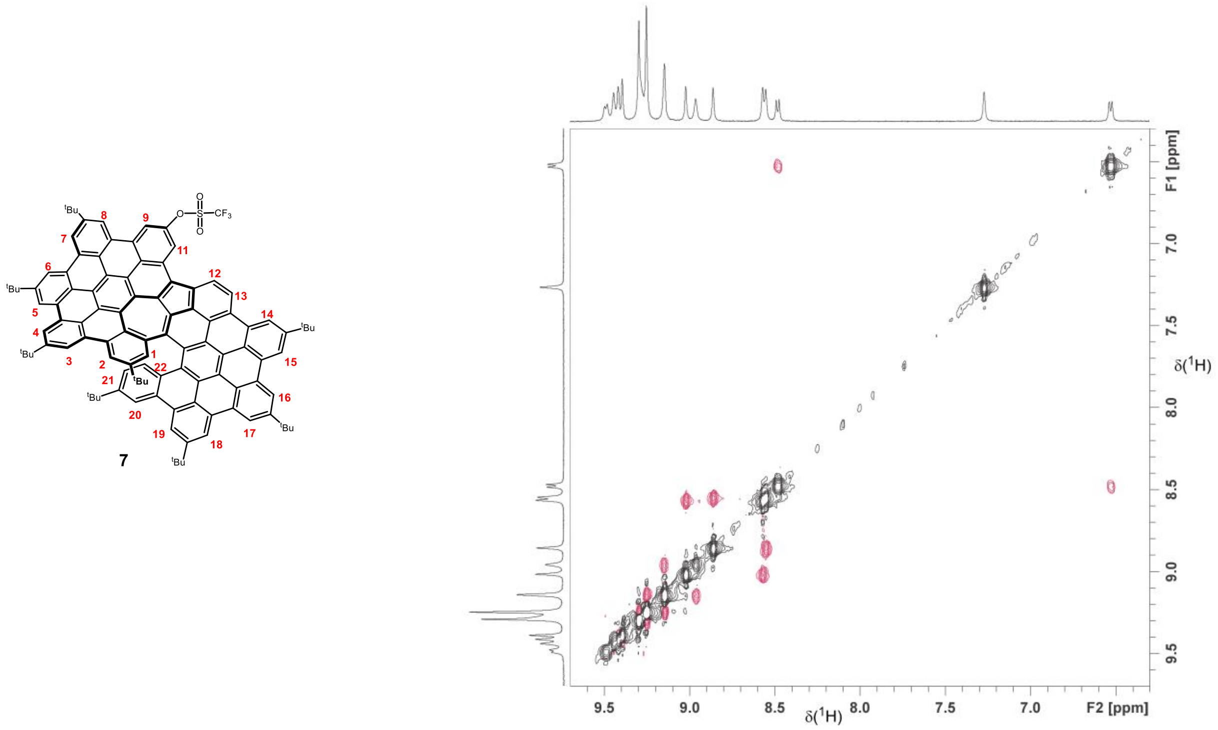

Figure S39. ROESY spectrum (region of aromatic protons) of **7** (500 MHz, $C_2D_2Cl_4$, 60°C).

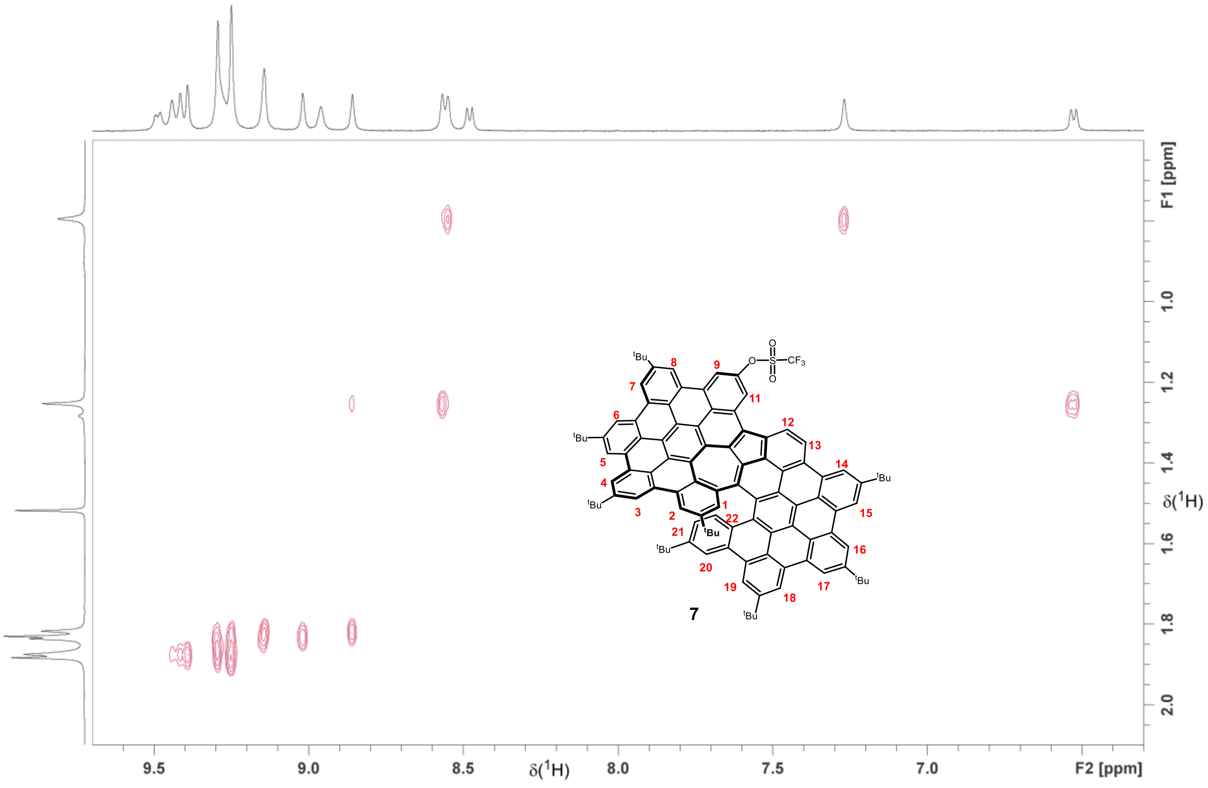

Figure S40. ROESY spectrum (correlations between CH_3 groups and aromatic protons) of **7** (500 MHz, $C_2D_2Cl_4$, 60°C).

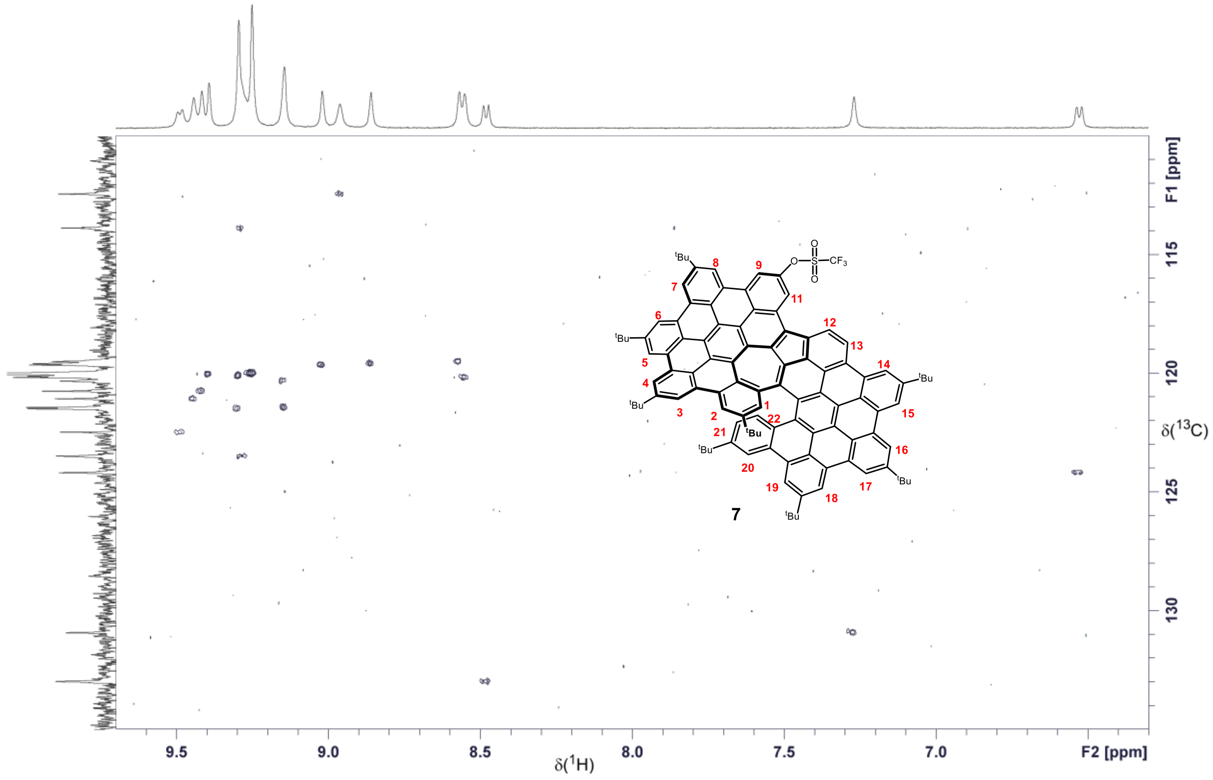

Figure S41. HSQC spectrum (region of aromatic CH groups) of **7** (500 MHz, $C_2D_2Cl_4$, 60°C). The F1 axis depicts the DEPT135 spectrum.

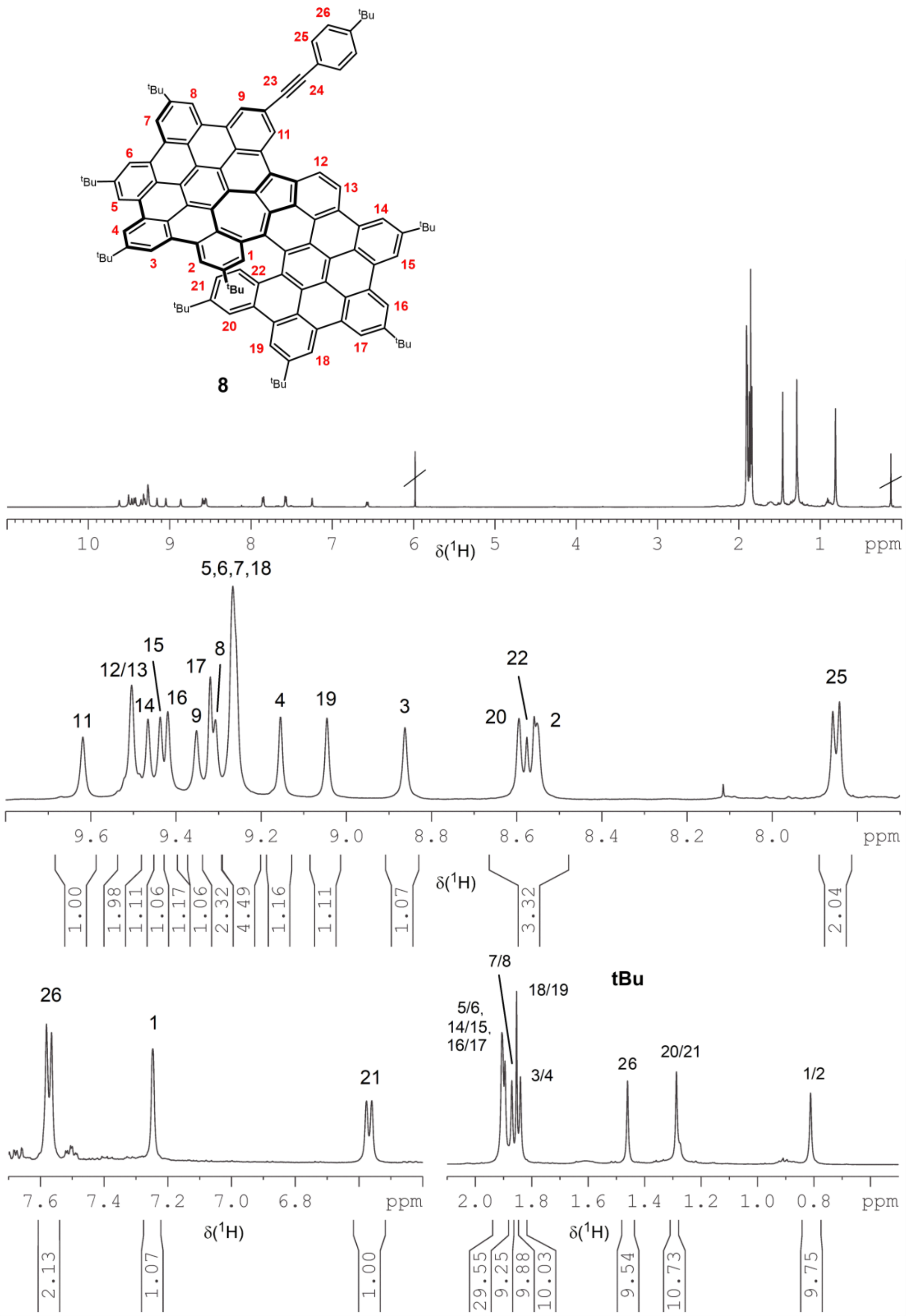

Figure S42. ^{1}H NMR spectrum of **8** and expanded regions (500 MHz, $C_2D_2Cl_4$).

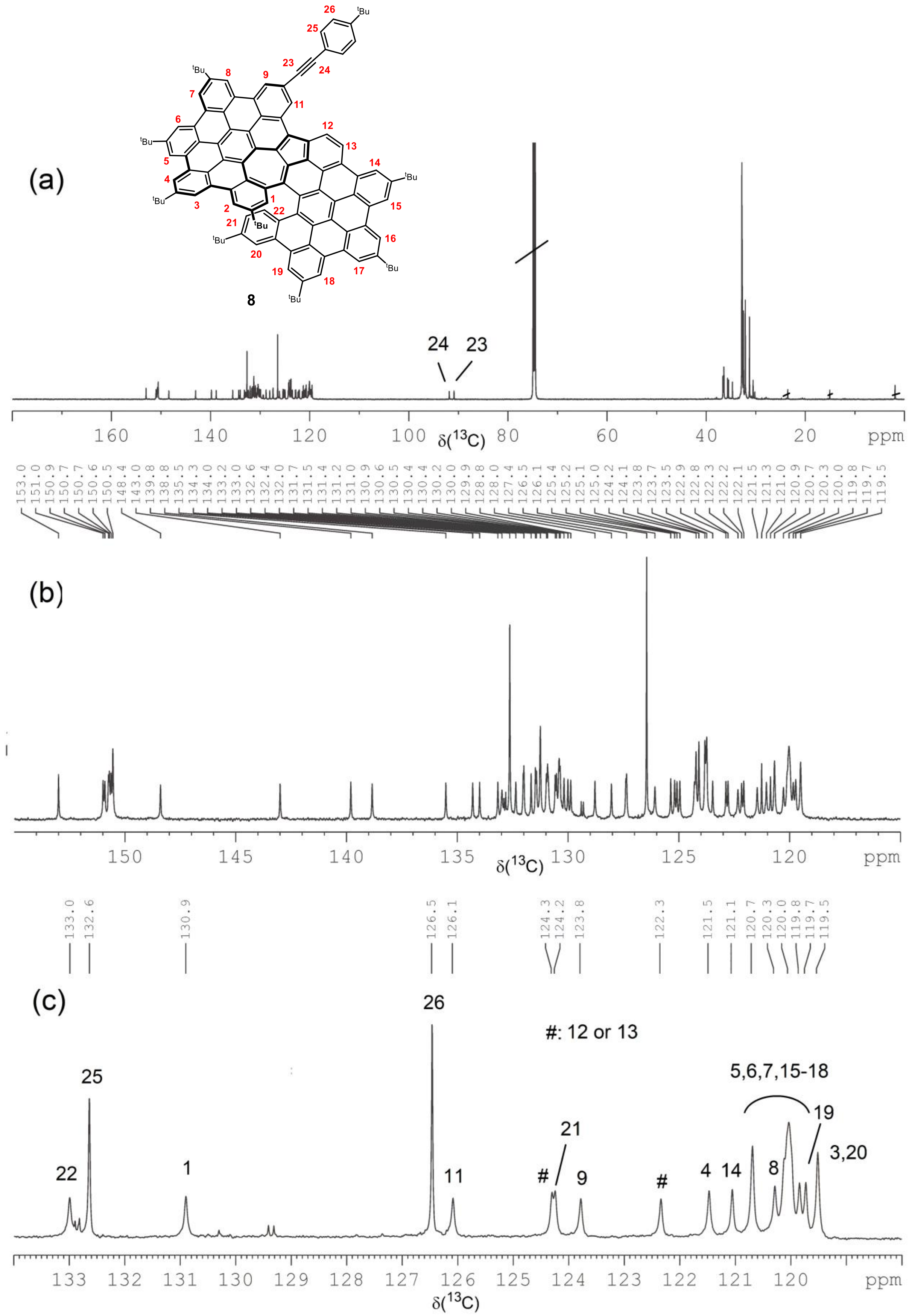

Figure S43. ^{13}C NMR spectrum of **8** (a), expanded region (b) and region of the DEPT135 spectrum (c) in $C_2D_2Cl_4$ (125 MHz).

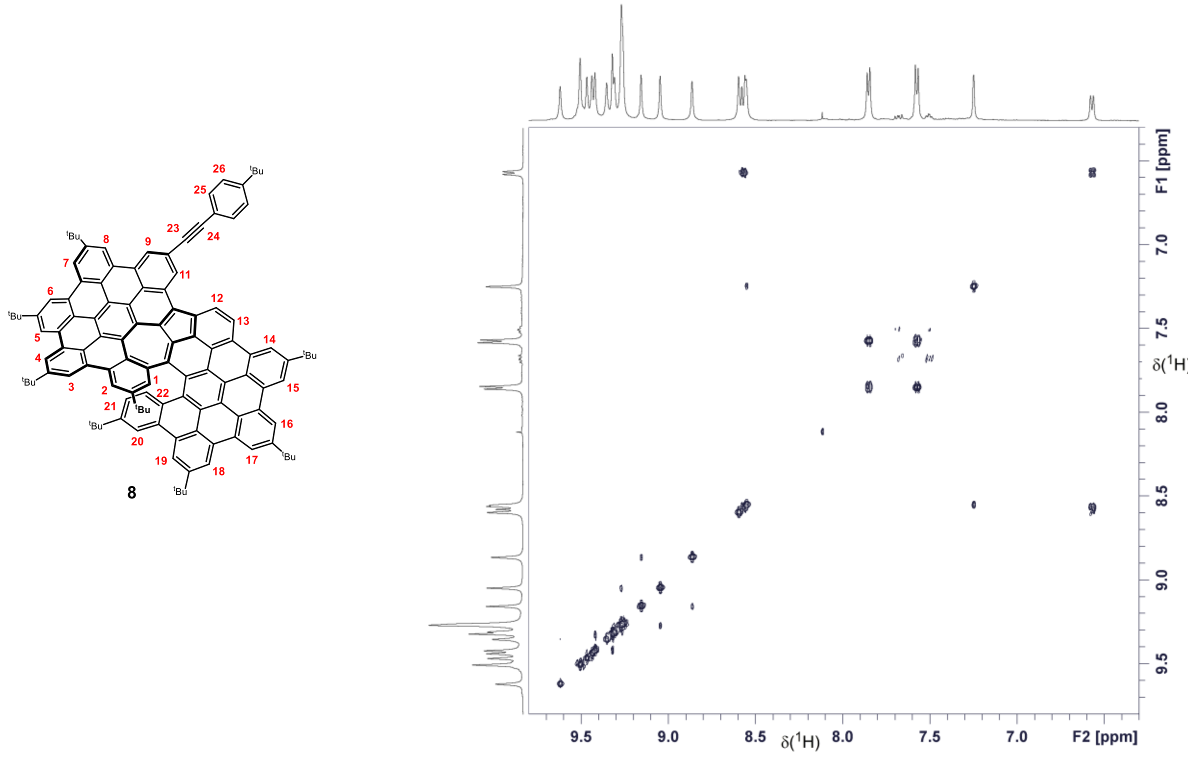

Figure S44. COSY spectrum (region of aromatic protons) of **8** (500 MHz, $C_2D_2Cl_4$).

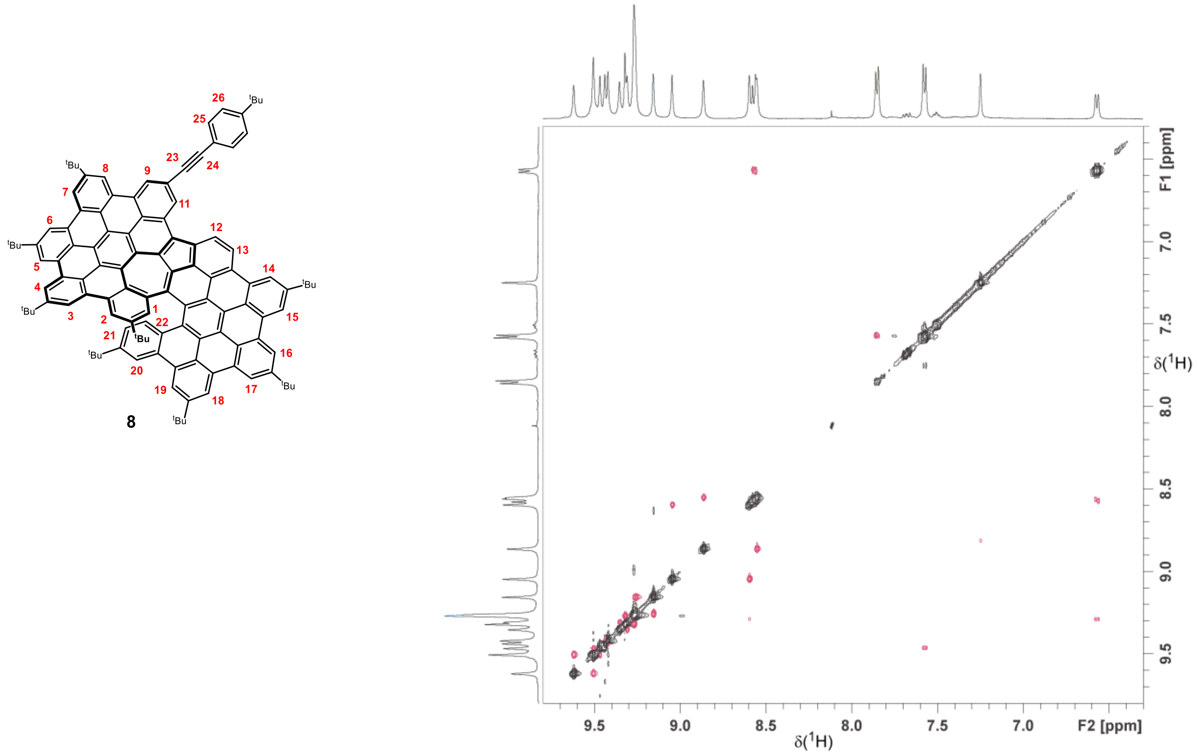

Figure S45. ROESY spectrum (region of aromatic protons) of **8** (500 MHz, $C_2D_2Cl_4$).

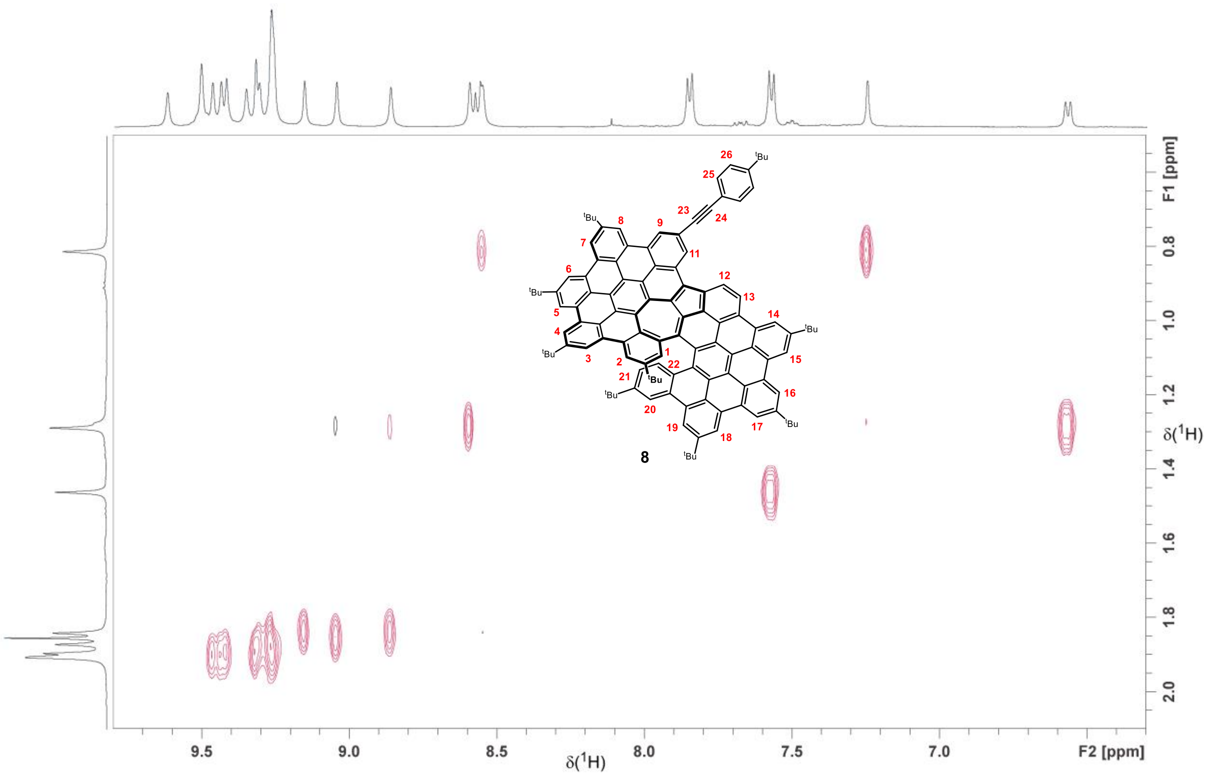

Figure S46. ROESY spectrum (correlations between CH_3 groups and aromatic protons) of **8** (500 MHz, $C_2D_2Cl_4$).

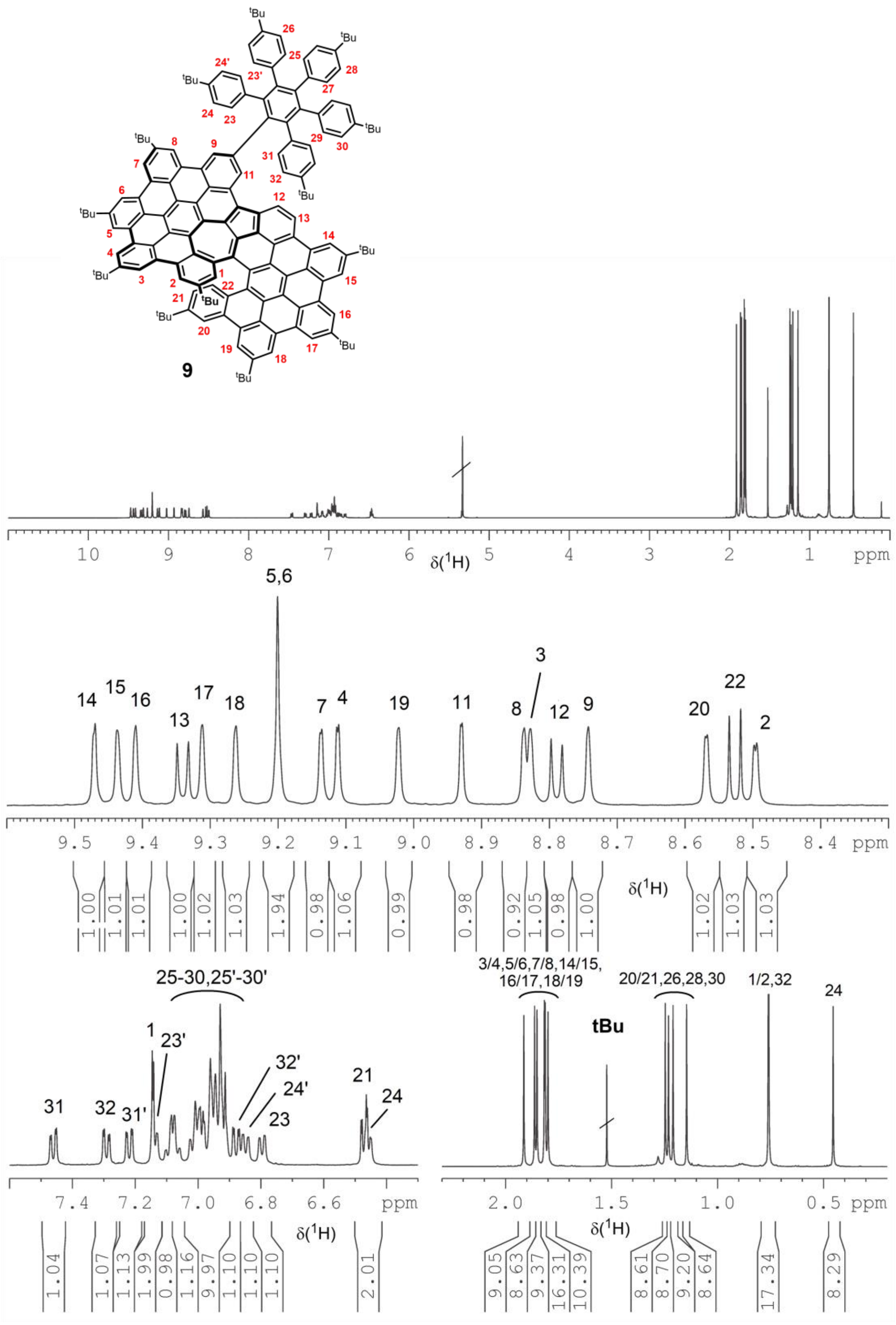

Figure S47. ^{1}H NMR spectrum of **9** and expanded regions (500 MHz, CD_2Cl_2).

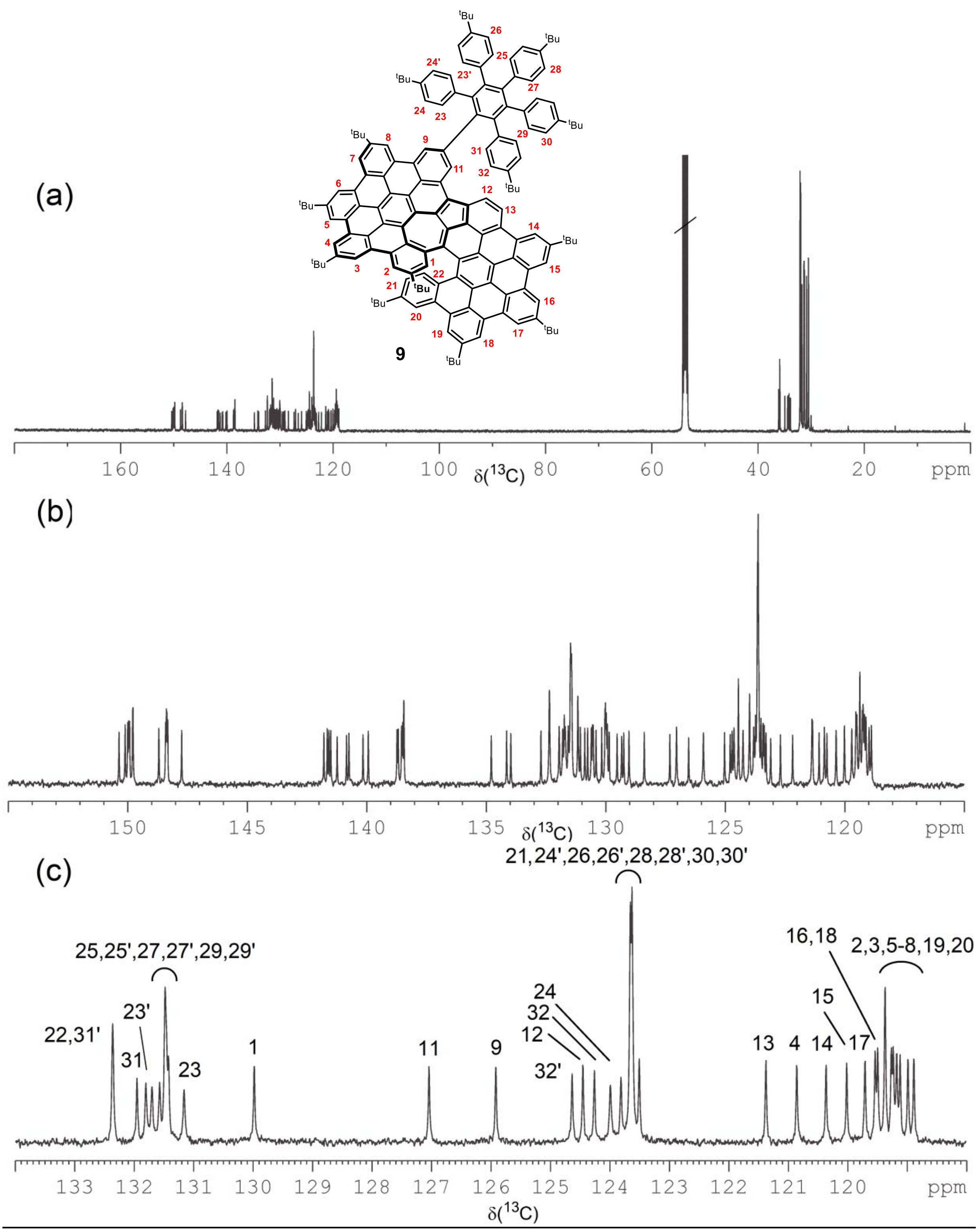

Figure S48. ^{13}C NMR spectrum of **9** (a), expanded region (b) and region of the DEPT135 spectrum (c) in CD_2Cl_2 (125 MHz).

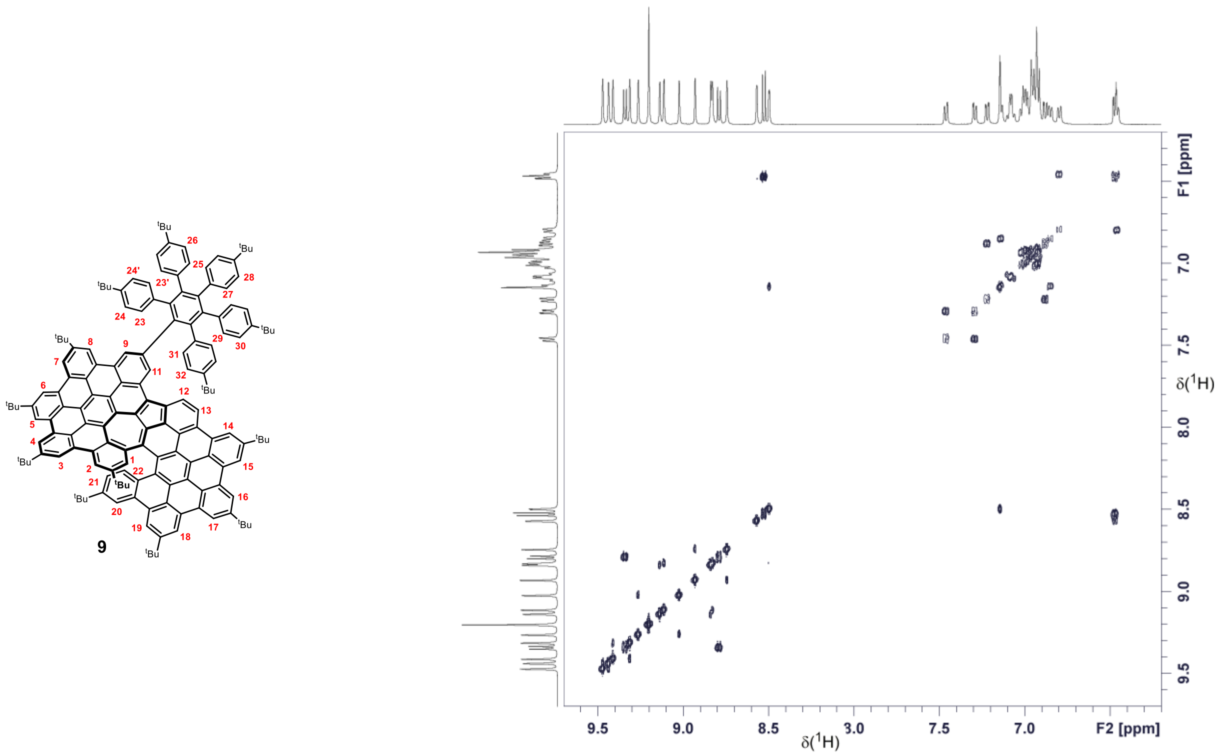

Figure S49. COSY spectrum (region of aromatic protons) of **9** (500 MHz, CD_2Cl_2).

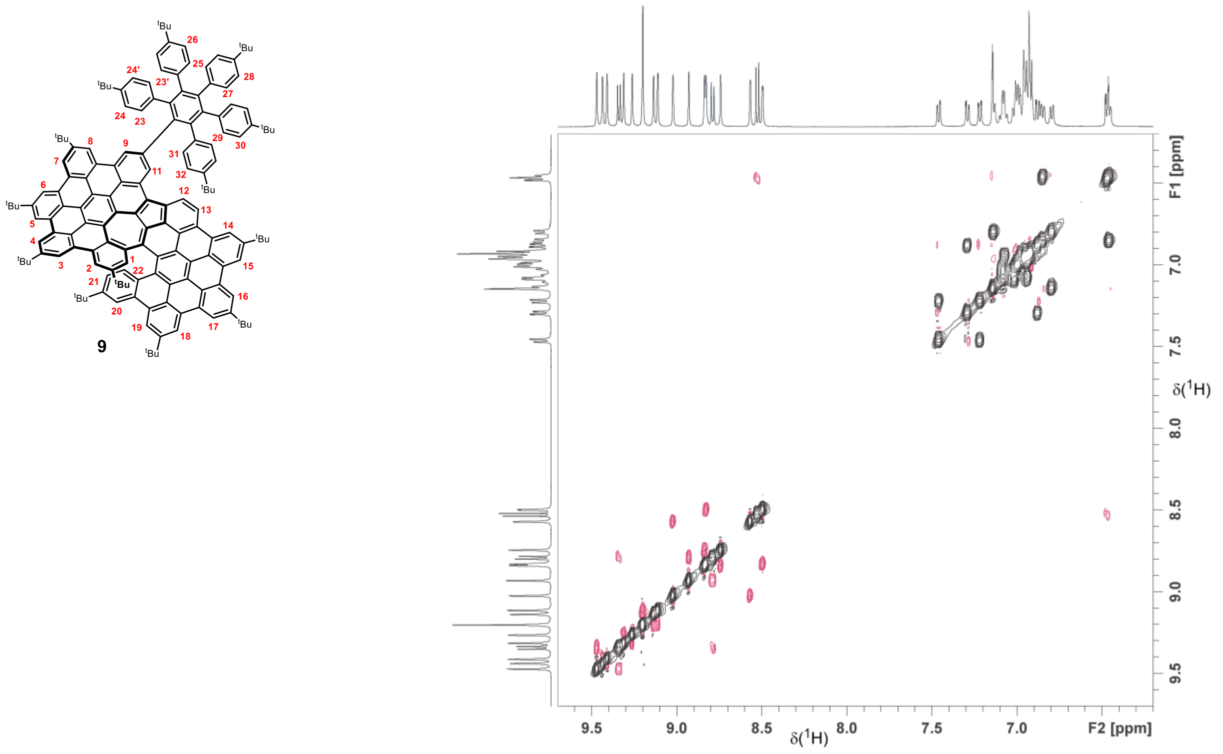

Figure S50. ROESY (red correlations) / EXSY (black correlations) spectrum (region of aromatic protons) of **9** (500 MHz, CD_2Cl_2).

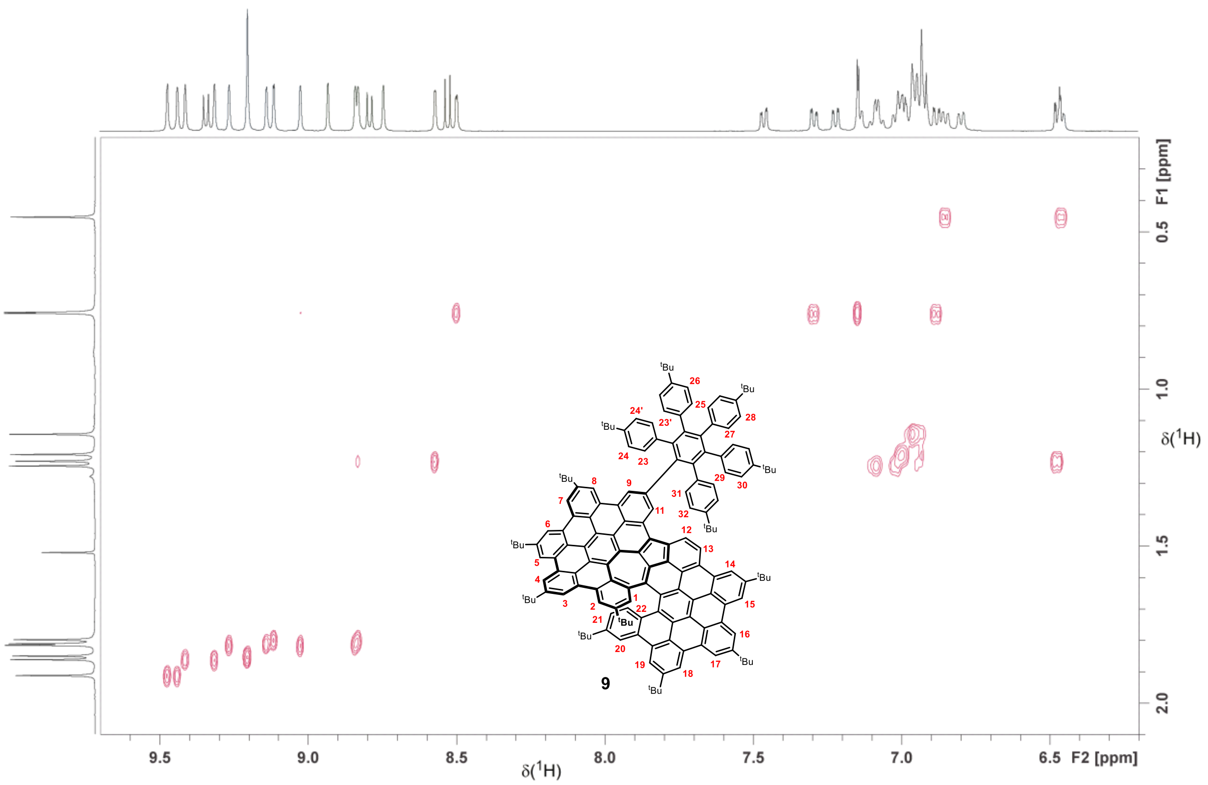

Figure S51. ROESY spectrum (correlations between CH_3 groups and aromatic protons) of **9** (500 MHz, CD_2Cl_2).

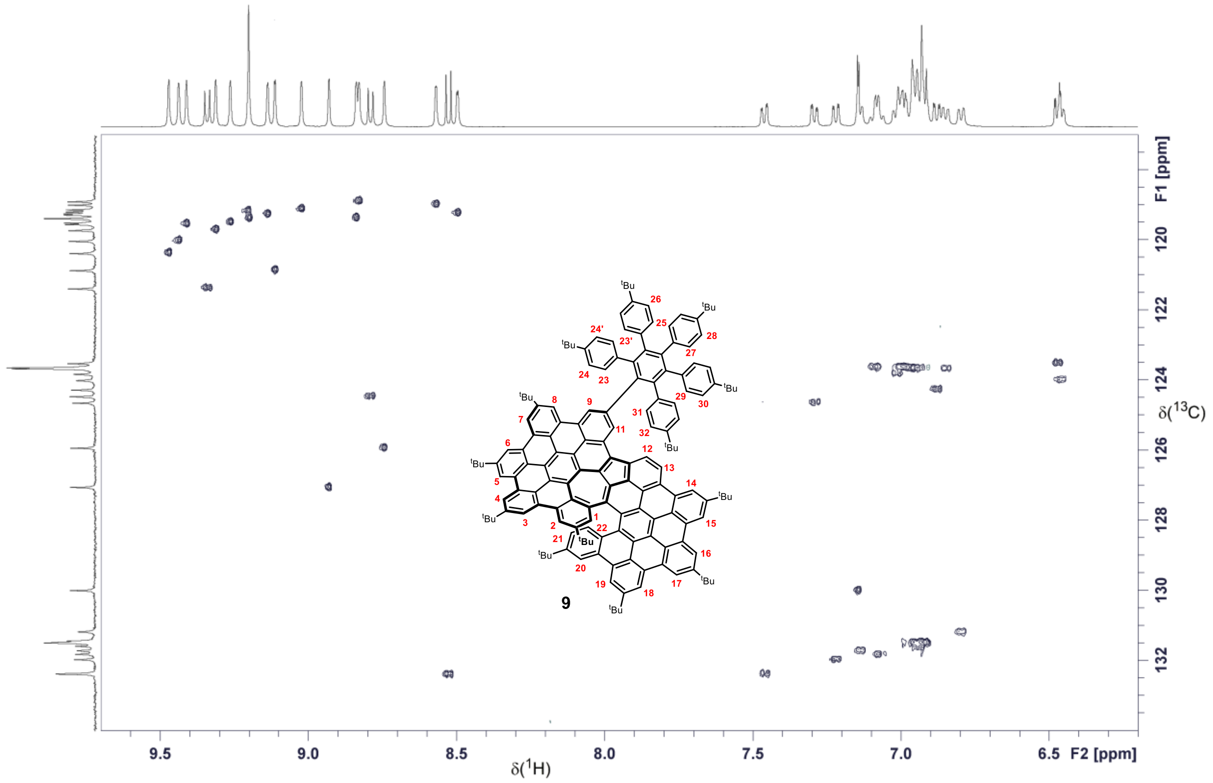

Figure S52. HSQC spectrum (region of aromatic CH groups) of **9** (500 MHz, CD_2Cl_2). The F1 axis depicts the DEPT135 spectrum.

10. References

1. Ma, J.; Fu, Y.; Dmitrieva, E.; Liu, F.; Komber, H.; Hennersdorf, F.; Popov, A. A.; Weigand, J. J.; Liu, J.; Feng, X.,. *Angew. Chem. Int. Ed.* **2020**, *59*, 5637.
2. Mueller, U.; Förster, R.; Hellmig, M.; Huschmann, F. U.; Kastner, A.; Malecki, P.; Pühringer, S.; Röwer, M.; Sparta, K.; Steffien, M.; Ühlein, M.; Wilk, P.; Weiss, M. S., *Eur. Phys. J. Plus* **2015,** *130*, 141.
3. Sparta, K. M.; Krug, M.; Heinemann, U.; Mueller, U.; Weiss, M. S., XDSAPP2.0. *J. Appl. Cryst.* **2016,** *49*, 1085.
4. Kabsch, W., XDS. *Acta Cryst. D* **2010,** *66*, 125.
5. Sheldrick, G., *Acta Cryst. C* **2015,** *71*, 3.
6. Yoshida, Y.; Nakamura, Y.; Kishida, H.; Hayama, H.; Nakano, Y.; Yamochi, H.; Saito, G., *CrystEngComm* **2017,** *19* (26), 3626-3632.
7. Kuroda, R., *JCS PERKIN II* **1982**, *789*.
8. Fuchter, M. J.; Weimar, M.; Yang, X.; Judge, D. K.; White, A. J., *Tetrahedron Lett.* **2012**, *53*, 1108.
9. Gaussian 16, Revision C.01, M. J. Frisch, G. W. Trucks, H. B. Schlegel, G. E. Scuseria, M. A. Robb, J. R. Cheeseman, G. Scalmani, V. Barone, G. A. Petersson, H. Nakatsuji, X. Li, M. Caricato, A. V. Marenich, J. Bloino, B. G. Janesko, R. Gomperts, B. Mennucci, H. P. Hratchian, J. V. Ortiz, A. F. Izmaylov, J. L. Sonnenberg, D. Williams-Young, F. Ding, F. Lipparini, F. Egidi, J. Goings, B. Peng, A. Petrone, T. Henderson, D. Ranasinghe, V. G. Zakrzewski, J. Gao, N. Rega, G. Zheng, W. Liang, M. Hada, M. Ehara, K. Toyota, R. Fukuda, J. Hasegawa, M. Ishida, T. Nakajima, Y. Honda, O. Kitao, H. Nakai, T. Vreven, K. Throssell, J. A. Montgomery, Jr., J. E. Peralta, F. Ogliaro, M. J. Bearpark, J. J. Heyd, E. N. Brothers, K. N. Kudin, V. N. Staroverov, T. A. Keith, R. Kobayashi, J. Normand, K. Raghavachari, A. P. Rendell, J. C. Burant, S. S. Iyengar, J. Tomasi, M. Cossi, J. M. Millam, M. Klene, C. Adamo, R. Cammi, J. W. Ochterski, R. L. Martin, K. Morokuma, O. Farkas, J. B. Foresman, and D. J. Fox, Gaussian, Inc., Wallingford CT, 2016.
10. Geuenich, D.; Hess, K.; Köhler, F.; Herges, R. *Chem. Rev.* **2005**, *105*, 3758.
11. Chen, Z.; Wannere, C. S.; Corminboeuf, C.; Puchta, R.; Schleyer, P. V. R.; *Chem. Rev.* **2005,** *105*, 3842.
12. Schleyer, P. V. R.; Maerker, C.; Dransfeld, A.; Jiao, H.; Eikema Hommes, van N. J. R.; *J. Am. Chem. Soc.* **1996,** *118*, 6317-6318.
